# Tuning the circularly polarized luminescence in homoleptic and heteroleptic chiral Cr^III^ complexes

**DOI:** 10.3389/fchem.2024.1472943

**Published:** 2024-10-09

**Authors:** Maxime Poncet, Céline Besnard, Laure Guénée, Juan-Ramón Jiménez, Claude Piguet

**Affiliations:** ^1^ Department of Inorganic and Analytical Chemistry, University of Geneva, Geneva, Switzerland; ^2^ Laboratory of Crystallography, University of Geneva, Geneva, Switzerland; ^3^ Department of Inorganic Chemistry, University of Granada, Unidad de Excelencia de Química (UEQ), Granada, Spain

**Keywords:** chromium, chirality, emission, circularly polarized luminescence, circular dichroism, chiral HPLC

## Abstract

A series of highly emissive inert and chiral Cr^III^ complexes displaying positive and negative circularly polarized luminescence (CPL) within the near-infrared (NIR) region at room temperature have been prepared and characterized to decipher the effect of ligand substitution on the photophysical properties, more specifically on the chiroptical properties. The helical homoleptic [Cr(dqp-R)_2_]^3+^ (dqp = 2,6-di(quinolin-8-yl)pyridine; R = Ph, ≡-Ph, DMA, ≡-DMA (DMA = *N,N*-dimethylaniline)) and heteroleptic [Cr(dqp)(L)]^3+^ (L = 4-methoxy-2,6-di(quinolin-8-yl)pyridine (dqp-OMe) or L = *N* ^2^,*N* ^6^-dimethyl-*N* ^2^,*N* ^6^-di(pyridin-2-yl)pyridine-2,6-diamine (ddpd)) molecular rubies were synthesized as racemic mixtures and then resolved and isolated into their respective pure *PP* and *MM* enantiomeric forms by chiral stationary phase HPLC. The corresponding enantiomers show two opposite polarized emission bands within the 700–780 nm range corresponding to the characteristic metal-centered Cr(^2^E’→^4^A_2_) and Cr(^2^T_1_^’^→^4^A_2_) transitions with large *g*_lum_ ranging from 0.14 to 0.20 for the former transition. In summary, this study reports the rational use of different ligands on Cr^III^ and their effect on the chiroptical properties of the complexes.

## 1 Introduction

Chiral chromophores displaying intense circularly polarized luminescence (CPL) signal and high CPL brightness (*B*_CPL_) are up-and-coming candidates for applications as molecular probes in biological systems (Carr et al., 2012; Staszak et al., 2019), in bio-imaging (Heffern et al., 2014), in light-emitting devices (e.g., CP-OLEDs) (Brandt et al., 2017; Zinna et al., 2017; Furlan et al., 2024), and in counterfeiting agents (e.g., security inks) (MacKenzie and Pal, 2021). The dissymmetry factor *g*_lum_ is used to estimate the excess of emitted right- or left-circularly polarized light in an isotropic solution. It is deduced from *I*_L_ and *I*_R_, which represent the emission intensities of left and right circularly polarized light, respectively (Equation 1 center, Richardson, 1979; Arrico et al., 2020). This parameter is directly related to the electric dipole ( $\vec{\mu_{ij}}$ ), the magnetic dipole ( $\vec{m_{ij}}$ ) transition moment, and the angle between these two vectors (*θ*) (Equation 1 right).

$$\left|g_{\text{lum}}\right|=\frac{2\times \left(I_{L}-I_{R}\right)}{\left(I_{L}+I_{R}\right)}=4\times \frac{\left(\vec{\mu_{ij}}/\vec{m_{ij}}\right)\times \cos\theta }{{\left(\vec{\mu_{ij}}/\vec{m_{ij}}\right)}^{2}+1}.$$ (1)

An important number of photons must be detected to apply chiral chromophores in CPL materials. To account for that, Zinna and coworkers introduced the CPL brightness (*B*_CPL_), which combines the molar absorption extinction coefficient (*ε*_λexc_), the photoluminescence quantum yield (*ϕ*_PL_), and the dissymmetry factor in Equation 2 (Arrico et al., 2020).

BCPL=ελexc×ϕPL×glum2. (2)

**FIGURE 1:**
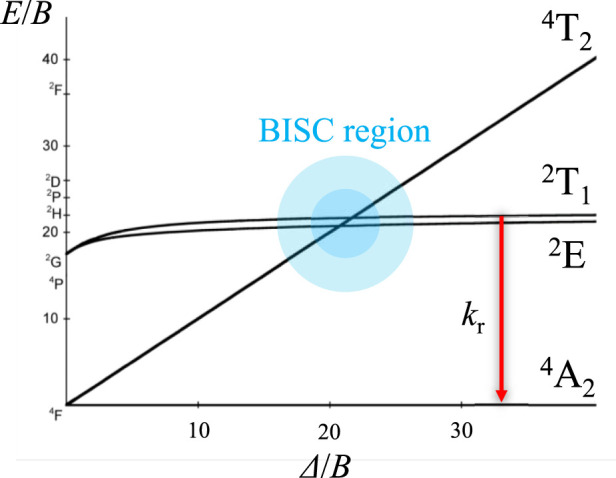
Simplified Tanabe–Sugano diagram for a d^3^ electronic configuration in an octahedral field with *C/B* = 4.5. Two deexcitation pathways are shown: radiative (*k*_r_, red arrow) and back intersystem crossing (BISC, blue domain). A strong LFS prevents the non-radiative deexcitation of the excited state through BISC.

Improving *B*_CPL_ is a current challenge as commonly bright luminescent molecules (displaying high *ε*_λexc_ and *ϕ*_PL_) are usually accompanied with weak *g*_lum_, as rationalized by Equation 1 because intense ED-allowed transitions (ligand-to-metal charge transfer, metal-to-ligand charge transfer, or π* → π transitions) imply $\vec{\mu_{ij}}\gg \vec{m_{ij}}$ , typically in the range of ${\left|\vec{\mu_{ij}}\right|}^{2}\approx 75000\cdot {\left|\vec{m_{ij}}\right|}^{2}$ (Reiné et al., 2018; Albano et al., 2020). Consequently, organic dyes and 4d/5d complexes, which display large *ϕ*_PL_ and *ε*_λexc_, are very challenging systems for improving the *B*_CPL_ due to the limiting weak *g*_lum_ (Saleh et al., 2014; Reiné et al., 2018; Gauthier et al., 2020; Song et al., 2022; Yoshida et al., 2024). Many strategies have been put in place to counter this limitation and to improve the dissymmetry factor, such as (i) the use of supramolecular assemblies (Sang et al., 2020) or (ii) enclosing chiral molecules into liquid crystals (Albano et al., 2020), into excimers (Nakanishi et al., 2016; Homberg et al., 2018; Hara et al., 2019; Mori, 2020), or into cyclic aromatic structures (Sato et al., 2017). The competitor to this type of molecules is lanthanide-based luminescent complexes as, thanks to the primogenic effect, the intrashell ED forbidden/MD allowed character of some emissive f-f transitions brings $\left|\vec{m_{ij}}\right|$ in the range of $\left|\vec{\mu_{ij}}\right|$ , thus maximizing the dissymmetry factor (Equation 1). A large dissymmetry factor often comes with weak luminescence through direct metal center excitation (Lunkley et al., 2008; Nagata and Mori, 2020). For that purpose, intense activity in the field is dedicated to improving *ϕ*_PL_ and *ε*_λexc_ using approaches such as the antenna effect, which avoids the unfavorable direct excitation of the emissive metallic centers (Ward, 2010). Limited by their high cost and, more importantly, by their intrinsic kinetic lability, 4f metal ions could be difficult to handle due to the challenges of maintaining their molecular structures for applications. Chiral 3d metal ions are drawing more attention as an alternative due to their low cost and also because they benefit from the primogenic effect. However, they are limited by (i) their usual kinetic lability, (ii) the lack of efficient Laporte-forbidden d–d emissive states due to weak ligand-field strength, (iii) the mixing of the energy states with ligand-to-metal charge transfers (LMCT)/MLCT, and (iv) the important non-radiative deexcitation pathways that correspond to major handicaps. Cr^III^ complexes in octahedral geometry are exceptions because the large crystal-field stabilization energy (CFSE) found for the 3d^3^ electronic configuration (CSFE = 1.2 Δ_oct_) makes these complexes kinetically inert (Helm and Merbach, 2005; Richens, 2005). In addition, the linear correlation of the Cr(^4^T_2_) energy level with ligand-field splitting (LFS) limits damaging back intersystem crossing (BISC) from the low-lying excited state when strong donor atoms are bound to Cr^3+^ (Figure 1). As a result, this kind of Cr^III^ complex is known to display characteristic long-lived ED-forbidden/MD-allowed metal-centered spin-flip Cr(^2^E→^4^A_2_) and Cr(^2^T_1_→^4^A_2_) NIR emission (Maiman, 1960; Kirk, 1999; Otto et al., 2015; Jimenez et al., 2019; Jiménez et al., 2020; Jimenez et al., 2021; Sinha et al., 2021).

Despite uniting the searched intrinsic kinetic inertness with emissive spin-flip transitions, the design of chiral Cr^III^ complexes with adapted absorption and emission properties remains scarce. Recently, the meridional coordination of bis-terdentate six-membered chelates rings to Cr^III^ has been demonstrated to be an interesting approach for inducing intense spin-flip chiroptical responses (Jimenez et al., 2019; Jimenez et al., 2023). In contrast, introducing chiral centers in the ligand scaffold did not lead to strong CPL emission in related complexes (Poncet et al., 2021; Cheng et al., 2023). In this context, the recent chiral resolution of the inert emissive complexes [Cr(dqp)_2_]^3+^ (dqp = 2,6-di(quinolin-8-yl)pyridine) (Jimenez et al., 2019) and [Cr(ddpd)_2_]^3+^ (ddpd = *N* ^2^,*N* ^6^-dimethyl-*N* ^2^,*N* ^6^-di(pyridin-2-yl)pyridine-2,6-diamine) (Dee et al., 2019) displaying large dissymmetry factors reactivated the interest for this topic. Concomitant high quantum yields could be obtained, making cheap and earth-abundant chromium assemblies promising candidates for CPL applications.

This work follows this strategy with the synthesis of four novel homoleptic complexes [Cr(dqp-R)_2_]^3+^ (R = Ph (**1**), ≡-Ph (**2**), DMA (**3**), and ≡-DMA (**4**), DMA = *N*,*N*-dimethylaniline). The photophysics of these complexes can be compared with those of the heteroleptic complexes [Cr(dqp)(dqp-OMe)]^3+^ (**5**, dqp-OMe = 4-methoxy-2,6-di(quinolin-8-yl)pyridine) and [Cr(dqp)(ddpd)]^3+^ (**6**) (Jimenez et al., 2020) The enantiomeric resolutions proved to be successful for all complexes, which paved the way for addressing their chiroptical properties.

## 2 Results and discussion

**FIGURE 2:**
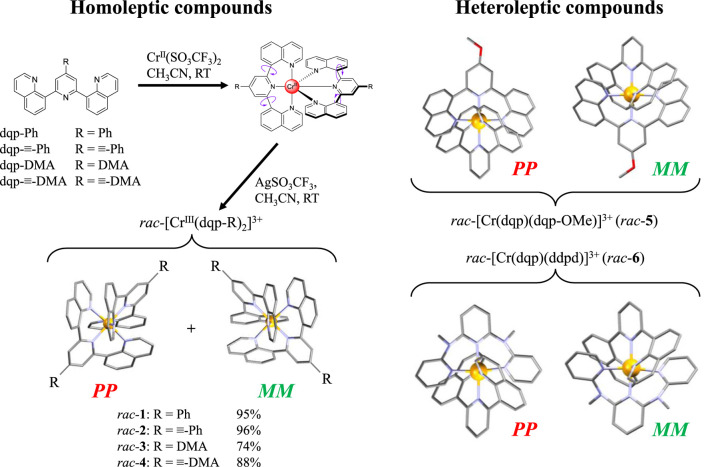
Synthesis of homoleptic complexes *rac*-[Cr(dqp-R)_2_]^3+^. Racemic mixtures of the *PP* and *MM* enantiomers are isolated for both homoleptic and heteroleptic complexes.

The ligands dqp-OMe, ddpd, and dqp; the salt Cr(CF_3_SO_3_)_2_·2H_2_O; and the heteroleptic complexes **5** and **6** were prepared according to published methods (Supplementary Appendix 1; Cantuel et al., 2002; Otto et al., 2015; Jimenez et al., 2019; Jiménez et al., 2020). The preparation of the homoleptic complexes [Cr(dqp-R)_2_]^3+^ is achieved by mixing one equivalent of Cr^II^ precursor and two equivalents of the corresponding ligands dqp-R (dqp-Ph for **1**, dqp-≡-Ph for **2**, dqp-DMA for **3,** and dqp-≡-DMA for **4**) under anaerobic conditions at room temperature. The formed *rac*-[Cr^II^(dqp-R)_2_]^2+^ are oxidized to the kinetically inert *rac*-[Cr^III^(dqp-R)_2_]^3+^ using AgSO_3_CF_3_, affording the desired complexes in good-to-excellent yields (74%–96%, Figure 2).

Single crystals suitable for X-ray diffraction analysis (Supplementary Tables S1–S13; Supplementary Figures S1–S7 in the Supplementary Material) were obtained through slow diffusion of diethyl ether into a concentrated methanol solution for **1**, **2**, **3** (with triflate counterions), and **4** (with chlorine counterions). The dqp derivatives systematically adopt the thermodynamically more stable *meridional* binding around the kinetic labile Cr^II^ intermediate, leading to *meridional* arrangement in the final Cr^III^ complexes after oxidation. Under acidic conditions, the tertiary amine groups in **3** and **4** can be further protonated to yield [Cr(dqp-DMAH)_2_]^5+^ (H_2_-**3**, DMAH = *N*,*N*-dimethylanilininum) and [Cr(dqp-≡-DMAH)_2_]^5+^ (H_2_-**4**). Single crystals suitable for X-ray diffraction of both protonated complexes could also be successfully isolated by crystallization. The semi-flexible nature of the dqp backbone upon *mer* binding joined with the kinetic inertness of Cr^III^ results in the formation of racemic mixtures of helical *PP* and *MM* enantiomers of *D*_2_-symmetry in the crystals (Figure 2). As previously reported, the two instances of intramolecular, interligand π-stacking between the aromatic quinolines produce stabilizing interactions compatible with the exclusive formation of the *PP* and *MM* enantiomers (Jimenez et al., 2021). No meso *PM* complex could be observed, likely due to steric hindrance, as simulated in the parent [Cr(dqp)_2_]^3+^ (Jimenez et al., 2021). To better appreciate the intramolecular interligand π-stacking, the interplanar angle is calculated as the angle between the mean plane of the 10 atoms of each quinoline (Supplementary Figures S8–S15). The closer the angle is to 0, the more parallel to each other the quinolines are. The calculated values for the complexes **1**–**5**, [H_2_-**3**] and [H_2_-**4**] lie within the 15.54°–16.68° range. In the heteroleptic complex [Cr(dqp)(ddpd)]^3+^ (**6**), the corresponding quinoline–pyridine interplanar angle reaches 31.48°, far larger than in the other compounds, but yet smaller than the parent [Cr(ddpd)_2_]^3+^ (46.52°) (Otto et al., 2015). The transoid bite angles N-Cr-N are in the 176.0(1)°–177.7(8)° range for the homoleptic complexes (**1**–**4**, H_2_-**3**, and H_2_-**4**) and in the 175.5(9)°–176.1(10)° range for the heteroleptic complexes (**5** and **6**). In addition, to evaluate the structural distortion with respect to a perfect octahedron, the following parameter $\Sigma =\sum_{i=1}^{12}\left|90-\varphi_{i}\right|$ is computed with *φ*_i_ being the cisoid bite angles N-Cr-N. The distortion in complexes **1**–**6**, H_2_-**3**, and H_2_-**4** ranges from 22.73° ≤ Σ ≤ 31.49°, with the largest distortion in the heteroleptic complex **6**.

**FIGURE 3:**
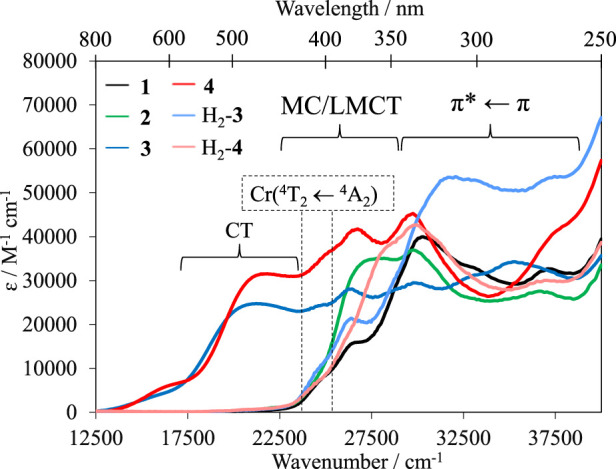
Absorption spectra of complexes **1–4** in H_2_O and H_2_-**3** and H_2_-**4** in aqueous HCl (1 M) at 293 K.

The absorption spectra of the homoleptic complexes (**1**–**4**) were recorded in water at room temperature. Complexes H_2_-**3** and H_2_-**4** were recorded in acidic media (aqueous HCl 1 M) to ensure full protonation of the terminal tertiary amine (Figure 3).

The maxima observed within the 345–250 nm range (29,000–40,000 cm^−1^) are associated with π*←π transitions located on the ligands. Ligand-to-metal charge transfers (LMCTs) are observed at lower energies from 435 nm to 345 nm (23,000–29,000 cm^−1^). Additionally, a shoulder is observed within the 420–400 nm range (23,800–25,000 cm^−1^), which has been assigned to the spin-allowed, parity-forbidden metal-centered (MC) Cr(^4^T_2_←^4^A_2_) transition according to TD-DFT calculations performed on the parent [Cr(dqp)_2_]^3+^ complex (Jimenez et al., 2019). Because *Δ* = *E*(Cr(^4^T_2_←^4^A_2_)) in octahedral complexes, the extracted energy values point to similar ligand-field splitting for all complexes within the 24,272–24,876 cm^−1^ range (Supplementary Table S14). Complexes **3** and **4** display an additional intense broad absorption band in the visible range of the electromagnetic spectrum 800–435 nm (12,500–23,000 cm^−1^) assigned to the intraligand charge transfer (ILCT) from the terminal nitrogen of the aniline to the trivalent chromium center, as observed in related terpyridine-based complexes (Barbour et al., 2017). Ensuring full protonation using acidic conditions (aqueous 1 M HCl) removes the CT band in H_2_-**3** and H_2_-**4** (Figure 3). Moving down in energy and using an increased concentration of complexes, the spin-forbidden/parity-forbidden spin-flip (SF) transitions Cr(^2^T_1_,^2^E←^4^A_2_) can be observed with low molar extinction coefficients ranging from 0.1 M^−1^ cm^−1^ to 0.6 M^−1^ cm^−1^.

**FIGURE 4:**
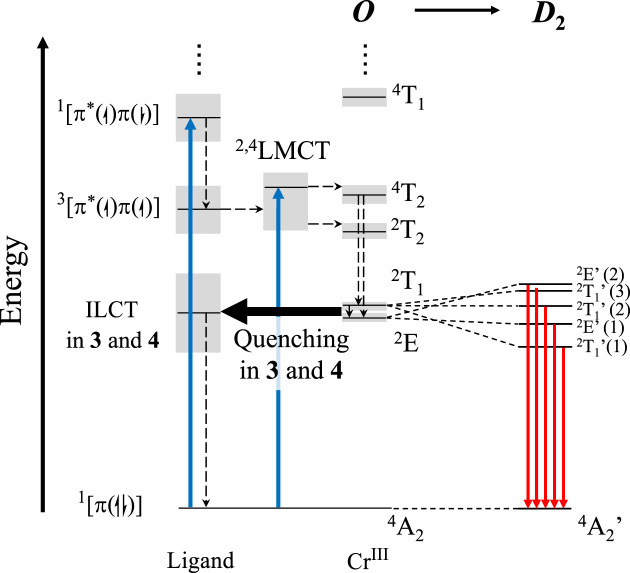
Perrin–Jablonski diagram for a Cr^III^ in *O* geometry. Excitation (blue arrows), internal conversion (dashed arrows), ILCT quenching in **3** and **4** (black arrow), and expected emission in a *D*_2_ distorted geometry (red arrow) are represented. The splitting of the ^2^T_1_ and ^2^E energy levels leads to five expected optically active energy levels in *D*_2_ symmetry.

Because of the slightly distorted geometry going from *O*_h_ to *D*_2_, a splitting of the two expected bands produces five distinct excited energy levels. The Cr(^2^T_1_) splits into three non-degenerated energy levels, and the Cr(^2^E) splits into two (Figure 4; Supplementary Figures S16–S19) (Jimenez et al., 2023). Recording the SF bands was impossible in complexes **3** and **4** because of the overlap with the intense charge transfer absorption band in the visible region of the spectrum. Individual assignments of the absorption bands together with experimental radiative rate constants *k*_rad_, radiative lifetimes *τ*_rad_, oscillator strengths *f*_exp_, and dipole strengths *D*_exp_ are compiled in Supplementary Table S14. From the absorption spectra and the calculated energies of the accessible excited levels, the ligand-field parameter Δ and the Racah parameters *B* and *C* can be estimated using Equations 3–6 (Supplementary Table S15) (Jorgensen, 1963; Witzke, 1971; Chong et al., 2022).

ET24=∆. (3)

ET12=9B+3C−24B2∆. (4)

EE2=9B+3C−50B2∆. (5)

ET22=15B+5C−176B2∆. (6)

ET14=1.5Δ+7.5B−0.5225B2+Δ2−18ΔB. (7)

For all studied complexes, *B* ranges from 611 cm^−1^ to 655 cm^−1^, and *C* ranges from 2,743 cm^–−1^ to 2,885 cm^−1^, which implies a weak impact of the extension of the π-delocalized conjugated system contrariwise to a previously reported substitution of methoxy groups in the same position in the homoleptic [Cr(dqp-OMe)_2_]^3+^ (Jimenez et al., 2021). We note that for the [Cr(dqp)_2_]^3+^, the ratio *C*/*B* equals 4.7, but the ratio is only 3.1 for [Cr(dqp-OMe)_2_]^3+^. Typical values are in the range of 4.2–4.9 and are sometimes assumed to be 4.7 (Adachi, 2024).

**FIGURE 5:**
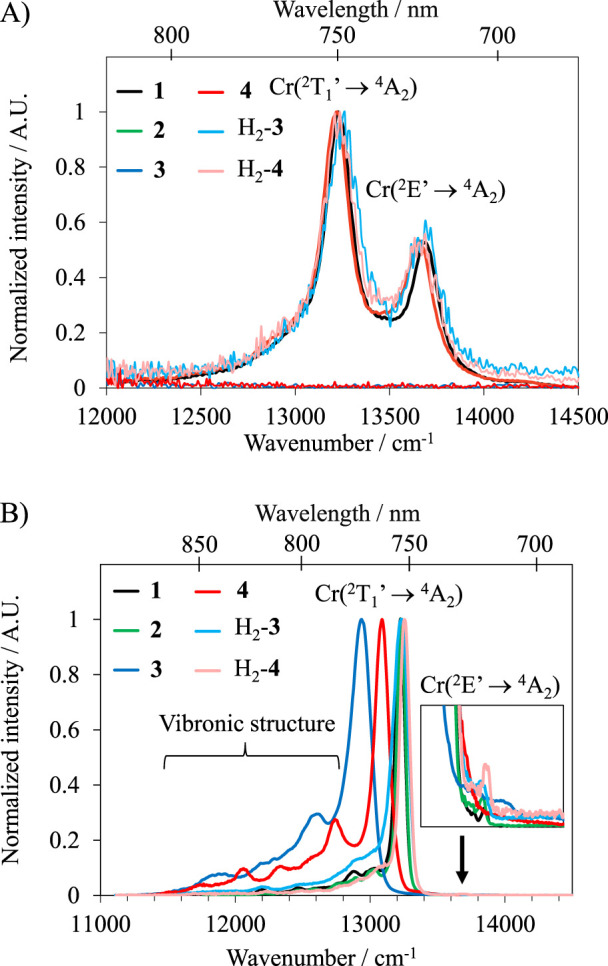
Near-infrared emission spectra with *λ*_exc_ = 435 nm of **(A)** Complexes **1–4** in H_2_O and H_2_-**3** and H_2_-**4** in aqueous 1 M HCl at 293 K and **(B)** Complexes **1–4** in DMSO/H_2_O 1:1 and H_2_-**3** and H_2_-**4** in aqueous 1 M HCl at 77 K.

Upon UV–VIS excitation (λ_exc_ = 350–435 nm) at room temperature, the typical sharp NIR dual emissions (FWHM ≈200 cm^−1^) observed in complexes **1** and **2** (Figure 5A) can be assigned to the radiative relaxation of the Cr(^2^E) and Cr(^2^T_1_) excited state levels to the Cr(^4^A_2_) ground state level in approximate *O* symmetry (Jimenez et al., 2019; Jimenez et al., 2021). The most intense band is attributed to the Cr(^2^T_1_’→^4^A_2_) SF transition (maxima 13,227 cm^−1^ (756 nm) in **1** and **2**) and the less intense to Cr(^2^E’→^4^A_2_) (maxima at 13,698 cm^−1^ (730 nm) for **1** and 13,661 cm^−1^ (732 nm) for **2**). Contrariwise, the luminescence of complexes **3** and **4** is completely quenched, likely due to energy back transfer into the non-emissive CT levels (Figure 4). Upon protonation of the anilines (H_2_-**3** and H_2_-**4**), a weak luminescence is retrieved with an overall photoluminescence quantum yield of *Φ*_PL_ ≤ 0.0011% (Supplementary Table S16; Figure 5A), in contrast to the non-luminescent terpyridine analog (Barbour et al., 2017).

Upon changing the temperature, the Boltzmann distribution of the thermally equilibrated Cr(^2^E’) and Cr(^2^T_1_’) levels is modified, and 77 K measurements result in a close to single emission assigned to Cr(^2^T_1_’→^4^A_2_) (Figure 5B). Interestingly, the total (**3** and **4**) and partial (H_2_-**3** and H_2_-**4**) quenching pathways of the luminescence happening at 293 K vanish at 77 K, and strong luminescence is recovered (Figure 5B). For both H_2_-**3** and H_2_-**4** at 77 K, the main emission band is blue-shifted compared to the non-protonated **3** and **4** complexes. The room temperature *Φ*_PL_ values determined in aerobic and anaerobic conditions (CH_3_CN, *λ*_exc_ = 435 nm) are gathered in Supplementary Table S16. The obtained values in oxygen-free solutions are 12.4%, making complexes **1** and **2** in the same range as the previously reported record-holding deuterium-free Cr^III^ complexes (Jimenez et al., 2019; Jimenez et al., 2021). The origin of the high *Φ*_PL_ observed is attributed to (i) the weak trigonal distortion forming the octahedral geometry, preventing the non-radiative deexcitation pathways (Kitzmann et al., 2022) and (ii) the strong LFS induced by the dqp-type ligands, avoiding BISC to the Cr(^4^T_2_) excited state level (Figure 1).

Time-resolved experiments were conducted and displayed mono-exponential decays, resulting in τCr,obsE2′,T21′ > 1 ms in the deaerated solution at 293 K and up to 2.62(2) ms at 77 K (**2**, Supplementary Table S16; Supplementary Figures S20–S25). Air-equilibrated experiments demonstrated the extreme dependence on dissolved dioxygen, as previously reported for Cr^III^ chromophores ( τCr,obsE2′,T21′ < 65 µs) (Kirk, 1999). For more insight, the reader is referred to the in-depth analysis of the mechanism discussed by Alazaly et al. (2023).

Additionally, the rate of energy transfer from Cr(^2^E’/^2^T_1_’) to O_2_, *k*_q_, can be estimated in **1** and **2** with the relationship *k*_q_ = 1/[O_2_]⋅(1/τ_air_ −1/τ_Ar_) (Burgin et al., 2022), in which [O_2_] is the oxygen concentration in the solvent at the experimental temperature (2.42 mM in CH_3_CN at 293 K), τ_air_ is the Cr(^2^E’/^2^T_1_’) lifetime under air-equilibrated conditions, and τ_Ar_ is the respective lifetime under deaerated conditions. The obtained values of 6.23∙10^6^ s^−1^ (**1**) and 7.59∙10^6^ s^−1^ (**2**) demonstrate the effectiveness of O_2_ quenching (Supplementary Table S16).

The thermal equilibrium of the Cr(^2^T_1_’) and Cr(^2^E’) levels was confirmed by the recording of identical excited state lifetimes at both maxima. The sensitization efficiency for transferring the energy from the ligand to the Cr(^2^E’/^2^T_1_’) excited state $\eta_{\text{sens}}^{L\to \text{Cr}}$ is found to be above 71% (Supplementary Table S16). The measurement of certain photophysical properties in **3**, **4**, H_2_-**3**, and H_2_-**4** was limited by (i) the weak emitted signals (H_2_-**3** and H_2_-**4**) that prevented reliable measurements of the excited state lifetimes and (ii) the lack of emissive properties in **3** and **4**. Nevertheless, the retrieval of the luminescence at 77 K allowed time-resolved measurements on all complexes. Excitation spectra were recorded in dilute solutions and closely match the absorption spectra of the corresponding compounds (Supplementary Figures S26–S29).

**FIGURE 6:**
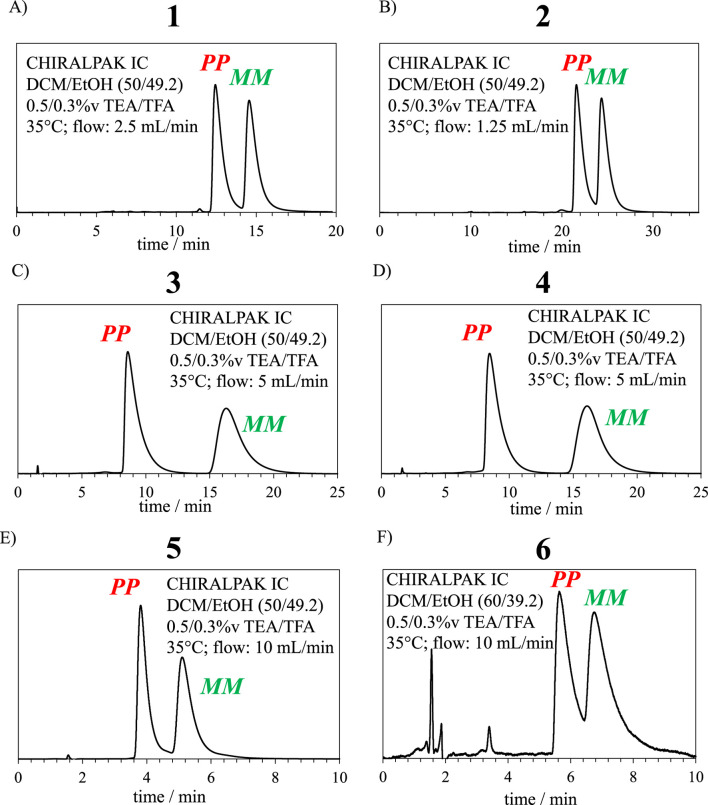
CSP-HPLC conditions required for the enantiomeric resolution of complexes **(A) 1**, **(B) 2**, **(C) 3**, **(D) 4**, **(E) 5**, and **(F) 6**.

Chiral stationary phase high-performance liquid chromatography (CSP-HPLC) was proven to be effective, straightforward, and quick in the separation of the racemic mixture of d-block complexes such as Ru^III^, Cr^III^, and Co^III^ (Yoshida et al., 2013; Cortijo et al., 2017; Dee et al., 2019). Isocratic elution using a CH_2_Cl_2_/CH_3_CH_3_OH/triethylamine/trifluoroacetic acid 50/49.2/0.5/0.3 (v:v) mixture resulted in the separation of the complexes **1–6** (Figures 6A–F). For complexes **1**, **2**, and **5**, the elutions of *MM* enantiomers tail and slightly overlap with the elution peaks of the *PP* enantiomers. As a result, a small amount of contamination was observed upon reinjection in the analytical column (Supplementary Figures S30–S32). Nevertheless, thorough integrations of the chromatogram reveal that ≤0.5% mol was present in the sample. Therefore, the chiroptical studies were carried out while considering these contaminations as negligible. The obtention of a mirror image of the circular dichroism (CD) and CPL signals confirmed the adequacy of the latter assumption. Note that the efficient separation of **3** and **4** would allow a larger scale separation.

**FIGURE 7:**
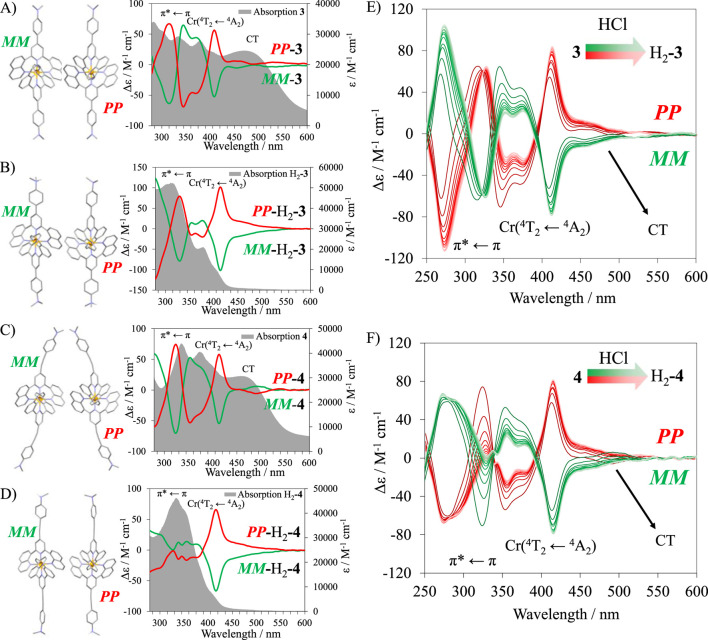
Crystalline structures of *PP* and *MM* enantiomers and the corresponding absorption (gray traces) and CD spectra (red and green traces) for **(A) 3**, **(B)** H_2_-**3**, **(C) 4**, and **(D)** H_2_-**4**. CD titration of complexes **(E) 3** to H_2_-**3** and **(F) 4** to H_2_-**4** in aqueous media.

**FIGURE 8:**
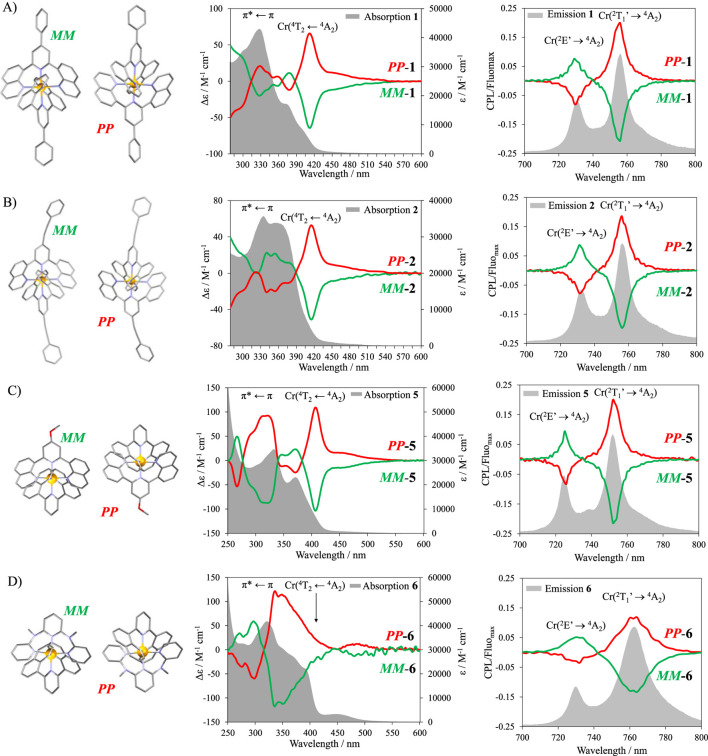
Crystalline structure of the *PP* and *MM* enantiomers and the corresponding CD (middle, red, and green traces) and CPL (λ_exc_ = 340 nm, right, (red and green traces)) spectra for **(A) 1**, **(B) 2**, **(C) 5**, and **(D) 6**. The experimental bandwidth (EBW) for the CPL spectra in A–C is 0.5 nm and 5 nm for **(D)**. The gray traces show the absorption (middle) and emission (right) spectra.

The CD was recorded, and mirror images were systematically obtained for all complexes **1**–**6**, H_2_-**3**, and H_2_-**4** (Figures 7, 8). The study of the analogous crystallized *MM*-[Cr(dqp)_2_]^3+^ in CD allowed the assignment of the measured complexes (Jimenez et al., 2019). In all complexes except for **6** (Figure 8D), a strong Cotton effect could be observed in the 410–430 nm range, corresponding to the MC Cr(^4^T_2_←^4^A_2_) transition. The UV range is also dominated by considerable Cotton effect matching with π*←π transitions located on the ligands. Despite the significant absorption of the CT bands in complexes **3** and **4** (ε = 25,000–32000 M^−1^cm^−1^), little to no Cotton effect was observed, confirming the low *g*_abs_ for these specific transitions (Figures 7A, C). Because **3** and **4** could be protonated in solution, their conversion to the respective protonated species H_2_-**3** and H_2_-**4** could be followed by CD upon successive addition of hydrochloric acid (0.05 M) in the aqueous solution (Figures 7E, F). The MC ligand-field Cr(^4^T_2_←^4^A_2_) transition is less affected by the protonation of the ligands than the π*←π transitions, which display important changes in the 250–400 nm range.

Circularly polarized luminescence (CPL) measurements were recorded on each enantiomer of the emissive complexes **1**, **2**, **5**, and **6** (Figures 8A–D). The low photoluminescence quantum yield of H_2_-**3** and H_2_-**4** and the non-emissive character of **3** and **4** prevented CPL measurements. To ensure a correct determination of the dissymmetry factor, the highest resolution of the CPL spectra is ensured with the smallest emission slit aperture settings available at the cost of the signal intensity (Sickinger et al., 2024). The experimental bandwidth (EBW) used in these experiments is 0.5 nm for **1**, **2**, and **5** and 5 nm for **6**. A better signal-to-noise ratio can be obtained by opening the slits (larger EBW), but the resolution of the spectra must be sacrificed, skewing the results and the value of *g*_lum_. It is, therefore, more correct to measure the sample at the smallest slit aperture available and divide the CPL spectra by the maximum read value of the corresponding emission. Under unpolarized excitation (λ_exc_ = 340 nm), complexes **1**, **2**, **5**, and **6** (Figures 8A–D) displayed strong circularly polarized emission within the 720–780 nm range. The dissymmetry factors |*g*_lum_| obtained for the Cr(^2^E’→^4^A_2_) transition reach 0.07–0.08 for all four complexes (Supplementary Figures S33–S36). Interestingly, for the Cr(^2^T_1_’→^4^A_2_) transition, complexes **1**, **2**, and **5** display |*g*_lum_| = 0.20, whereas complex **6** reaches only |*g*_lum_| = 0.14.

Putting these results in perspective with those of the previously reported homoleptic complexes of [Cr(ddpd)_2_]^3+^ and [Cr(dqp-R)_2_]^3+^ (R = H, Br, OMe, C≡CH, Table 1) (Otto et al., 2015; Jimenez et al., 2019; Jimenez et al., 2021), a trend emerges for the Cr(^2^T_1_’→^4^A_2_) transitions. Indeed, all complexes containing two dqp-based ligands display the same dissymmetry factor of 0.2, whereas the more flexible [Cr(ddpd)_2_]^3+^ culminated at 0.093 (Dee et al., 2019). By exploiting the chemical inertness of Cr^III^, one of each ligand could be implemented in the same complex to give the heteroleptic complex [Cr(dqp)(ddpd)]^3+^ (**6**), for which |*g*_lum_| = 0.14 corresponds to the average of the dissymmetry factors of the two parent homoleptic complexes. As an attempt to rationalize this result, a hypothetical “ligand dissymmetry factor” $g_{\text{lum}}^{L}$ could be imagined for a given transition defined as Equation 8.

$$\left|g_{\text{lum}}^{L}\right|=\frac{\eta^{L}}{n}\cdot \left|g_{\text{lum}}^{\text{total},\text{homoleptic}}\right|.$$ (8)

$\eta^{L}$ is the denticity of the ligand, n the number of available coordination sites around the metal center (6 for octahedral symmetry), and $g_{\text{lum}}^{\text{total},\text{homoleptic}}$ is the dissymmetry factor of the corresponding homoleptic complex of a specific transition. Therefore, in the cases of [Cr(dqp)_2_]^3+^ and [Cr(ddpd)_2_]^3+^, the following values are obtained: $g_{\text{lum}}^{\text{dqp}}=0.1$ and $g_{\text{lum}}^{\text{ddpd}}=0.0465$ . From these values, the dissymmetry factor of a heteroleptic complex could be estimated by applying Equation 9.

$$\left|g_{\text{lum}}^{\text{total},\text{heteroleptic}}\right|=\sum \left|g_{\text{lum}}^{L}\right|.$$ (9)

**TABLE 1:** CPL brightness (*B*_CPL_) calculation for each emissive transition in complexes **1**, **2**, **5**, and **6**.

Complex	ε/M^−1^cm^−1^	*ϕ*_PL_/% ^(^ ^a^ ^)^	^2^T_1_’/^2^E’ ratio		|*g*_lum_|	*B*_CPL_/M^−1^cm^−1^
**1**	34,622 ^(^ ^b^ ^)^	12.4	^2^E’	0.324	8⋅10^−2^	56
			^2^T_1_’	0.676	2⋅10^−1^	290
**2**	36,118 ^(^ ^b^ ^)^	12.4	^2^E’	0.333	8⋅10^−2^	60
			^2^T_1_’	0.667	2⋅10^−1^	299
**5**	29,881^(^ ^b^ ^)^	6.5	^2^E’	0.308	8⋅10^−2^	24
			^2^T_1_’	0.692	2⋅10^−1^	134
**6**	32,684 ^(^ ^b^ ^)^	6.0	^2^E’	0.181	7⋅10^−2^	14
			^2^T_1_’	0.819	1.4⋅10^−1^	112
[Cr(dqp)_2_]^3+ (^ ^c^ ^)^	20,000 ^(^ ^d^ ^)^	5.2	^2^T_1_’	-	2⋅10^−1^	104
[Cr(dqp-Br)_2_]^3+ (^ ^e^ ^)^	10,591 ^(^ ^f^ ^)^	14	^2^T_1_’	-	1.9⋅10^−1^	140
[Cr(dqp-OMe)_2_]^3+ (^ ^e^ ^)^	5,000 ^(^ ^f^ ^)^	17	^2^T_1_’	-	1.8⋅10^−1^	76.5
[Cr(dqp-≡)_2_]^3+ (^ ^e^ ^)^	13,553 ^(^ ^f^ ^)^	15	^2^T_1_’	-	1.7⋅10^−1^	173
[Cr(ddpd)_2_]^3+ (^ ^g^ ^)^	30,000 ^(^ ^h^ ^)^	11	^2^T_1_’	-	9.3⋅10^−2^	153

^a^ Deaerated conditions.

^b^ λ_abs_ = 340 nm.

^c^ Jimemez et al., 2019.

^d^ λ_abs_ = 370 nm.

^e^ Jimemez et al., 2021.

^f^ λ_abs_ = 405 nm.

^g^ Dee et al., 2019.

^h^ λ_abs_ = 300 nm.

For **2**, one obtains $g_{\text{lum}}^{\text{dqp}}+g_{\text{lum}}^{\text{ddpd}}=0.1+0.0465=0.1465$ , which corresponds closely to the experimental value of 0.14, verifying the hypothesis. Unfortunately, the number of available compounds is limited; thus, to validate or invalidate the statement, more flexible tridentate ligands must be studied. As a general conclusion to the chiroptic luminescence, an extension of the organic π-delocalized electronic cloud in the *para* position of the pyridine does not influence the dissymmetry factor, while the implementation of ddpd ligands results in an attenuated *g*_lum_. A plausible explanation for this observation is that the rigidification of the complex is key in the obtention of a large dissymmetry factor, and the rigidity must be maximized.

The *B*_CPL_ of the emissive complexes can be calculated using Equation 2. Because the *g*_lum_ values associated with the two observed emissions Cr(^2^T_1_’→^4^A_2_) and Cr(^2^E’→^4^A_2_) are of opposite signs in the same enantiomer, *B*_CPL_ must be similarly split into two components. Accordingly, the *ϕ*_PL_ must be split relative to the intensity of each band at the temperature measurement (293 K). The emission spectrum of the corresponding complex is approximated as two Gaussian curves, deconvoluted as such, and the ratio between them is calculated (idealized Cr(^2^T_1_→^4^A_2_) and Cr(^2^E→^4^A_2_) transitions, Supplementary Figures S37–S40). The calculated values of *B*_CPL_ are compiled in Table 1 and range from 299 M^−1^cm^−1^ to 122 M^−1^cm^−1^ for the Cr(^2^T_1_’→^4^A_2_) transition and 60–14 M^−1^cm^−1^ for the Cr(^2^E’→^4^A_2_) transition, which are among the highest reported values for CPL active compounds (Arrico et al., 2020). A higher *B*_CPL_ can be achieved by exciting the maxima of the absorption band to increase the value of ε in the *B*_CPL_ calculation. The *ϕ*_PL_ is considered invariant with the excitation wavelength.

## 3 Conclusion

A series of new chiral homoleptic and heteroleptic Cr^III^ chromophores with dqp-based ligands with *para* functionalization of the central pyridine have been synthesized and characterized. The addition of *N*,*N*-dimethylaniline to the complex resulted in a large increase in absorbance in the VIS region (CT) accompanied by quenching of the luminescence. Interestingly, weak luminescence is retrieved upon protonation of the aniline (*Φ*_PL_ ≤ 0.0011%). The highly luminescent complexes [Cr(dqp-Ph)_2_]^3+^ and [Cr(dqp-≡-Ph)_2_]^3+^ are promising candidates for use as chiral luminescent probes. Enantiomeric resolution of all six racemic complexes could be achieved by CSP-HPLC. Implementing *N*,*N*-dimethylaniline as a substituent to the complex resulted in a baseline separation of the two enantiomers, allowing a potential large-scale separation. Moreover, the evolution of the circular dichroism from the non-protonated to the protonated species in [Cr(dqp-DMA)_2_]^3+^/[Cr(dqp-DMAH)_2_]^5+^ and [Cr(dqp-≡-DMA)_2_]^3+^/[Cr(dqp-≡-DMAH)_2_]^5+^ could be followed. Near-perfect octahedral geometries are obtained with the help of the six-membered chelate rings, providing long excited state lifetime and high overall photoluminescence quantum yields at room temperature. Dual circularly polarized luminescence arises from the Cr(^2^T_1_’) and Cr(^2^E’) excited level to the Cr(^4^A_2_) ground state within the 720–780 nm range. The observed |*g*_lum_| for the emissive complexes amounts to 0.2 except for [Cr(dqp)(ddpd)]^3+^, for which the |*g*_lum_| was measured to be in between the two corresponding parent homoleptic complexes [Cr(dqp)_2_]^3+^ (|*g*_lum_| = 0.2) and [Cr(ddpd)_2_]^3+^ (|*g*_lum_| = 0.093). High *B*_CPL_ values, ranging from 299 ^−1^cm^−1^ to 122 M^−1^cm^−1^ for the Cr(^2^T_1_’→^4^A_2_) transition and 60–14 M^−1^cm^−1^ for the Cr(^2^E’→^4^A_2_) transition, were obtained, reaching the typical range of 4f-based chiral chromophores with the added value of the inertness and low cost of trivalent chromium.

## Data Availability

The original contributions presented in the study are included in the article/Supplementary Material, further inquiries can be directed to the corresponding authors.
