# Supplementary material for: Tuning the circularly polarized luminescence in homoleptic and heteroleptic chiral CrIII complexes
Source: Front Chem. 2024 Oct 9;12:1472943. doi: 10.3389/fchem.2024.1472943 (PMC11496276; doi:10.3389/fchem.2024.1472943)
Supplement: Supplementary file 1 [file DataSheet1.PDF]

Tuning the Circularly Polarized Luminescence (CPL) in homoleptic and heteroleptic chiral Cr^{III} complexes.

Maxime Poncet,[1] Céline Besnard,[2] Laure Guénée,[2] Juan-Ramón Jiménez,*[3] and Claude Piguet*[1].

[1] Department of Inorganic and Analytical Chemistry, University of Geneva, quai E. Ansermet 30, 1211 Geneva 4, Switzerland;

[2] Laboratory of Crystallography, University of Geneva, quai E. Ansermet 24, 1211 Geneva 4, Switzerland

[3] Department of Inorganic Chemistry, University of Granada, Unidad de Excelencia de Química (UEQ), Avda. Fuentenueva, 18071 Granada, Spain;

Supporting Information

(57 pages)

Appendix 1: Experimental section.

Solvents and starting materials.

Reagent grade acetonitrile (ACN) was distilled from CaH_2. All other chemicals were purchased from commercial suppliers and used without further purification. Silica-gel plates (Merck, 60 F254) were used for thin-layer chromatography and preparative column chromatography was performed using SiliaFlash® silica gel P60 (0.04-0.063 mm).

Spectroscopic and analytical measurements.

^{1}H and ^{13}C NMR spectra were recorded at 298 K on a Bruker Avance 400 MHz spectrometer. Spectrophotometric titrations were performed with a J&M diode array spectrometer (Tidas series) connected to an external computer. Pneumatically-assisted electrospray (ESI) mass spectrum was recorded on an Applied Biosystems API 150EX LC/MS System equipped with a Turbo Ionspray source®. High Resolution Mass Spectra were recorded on a Xevo G2-TOF HRMS instrument equipped with a Zspray™ Lockspray™ ESI/APCI/ESCi® electrospray by Waters™. Elemental analyses were performed by K. L. Paglia from the Microchemical Laboratory of the University of Geneva. Solution state absorption spectra were recorded using a Lambda 1050 Perkin Elmer spectrometer (quartz cell path length 1 cm, 1 mm or 0.2 mm, 250-1600 nm domain). Emission spectra (excitation at 355 nm) and excitation spectra were recorded, with a Fluorolog (Horiba Jobin-Yvon), equipped with iHR320, a Xenon lamp 450-Watt Illuminator (FL-1039A/40A), a water-cooled photo multiplier tube (PMT Hamamatsu R2658 or R928) for the 250-850 nm range. Both detectors are corrected for the spectral response of the system. Emission spectra (excitation at 730 nm) was recorded with a MDL-III-730-1.5W as light source connected to a PSU-III-LED power supply. For time-resolved experiments, the decay curves were recorded from previously excited samples, with a photomultiplier (Hamamatsu R2658 or R928 or Hamamatsu IR PMT H10330-75) and a digital oscilloscope (Tektronix MDO4104C). Pulsed excitation at 355 nm was obtained with the third harmonic of a pulsed Nd:YAG laser (Quantel Qsmart 850).

Crystallographic measurements.

Summary of crystal data, intensity measurements and structure refinements for complexes *rac*-[Cr(dqp-Ph)$_2$](SO_3CF_3)$_3$ (**1**), *rac*-[Cr(dqp-≡-Ph)$_2$](SO_3CF_3)$_3$ (**2**), *rac*-[Cr(dqp-DMA)$_2$](SO_3CF_3)$_3$ (**3**), *rac*-[Cr(dqp-DMAH)$_2$](SO_3CF_3)$_5$ (H_2-**3**), *rac*-[Cr(dqp-≡-DMA)$_2$]Cl_3 (**4**) and *rac*-[Cr(dqp-≡-DMA)$_2$](SO_3CF_3)$_5$ (H_2-**4**) are collected in Tables S1-S13 with pertinent bond lengths, bond angles. ORTEP views with pertinent numbering schemes are gathered in Figures S1-S7. The crystals were mounted on Hampton cryoloops with protection oil. X-ray data collections were performed with a XtaLAB Synergy-S diffractometer equipped with a hybrid pixel array "hypix arc 150" detector. The structures were solved by using the dual-space methods in SHELXT. [A1-5]. Full-matrix least-square refinements on F^2 were performed using SHELXL[A1-6] within the Olex2 software.[A1-7] CCDC 2373726-2373731 contain the supplementary crystallographic data for this paper. These data can be obtained free of charge from The Cambridge Crystallographic Data Centre via https://www.ccdc.cam.ac.uk/structures/.

Synthetic procedure for the preparation of dqp-R (R = Ph, ≡-Ph, DMA, ≡-DMA (DMA = *N,N*-dimethylaniline)).

8,8'-(4-bromopyridine-2,6-diyl)diquinoline (dqp-Br) and 8,8'-(4-methoxypyridine-2,6-diyl)diquinoline (dqp-OMe) were synthesised according to published literature.[A1-8]

Synthesis of 8,8'-(4-phenylpyridine-2,6-diyl)diquinoline (dqp-Ph).

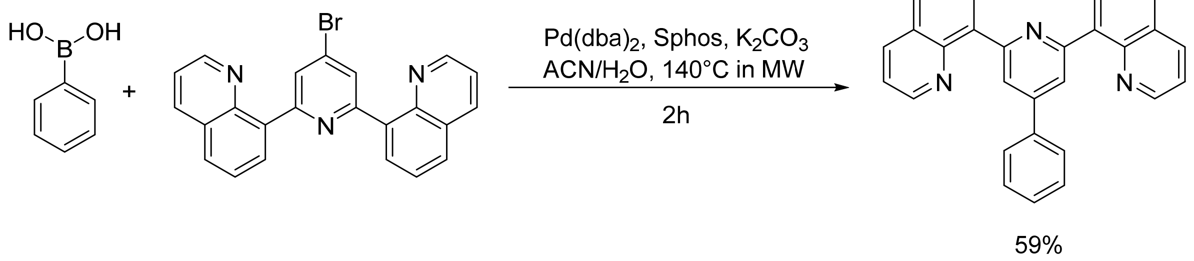

8,8'-(4-bromopyridine-2,6-diyl)diquinoline (133 mg, 0.32 mmol, 1.0 eq.), Phenyl boronic acid (44.6 mg, 0.37 mmol, 1.1 eq.), [$Pd_2(dba)_3$] (dba = dibenzylideneacetone) (13.1 mg, 0.02 mmol, 0.07 eq.), 2-dicyclohexylphosphino-2',6'-dimethoxybiphenyl (SPhos) (18.1 mg, 0.04 mmol, 0.14 eq.), K_2CO_3 (176.6 mg, 1.28 mmol, 3.96 eq.) in a mixture of acetonitrile:water (10 mL 3:1) were loaded into a microwave vial. The solution was bubbled with N_2 for about 10 min before being put under the MW for 2h at 140°C. After the reaction, the vial was left to cool down at RT. The ACN phase was yellow/orange and the aqueous phase colourless. The mixture was put in a separatory funnel and water and EtOAc were added (50 mL). The organic phase was extracted with water (3 x 100 mL). The organic fraction was dried over Na_2SO_4 and evaporated to dryness. Cold isopropanol (5 mL) was used to solubilise the impurity and filter-out 8,8'-(4-phenylpyridine-2,6-diyl)diquinoline (**dqp-Ph**) (78mg, 0.19 mmol, 59%). ^{1}H NMR ($CDCl_3$, 400 MHz): δ (ppm): 7.47 (m, 5H); 7.69 (t, 2H); 7.84 (d, 2H); 7.91 (dd, 2H); 8.26 (dd, 2H); 8.34 (m, 4H); 9.02 (dd, 2H). ESI-MS (CH_3CN) m/z: [**dqp-Ph**+H]$^+$ calc: 410.2, found: 410.2.

Synthesis of 8,8'-(4-(phenylethynyl)pyridine-2,6-diyl)diquinoline (dqp-≡-Ph).

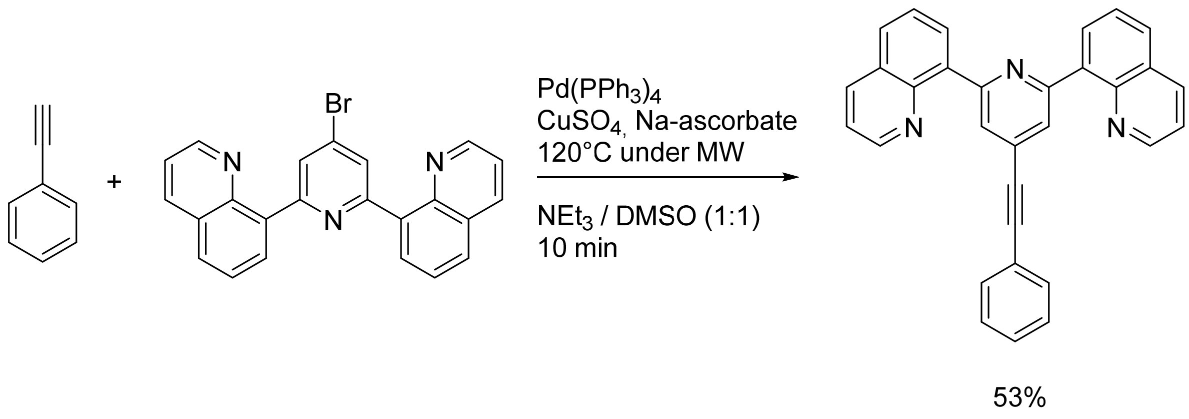

A mixture of NEt_3 and DMSO (1:1, 10mL total) was put into the microwave vial and was bubbled with N_2 for 5-10 min. 8,8'-(4-bromopyridine-2,6-diyl)diquinoline (250 mg, 0.61 mmol, 1.0 eq), phenylacetylene (62 mg, 66.6 µL, 0.61 mmol, 1.0 eq), $Pd(PPh_3)_4$ (21 mg, 0.02 mmol, 0.03 eq.), $CuSO_4*5H_2O$ (2.2 mg, 0.01 mmol, 0.01 eq.) and Na ascorbate (7.8 mg, 0.04 mmol, 0.06 eq.) were weighted and put into the vial. The vial was sealed and put into the microwave at 120°C for 10 min. The TEA upper phase was removed using a pipette leaving a black DMSO phase. DCM was added to the remaining solution and Tris(2-aminoethyl)amine was added to complex the copper in solution (0.1 mL, 48.8 mg, 0.33 mmol). The DCM phase was washed with water (2x250mL) and each water phase washed with DCM (2x50mL). The combined organic phases were dried over Na_2SO_4, filtered, and evaporated. Column on silica was prepared (eluent: 2% MeOH in DCM). 8,8'-(4-(phenylethynyl)pyridine-2,6-diyl)diquinoline (**dqp**-≡-**Ph**) was isolated as a beige powder (140 mg, 0.32 mmol, 53%). ^1H NMR ($CDCl_3$, 400 MHz): δ (ppm): 7.37 (m, 3H); 7.49 (q, 2H); 7.59 (m, 2H); 7.68 (t, 2H); 7.91 (d, 2H); 8.28 (m, 6H); 9.06 (d, 2H). ESI-MS (CH_3CN) m/z: [**dqp**-≡-**Ph**+H]$^+$ calc: 434.2, found: 434.2.

Synthesis of 4-(2,6-di(quinolin-8-yl)pyridin-4-yl)-N,N-dimethylaniline (dqp-DMA).

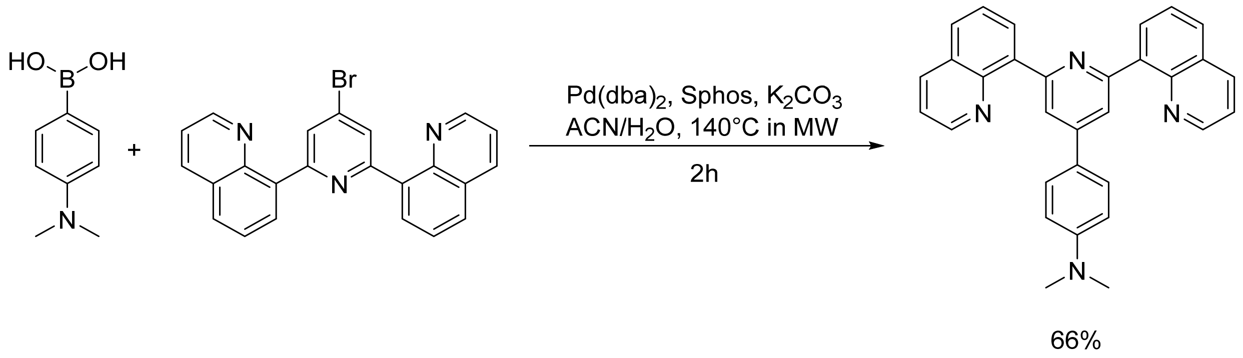

8,8'-(4-bromopyridine-2,6-diyl)diquinoline (200 mg, 0.49 mmol, 1.0 eq.), N,N-dimethylaniline boronic acid (90.1 mg, 0.55 mmol, 1.1 eq.), [$Pd_2(dba)_3$] (dba = dibenzylideneacetone) (20.7 mg, 0.04 mmol, 0.07 eq.), 2-dicyclohexylphosphino-2',6'-dimethoxybiphenyl (SPhos) (29 mg, 0.07 mmol, 0.15 eq.), K_2CO_3 (258 mg, 1.87 mmol, 3.84 eq.) in a mixture of acetonitrile:water (10 mL 3:1) were loaded into a microwave vial. The solution was bubbled with N_2 for about 10 min before being put into the MW for 2h at 140°C. After the reaction, the vial was left to cool down at RT. The ACN phase was yellow/orange and the aqueous phase colourless. The mixture was put into a separatory funnel and water and EtOAc were added (50 mL). The organic phase was extracted with water (3 x 100 mL). The organic fraction was dried over Na_2SO_4 and evaporated to dryness. Cold isopropanol (5 mL) was used to solubilise the impurity and filter-out 4-(2,6-di(quinolin-8-yl)pyridin-4-yl)-N,N-dimethylaniline (**dqp-DMA**) (145 mg, 0.32 mmol, 66%). ^1H NMR ($CDCl_3$, 400 MHz): δ (ppm): 3.03 (s, 6H); 6.82 (d, 2H); 7.47 (q, 2H); 7.69 (t, 2H); 7.78 (d, 2H); 7.90 (d, 2H); 8.29 (m, 6H); 8.99 (dd, 2H). ESI-MS (CH_3CN) m/z: [**dqp-DMA**+H]$^+$ calc: 453.2, found: 453.2.

Synthesis of 4-((2,6-di(quinolin-8-yl)pyridin-4-yl)ethynyl)-N,N-dimethylaniline (dqp-≡-DMA).

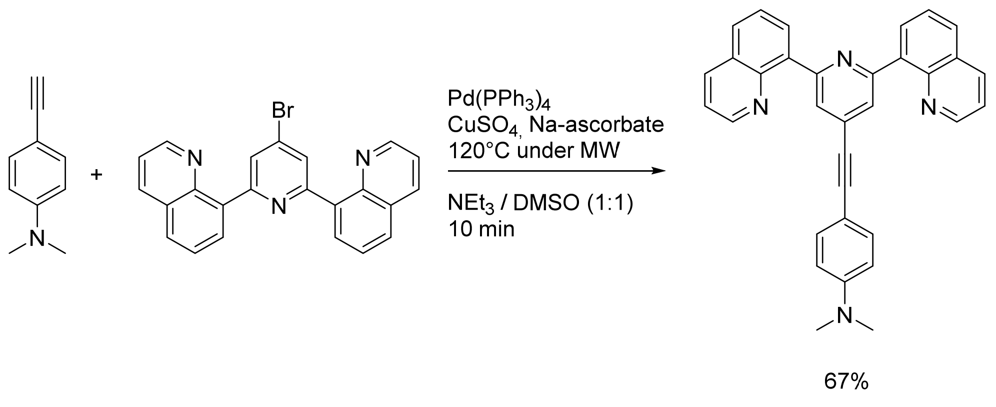

A mixture of NEt_3 and DMSO (1:1, 20mL total) was put in the microwave vial and was bubbled with N_2 for 5-10 min. 8,8'-(4-bromopyridine-2,6-diyl)diquinoline (100.7 mg, 0.24 mmol, 1.0 eq), 4-ethynyl-N,N-dimethylaniline (36.8 mg, 0.25 mmol, 1.04 eq), $Pd(PPh_3)_4$ (8.9 mg, 0.008 mmol, 0.03 eq.), $CuSO_4$*$5H_2O$ (0.6 mg, 0.002 mmol, 0.01 eq.) and Na ascorbate (5.1 mg, 0.026 mmol, 0.11 eq.) were weighted and put into the vial. The vial was sealed and put into the microwave at 120°C for 10 min. DCM was used to transfer in a flask and the DCM and TEA were evaporated using the schlenk line. DCM was added to the solution and Tris(2-aminoethyl)amine was added to complex the copper in solution (0.1 mL, 48.8 mg, 0.33 mmol). The DCM phase was washed with water (2x750mL) and each water phase washed with DCM (2x50mL). The combined organic phases were dried over Na_2SO_4, filtered, and evaporated. Column on silica was prepared (eluent: 30% AcOEt, 40% Cyclohexane, 30% DCM). 4-((2,6-di(quinolin-8-yl)pyridin-4-yl)ethynyl)-N,N-dimethylaniline (**dqp**-≡-**DMA**) was isolated as a bright orange powder (78 mg, 0.16 mmol, 67%). ^1H NMR ($CDCl_3$, 400 MHz): δ (ppm): 3.00 (s, 6H); 6.66 (dt, 2H); 7.46 (m, 4H); 7.67 (t, 2H); 7.89 (dd, 2H); 8.18 (s, 2H); 8.25 (m, 4H); 9.04 (dd, 2H). ESI-MS (CH_3CN) m/z: [**dqp**-≡-**DMA**+H]$^+$ calc: 477.2, found: 477.4.

Synthesis of 4-(2,6-di(quinolin-8-yl)pyridine (dqp).

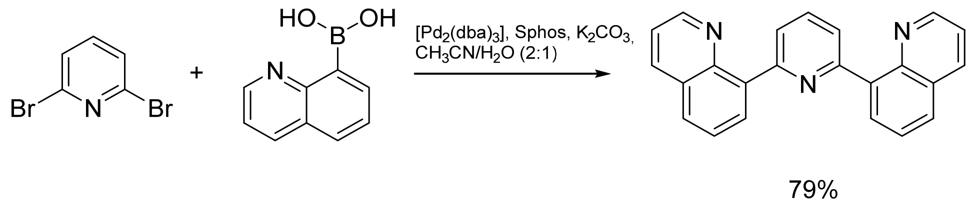

2,6-dibromopyridine (731.5 mg, 3.13 mmol, 1 eq), quinoline-8-boronic acid (1178.1 mg, 6.81 mmol, 2.2 eq), [Pd(dba)$_2$] (122.8 mg, 0.21 mmol, 7 mol%), Sphos (171.2 mg, 0.417 mmol, 13.3 mol%) and Potassium carbonate (2048 mg, 14.82 mmol, 4.74 eq) were weighed and split into two different 20 mL MW vial. 10 mL ACN and 5 mL H_2O were added. The solutions were each put into the MW for 2h at 140°C. The resulting combined mixture were extracted with water (3x250 mL) and EtOAc. The organic fraction was dried over Na_2SO_4 and evaporated to dryness. Cold EtOAc was used to solubilise the impurity. Filtration led to the isolation of 2,6-di(quinoline-8-yl)pyridine (**dqp**: 819 mg, 2.46 mmol, 79%) as an off-white powder.

^{1}H NMR ($CDCl_3$, 400 MHz): δ (ppm); 7.44 (q, 2H); 7.66 (dd, 2H); 7.88 (dd, 2H); 7.94 (dd, 2H); 8.11 (s, 2H); 8.13 (s, 2H); 8.23 (dd, 2H), 8.27 (dd, 2H); 9.00 (dd, 2H).

ESI-MS (CH_3CN) m/z: [**dqp**+H]$^+$ calc: 334.1, found: 334.1. Elemental analysis: Calcd for $C_{23}H_{15}N_3$: C, 82.86; H, 4.54; N, 12.60. Found C, 82.58; H, 4.50; N, 12.55.

Synthesis of N[2],N[6]-dimethyl-N[2],N[6]-di(pyridin-2-yl)pyridine-2,6-diamine (ddpd):

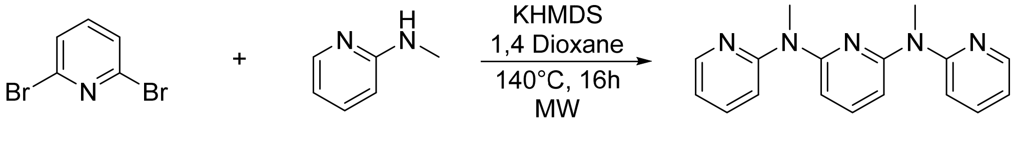

39%

Potassium bis(trimethylsilyl)amide (3.5g, 17.54 mmol, 5.20 equiv) was loaded in a MW vial and *N*-methylpyridin-2-ylamine (850 µL, 8.44 mmol, 2.5 equiv) was added. The solids were suspended in dioxane (5 mL) previously dried on molecular sieves. The solution turned yellow. 2,6-dibromopyridine (799.7 g, 3.38 mmol, 1.0 equiv) was added, and the mixture was sealed and heated at 140°C for 16 h, resulting in an orange solution. After cooling to room temperature, water (250 mL) was added, and the aqueous phase was extracted three times with tetrahydrofuran/Et_2O (1:1, 3x200 mL). The combined organic phases were washed twice times with a 1 M aqueous Na_2CO_3 solution (200 mL) and finally dried over $MgSO_4$. After removal of the solvent under reduced pressure, the product was purified by column chromatography (silica gel, cyclohexane/ethyl acetate 6/4), yielding N[2],N[6]-dimethyl-N[2],N[6]-di(pyridin-2-yl)pyridine-2,6-diamine (**ddpd**) as a yellow viscous liquid (386 mg, 1.32 mmol, 39%). ^{1}H NMR ($CDCl_3$, 400 MHz): δ (ppm); 3.61 (s, 6H); 6.72 (d, 2H); 6.87 (t, 2H); 7.26 (m, 2H); 7.44 (t, 1H); 7.53 (t, 2H); 8.36 (d, 2H); ESI-MS (CH_3CN) m/z: [**ddpd**+H]$^+$ calc: 292.3, found: 292.3.

Synthesis of the complexes

The heteroleptic complexes [Cr(dqp)(dqp-OMe)]$(SO_3CF_3)_3$ (**5**) and [Cr(dqp)(ddpd)]$(SO_3CF_3)_3$ (**6**) were synthesised according to published literature.[A1-9]

Synthesis of $Cr(CF_3SO_3)_2\cdot 2H_2O$.

$$Cr^0 + 2\,HSO_3CF_3 \xrightarrow{60°C,\ 24h,\ N_2,\ H_2O} Cr(SO_3CF_3)_2\cdot 2H_2O$$

A solution of 80 mmol of trifluoromethanesulfonic acid (7 cm^3) in degassed water (50 cm^3) at 0 °C was added via a cannula onto electrolytic chromium (2 g, 38 mmol) under nitrogen. After stirring for 24 h at 60 °C, the blue solution was filtered and evaporated to dryness. The resulting blue powder was dried under vacuum (10^{-2}Torr/48 h) and transferred into a glove box. Spectrophotometric analysis of the chromium content after oxidation into chromate (fusion with KNO_3) gives %Cr = 13.41 corresponding to $[Cr(CF_3SO_3)_2]\cdot 2H_2O$ (%Cr = 13.47). Elemental analysis: Calcd for $[Cr(CF_3SO_3)_2]$: C, 6.22; H, 1.04; N, 0.00. Found C, 6.79; H, 0.64; N, 0.00.

Synthesis of homoleptic complexes *rac*-[Cr(dqp-Ph)$_2$](SO$_3$CF$_3$)$_3$ (1), *rac*-[Cr(dqp-≡-Ph)$_2$](SO$_3$CF$_3$)$_3$ (2), *rac*-[Cr(dqp-DMA)$_2$](SO$_3$CF$_3$)$_3$ (3), *rac*-[Cr(dqp-DMAH)$_2$](SO$_3$CF$_3$)$_5$ (H$_2$-3), *rac*-[Cr(dqp-≡-DMA)$_2$](SO$_3$CF$_3$)$_3$ (4) and *rac*-[Cr(dqp-≡-DMA)$_2$](SO$_3$CF$_3$)$_5$ (H$_2$-4):

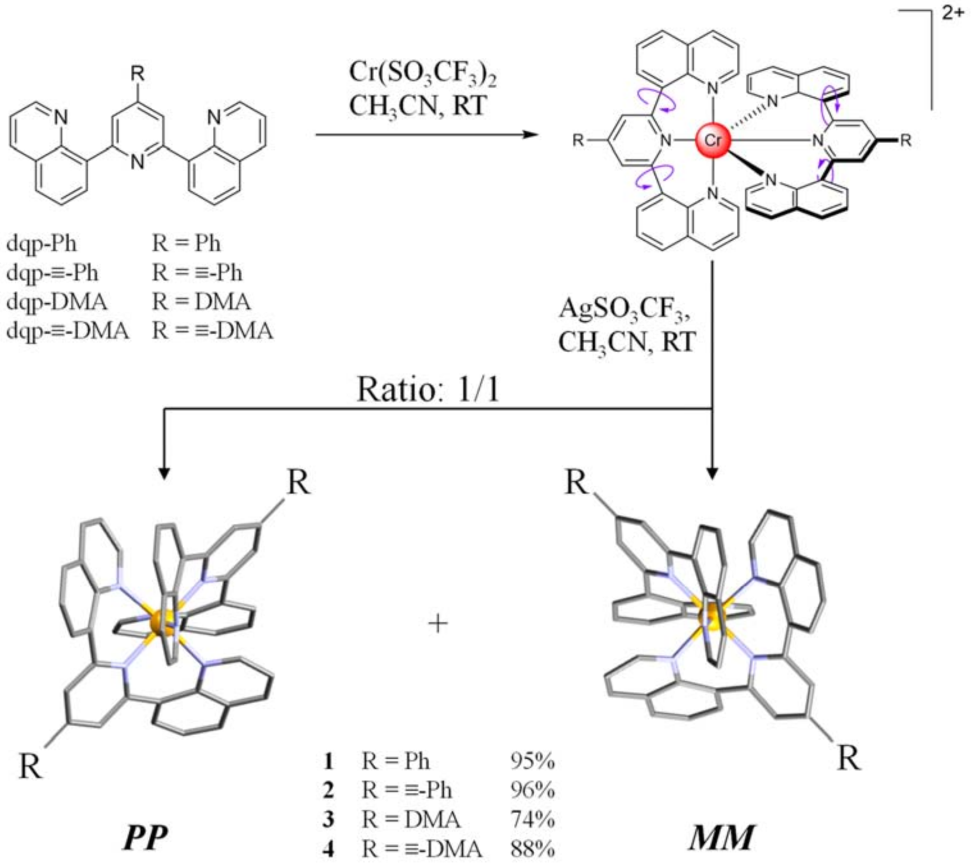

In a glovebox, Cr(CF$_3$SO$_3$)$_2$·2H$_2$O (50 mg, 0.13 mmol, 1 eq.) was charged into a Schlenk tube and degassed acetonitrile (4 mL) was added to dissolve the powder. The obtained blue-sky solution was added into a previously prepared suspension containing 2 equivalents of ligand dqp-R (R = Ph, ≡-Ph, DMA, ≡-DMA) in degassed acetonitrile (10 mL). The colour of the mixture turned to deep and intense red. After being stirred at RT for 2 h, AgSO$_3$CF$_3$ (33.3 mg, 0.13 mmol, 1.0 eq) was weighted and added to the solution. Ag(0) was formed and precipitated, and the solution became orange due to the formation of Cr(III). The vial was left under stirring for 4 more hours before being taken out of the glovebox. The solution was filtered to remove Ag(0). The solution was left open at air until dryness. The solid was re-dissolved in the smallest amount of ACN and Et$_2$O was added generously until full precipitation of the complex, that was isolated by centrifugation and dried overnight at 50°C. The orange solids of **1** and **3** were redissolved in methanol and through slow diethyl ether diffusion, crystals suitable for single

crystal diffraction analysis were obtained. The same procedure was applied to **2** with ^{t}BuOMe through slow diffusion. Anion exchange with (n-Bu_4)Cl was carried out in acetone. Slow diffusion of diethyl ether yielded crystals suitable for single crystal diffraction analysis. Additionally, the protonated derivatives of the *N*,*N*-dimethylaniline complexes H_2-**3** and H_2-**4** were successfully isolated by crystallisation upon full protonation using triflic acid in a methanolic solution and slow diffusion of diethyl ether. TOF-MS (CH_3CN) *m/z*: [[Cr(dqp-Ph)$_2$](SO_3CF_3)$_2$]$^+$ calc: 1168.159, found: 1168.099. [[Cr(dqp-≡-Ph)$_2$](SO_3CF_3)]$^+$ calc: 1216.159, found: 1216.121. [[Cr(dqp-DMA)$_2$](SO_3CF_3)]$^+$ calc: 1254.244, found: 1254.208. [[Cr(dqp-≡-DMA)$_2$](SO_3CF_3)]$^+$ calc: 1303.247, found: 1303.204. Elemental analysis: *rac*-[Cr(dqp-Ph)$_2$](SO_3CF_3)$_3$·3H_2O (**1**) Calcd for $C_{61}H_{38}CrF_9N_6O_9S_3$·3$H_2O$: C, 53.39; H, 3.23; N, 6.12. Found C, 53.01; H, 2.89; N, 6.03; MW: 1318 g/mol; *rac*-[Cr(dqp-≡-Ph)$_2$](SO_3CF_3)$_3$·2H_2O (**2**) Calcd for $C_{65}H_{38}CrF_9N_6O_9S_3$·2$H_2O$: C, 55.68; H, 3.02; N, 5.99. Found C, 55.81; H, 2.90; N, 5.91; MW: 1366 g/mol; *rac*-[Cr(dqp-DMA)$_2$](SO_3CF_3)$_3$·4.2H_2O (**3**) Calcd for $C_{65}H_{48}CrF_9N_8O_9S_3$·4.2$H_2O$: C, 52.75; H, 3.84; N, 7.57. Found C, 52.62; H, 3.77; N, 7.47; MW: 1403 g/mol; *rac*-[Cr(dqp-≡-DMA)$_2$](SO_3CF_3)$_3$·14.1 NaCl (**4**) Calcd for $C_{69}H_{48}CrF_9N_8O_9S_3$·14.1 NaCl: C, 40.96; H, 2.50; N, 5.79. Found C, 40.93; H, 3.08; N, 5.70; MW: 1413.77 g/mol; *rac*-[Cr(dqp-DMAH)$_2$](SO_3CF_3)$_5$·8CH_3CN (H_2-**3**) Calcd for $C_{67}H_{50}CrF_{15}N_8O_{15}S_5$·8$CH_3CN$: C, 49.04; H, 3.67; N, 11.02. Found C, 51.91; H, 5.07; N, 8.30; MW: 1703.11 g/mol; *rac*-[Cr(dqp-≡-DMAH)$_2$](SO_3CF_3)$_5$ ·7.5H_2O (H_2-**4**) Calcd for $C_{71}H_{50}CrF_{15}N_8O_{15}S_5$ ·7.5H_2O : C, 45.18; H, 3.47; N, 5.94. Found C, 45.33; H, 3.12; N, 5.59; MW: 1752.49 g/mol.

References

(A1-1) Maeder, M.; King, P., Analysis of chemical processes, determination of the reaction mechanism and fitting of equilibrium and rate constants. In *Chemometrics in Practical Applications*, Varmuza, K., Ed. INTECH: 2012; pp 41-62.

(A1-2) Gampp, H.; Maeder, M.; Meyer, C. J.; Zuberbuehler, A. D., Calculation of equilibrium constants from multiwavelength spectroscopic data. III. Model-free analysis of spectrophotometric and ESR titrations. *Talanta* **1985**, *32*, 1133-1139.

(A1-3) Gampp, H.; Maeder, M.; Meyer, C. J.; Zuberbuehler, A. D., Calculation of equilibrium constants from multiwavelength spectroscopic data - IV. Model-free least-squares refinement by use of evolving factor analysis. *Talanta* **1986**, *33*, 943-951.

(A1-4) Clifford, S.; Lawrance, G. A.; Neuhold, Y.-M.; Maeder, M., Conjoint analysis of kinetic and equilibrium data for mechanistic elucidation in polynuclear complexation reactions, exemplified by metal(II) helicate complex formation. *Aust. J. Chem.* **2010**, *63*, 141-144.

(A1-5) Sheldrick, G. M., *SHELXT* – Integrated Space-Group and Crystal-Structure Determination. *Acta Cryst. A* **2015**, *64*, 3-8.

(A1-6) Sheldrick, G. M., Crystal structure refinement with SHELXL. *Acta Cryst. C* **2015**, *71*, 3-8.

(A1-7) Dolomanov, O.V.; Bourhis, L. J.; Gildea, R. J.; Howard, J. A. K.; Puschmann, H., OLEX2: A Complete Structure Solution, Refinement and Analysis Program. *J. Appl. Cryst.* **2009**, *42*, 339-341.

(A1-8) Jimenez, J. R.; Poncet, M.; Miguez-Lago, S.; Grass, S.; Lacour, J.; Besnard, C.; Cuerva, J. M.; Campana, A. G.; Piguet, C., Bright Long-Lived Circularly Polarized Luminescence in Chiral Chromium(III) Complexes, *Angew. Chem. Int. Ed.* **2021,** *60* (18), 10095-10102.

(A1-9) Jiménez, J.-R.; Poncet, M.; Doistau, B.; Besnard, C.; Piguet, C., Luminescent polypyridyl heteroleptic CrIII complexes with high quantum yields and long excited state lifetimes, *Dalton Trans.* **2020,** *49* (39), 13528-13532.

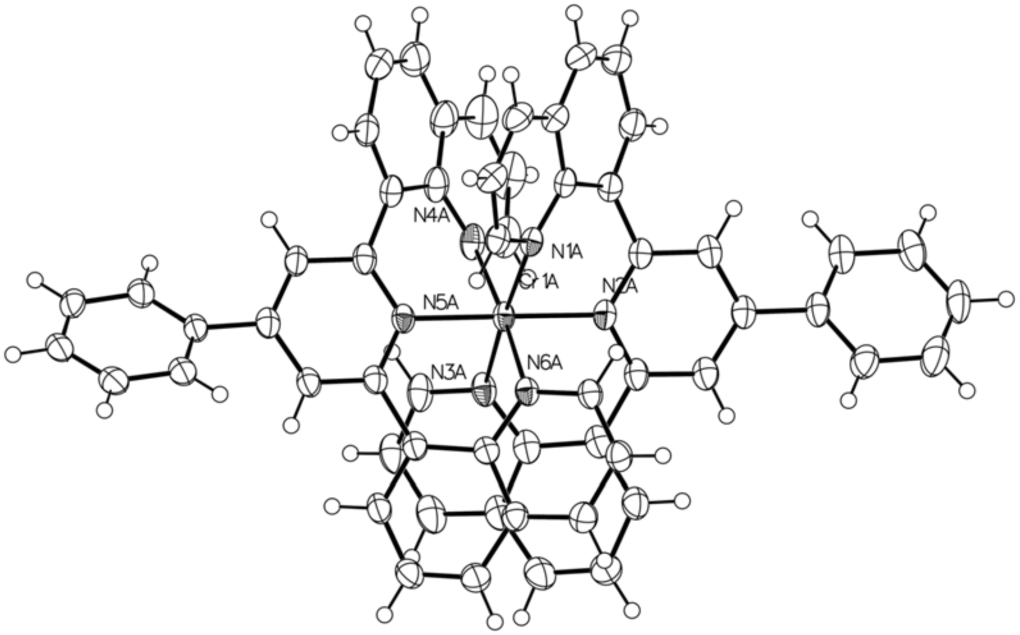

Figure S1. ORTEP view of **1** with numbering scheme. Thermal ellipsoids are drawn at 50% probability level.

Table S1. Crystal data and structure refinement for *rac*-[Cr(dqp-Ph)$_2$](SO_3CF_3)$_3$ (**1**)

CCDC	2373726	
Empirical formula	C123.64 H82.57 Cr2 F18 N12 O19.64 S6	
Formula weight	2688.76	
Temperature	120.00(10) K	
Wavelength	1.54184 Å	
Crystal system	Triclinic	
Space group	P-1	
Unit cell dimensions	a = 15.26011(17) Å	α = 96.7520(7)°
	b = 19.33962(19) Å	β = 97.5214(7)°
	c = 21.24758(13) Å	γ = 95.9234(8)°
Volume	6128.67(10) Å^3	
Z	2	
Density (calculated)	1.457 Mg/m^3	
Absorption coefficient	3.283 mm^{-1}	
F(000)	2743	
Crystal size	0.134 x 0.069 x 0.02 mm^3	
Theta range for data collection	2.317 to 76.387°.	

Index ranges	-11<=h<=18, -24<=k<=24, -26<=l<=26
Reflections collected	143464
Independent reflections	24814 [R(int) = 0.0537]
Completeness to theta = 67.684°	99.7 %
Absorption correction	Gaussian
Max. and min. transmission	1.000 and 0.784
Refinement method	Full-matrix least-squares on F^2
Data / restraints / parameters	24814 / 1441 / 1916
Goodness-of-fit on F^2	1.027
Final R indices [I>2sigma(I)]	R1 = 0.0715, wR2 = 0.1978
R indices (all data)	R1 = 0.0770, wR2 = 0.2019
Extinction coefficient	n/a
Largest diff. peak and hole	2.340 and -1.090 e.Å^{-3}

Table S2. Selected bond distances (Å), bond angles (°) in *rac*-[Cr(dqp-Ph)$_2$](SO$_3$CF$_3$)$_3$ (**1**).

Cr(1A)-N(6A)	2.060(3)
Cr(1A)-N(2A)	2.046(3)
Cr(1A)-N(1A)	2.070(3)
Cr(1A)-N(4A)	2.058(3)
Cr(1A)-N(5A)	2.037(3)
Cr(1A)-N(3A)	2.057(3)
Cr(1B)-N(3B)	2.060(3)
Cr(1B)-N(2B)	2.041(3)
Cr(1B)-N(5B)	2.041(3)
Cr(1B)-N(1B)	2.060(3)
Cr(1B)-N(6B)	2.067(3)
Cr(1B)-N(4B)	2.064(3)
N(6A)-Cr(1A)-N(1A)	88.06(10)
N(2A)-Cr(1A)-N(6A)	91.69(10)
N(2A)-Cr(1A)-N(1A)	88.18(10)
N(2A)-Cr(1A)-N(4A)	92.78(11)
N(2A)-Cr(1A)-N(3A)	87.33(11)
N(4A)-Cr(1A)-N(6A)	175.53(10)

N(4A)-Cr(1A)-N(1A)	92.12(11)
N(5A)-Cr(1A)-N(6A)	88.18(10)
N(5A)-Cr(1A)-N(2A)	179.78(12)
N(5A)-Cr(1A)-N(1A)	91.99(10)
N(5A)-Cr(1A)-N(4A)	87.35(11)
N(5A)-Cr(1A)-N(3A)	92.51(10)
N(3A)-Cr(1A)-N(6A)	92.81(11)
N(3A)-Cr(1A)-N(1A)	175.44(10)
N(3A)-Cr(1A)-N(4A)	87.36(12)
N(3B)-Cr(1B)-N(1B)	176.50(11)
N(3B)-Cr(1B)-N(6B)	94.63(10)
N(3B)-Cr(1B)-N(4B)	86.51(10)
N(2B)-Cr(1B)-N(3B)	92.03(10)
N(2B)-Cr(1B)-N(5B)	178.26(10)
N(2B)-Cr(1B)-N(1B)	91.05(11)
N(2B)-Cr(1B)-N(6B)	88.69(10)
N(2B)-Cr(1B)-N(4B)	88.48(10)
N(5B)-Cr(1B)-N(3B)	88.25(10)
N(5B)-Cr(1B)-N(1B)	88.72(11)
N(5B)-Cr(1B)-N(6B)	89.58(10)
N(5B)-Cr(1B)-N(4B)	93.25(10)
N(1B)-Cr(1B)-N(6B)	87.09(10)
N(1B)-Cr(1B)-N(4B)	91.92(10)
N(4B)-Cr(1B)-N(6B)	176.98(11)

Symmetry transformations used to generate equivalent atoms:

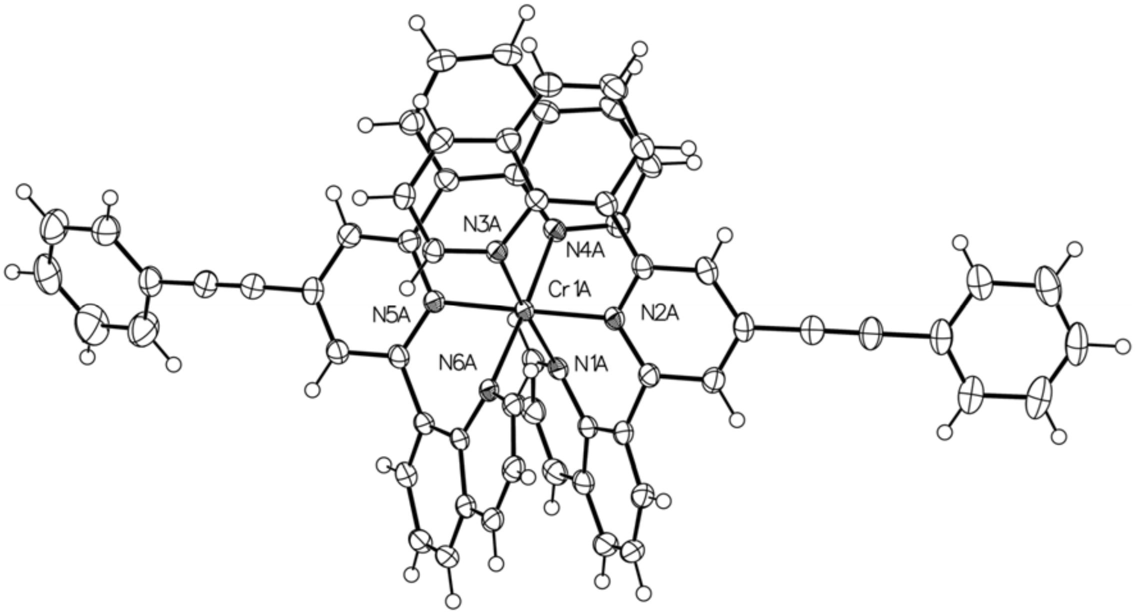

Figure S2. ORTEP view of **2** with numbering scheme. Thermal ellipsoids are drawn at 50% probability level.

Table S3. Crystal data and structure refinement for *rac*-[Cr(dqp-≡-Ph)$_2$](SO_3CF_3)$_3$ (**2**).

CCDC	2373727	
Empirical formula	C131.57 H82.29 Cr2 F18 N12 O19.57 S6	
Formula weight	2782.69	
Temperature	119.99(10) K	
Wavelength	1.54184 Å	
Crystal system	Triclinic	
Space group	P-1	
Unit cell dimensions	a = 11.27764(6) Å	α = 83.0057(5)°
	b = 14.86717(8) Å	β = 85.0334(5)°
	c = 39.6399(2) Å	γ = 79.6761(4)°
Volume	6475.75(6) Å^3	
Z	2	
Density (calculated)	1.427 Mg/m^3	
Absorption coefficient	3.128 mm^{-1}	
F(000)	2837	
Crystal size	0.64 x 0.085 x 0.045 mm^3	
Theta range for data collection	2.251 to 76.197°.	
Index ranges	-13<=h<=8, -18<=k<=18, -49<=l<=49	
Reflections collected	164648	

Independent reflections	26293 [R(int) = 0.0310]
Completeness to theta = 67.684°	99.8 %
Absorption correction	Gaussian
Max. and min. transmission	1.000 and 0.482
Refinement method	Full-matrix least-squares on F^2
Data / restraints / parameters	26293 / 425 / 1799
Goodness-of-fit on F^2	1.046
Final R indices [I>2sigma(I)]	R1 = 0.0531, wR2 = 0.1482
R indices (all data)	R1 = 0.0584, wR2 = 0.1517
Extinction coefficient	n/a
Largest diff. peak and hole	1.423 and -0.933 e.$Å^{-3}$

Table S4. Selected bond distances (Å), bond angles (°) in *rac*-[Cr(dqp-≡-Ph)$_2$](SO_3CF_3)$_3$ (**2**).

Cr(1A)-N(6A)	2.0627(19)
Cr(1A)-N(1A)	2.075(2)
Cr(1A)-N(3A)	2.052(2)
Cr(1A)-N(5A)	2.0496(19)
Cr(1A)-N(4A)	2.066(2)
Cr(1A)-N(2A)	2.031(2)
Cr(1B)-N(5B)	2.032(2)
Cr(1B)-N(2B)	2.040(2)
Cr(1B)-N(6B)	2.064(2)
Cr(1B)-N(4B)	2.062(2)
Cr(1B)-N(1B)	2.058(2)
Cr(1B)-N(3B)	2.062(2)
N(6A)-Cr(1A)-N(1A)	92.69(8)
N(6A)-Cr(1A)-N(4A)	176.71(8)
N(3A)-Cr(1A)-N(6A)	88.68(8)
N(3A)-Cr(1A)-N(1A)	176.71(8)
N(3A)-Cr(1A)-N(4A)	91.57(8)
N(5A)-Cr(1A)-N(6A)	88.66(8)
N(5A)-Cr(1A)-N(1A)	91.01(8)
N(5A)-Cr(1A)-N(3A)	92.02(8)
N(5A)-Cr(1A)-N(4A)	88.05(8)
N(4A)-Cr(1A)-N(1A)	87.23(8)

N(2A)-Cr(1A)-N(6A)	91.98(8)
N(2A)-Cr(1A)-N(1A)	88.92(8)
N(2A)-Cr(1A)-N(3A)	88.04(8)
N(2A)-Cr(1A)-N(5A)	179.35(8)
N(2A)-Cr(1A)-N(4A)	91.30(8)
N(5B)-Cr(1B)-N(2B)	179.32(8)
N(5B)-Cr(1B)-N(6B)	91.27(8)
N(5B)-Cr(1B)-N(4B)	91.64(8)
N(5B)-Cr(1B)-N(1B)	88.76(8)
N(5B)-Cr(1B)-N(3B)	88.17(8)
N(2B)-Cr(1B)-N(6B)	88.41(9)
N(2B)-Cr(1B)-N(4B)	88.69(8)
N(2B)-Cr(1B)-N(1B)	91.86(8)
N(2B)-Cr(1B)-N(3B)	91.22(8)
N(4B)-Cr(1B)-N(6B)	177.06(8)
N(1B)-Cr(1B)-N(6B)	92.85(8)
N(1B)-Cr(1B)-N(4B)	86.77(8)
N(1B)-Cr(1B)-N(3B)	176.92(9)
N(3B)-Cr(1B)-N(6B)	87.06(8)
N(3B)-Cr(1B)-N(4B)	93.48(8)

Symmetry transformations used to generate equivalent atoms:

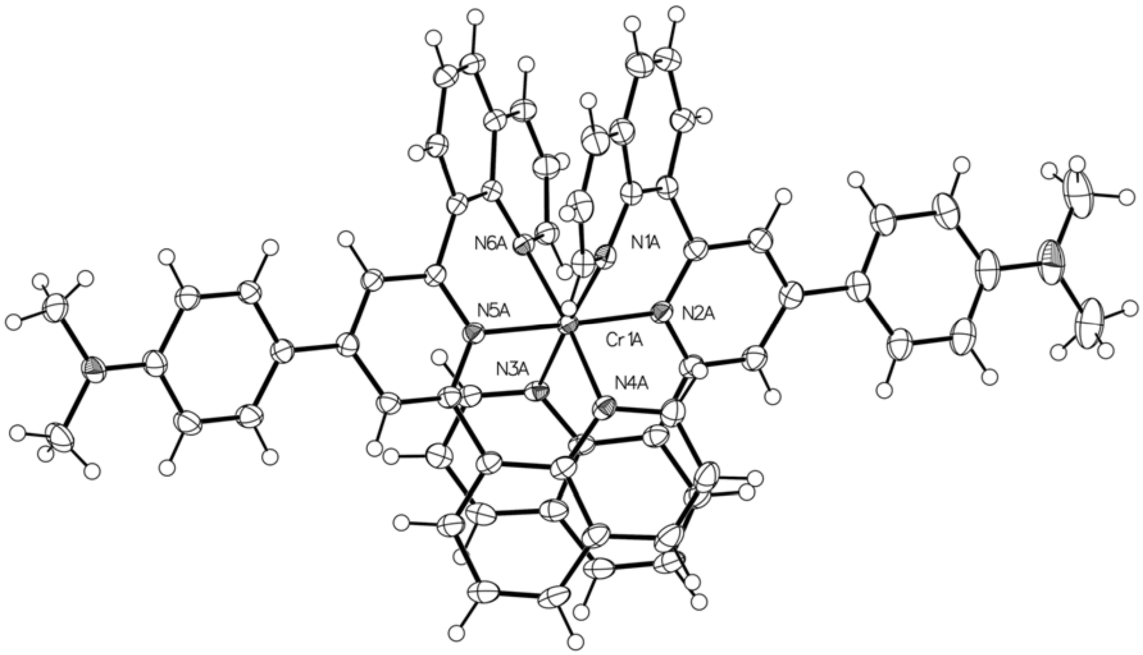

Figure S4. ORTEP view of **3** with numbering scheme. Thermal ellipsoids are drawn at 50% probability level.

Table S5. Crystal data and structure refinement for *rac*-[Cr(dqp-DMA)$_2$](SO_3CF_3)$_3$ (**3**).

CCDC	2373728	
Empirical formula	C68.91 H59.22 Cr F9 N8 O10.70 S3	
Formula weight	1489.80	
Temperature	120.00(10) K	
Wavelength	1.54184 Å	
Crystal system	Triclinic	
Space group	P-1	
Unit cell dimensions	a = 15.91222(12) Å	α = 85.7780(5)°
	b = 16.65807(11) Å	β = 85.5442(6)°
	c = 28.60662(19) Å	γ = 62.6924(7)°
Volume	6711.09(9) Å^3	
Z	4	
Density (calculated)	1.475 Mg/m^3	
Absorption coefficient	3.073 mm^{-1}	
F(000)	3069	
Crystal size	0.879 x 0.247 x 0.109 mm^3	
Theta range for data collection	3.102 to 76.310°.	
Index ranges	-19<=h<=12, -20<=k<=20, -35<=l<=35	

Reflections collected	179662
Independent reflections	27283 [R(int) = 0.0287]
Completeness to theta = 67.684°	99.9 %
Absorption correction	Gaussian
Max. and min. transmission	1.000 and 0.079
Refinement method	Full-matrix least-squares on F^2
Data / restraints / parameters	27283 / 364 / 2048
Goodness-of-fit on F^2	1.024
Final R indices [I>2sigma(I)]	R1 = 0.0687, wR2 = 0.1895
R indices (all data)	R1 = 0.0701, wR2 = 0.1906
Extinction coefficient	0.00013(3)
Largest diff. peak and hole	1.614 and -1.186 e.$Å^{-3}$

Table S6. Selected bond distances (Å), bond angles (°) in *rac*-[Cr(dqp-DMA)$_2$](SO_3CF_3)$_3$ (**3**).

Cr(1A)-N(5A)	2.037(2)
Cr(1A)-N(2A)	2.036(2)
Cr(1A)-N(6A)	2.065(2)
Cr(1A)-N(4A)	2.066(2)
Cr(1A)-N(1A)	2.068(2)
Cr(1A)-N(3A)	2.066(2)
Cr(1B)-N(2B)	2.029(2)
Cr(1B)-N(5B)	2.027(2)
Cr(1B)-N(3B)	2.067(2)
Cr(1B)-N(4B)	2.065(3)
Cr(1B)-N(1B)	2.065(2)
Cr(1B)-N(6B)	2.058(3)
N(5A)-Cr(1A)-N(6A)	87.65(9)
N(5A)-Cr(1A)-N(4A)	88.44(9)
N(5A)-Cr(1A)-N(1A)	91.19(9)
N(5A)-Cr(1A)-N(3A)	92.60(9)
N(2A)-Cr(1A)-N(5A)	178.07(9)
N(2A)-Cr(1A)-N(6A)	90.90(9)
N(2A)-Cr(1A)-N(4A)	93.05(9)
N(2A)-Cr(1A)-N(1A)	87.62(9)
N(2A)-Cr(1A)-N(3A)	88.59(9)
N(6A)-Cr(1A)-N(4A)	175.48(10)

N(6A)-Cr(1A)-N(1A)	93.36(9)
N(6A)-Cr(1A)-N(3A)	86.57(9)
N(4A)-Cr(1A)-N(1A)	89.00(10)
N(3A)-Cr(1A)-N(4A)	91.33(9)
N(3A)-Cr(1A)-N(1A)	176.21(9)
N(2B)-Cr(1B)-N(3B)	89.08(10)
N(2B)-Cr(1B)-N(4B)	91.73(10)
N(2B)-Cr(1B)-N(1B)	88.35(10)
N(2B)-Cr(1B)-N(6B)	90.92(10)
N(5B)-Cr(1B)-N(2B)	179.42(10)
N(5B)-Cr(1B)-N(3B)	91.31(10)
N(5B)-Cr(1B)-N(4B)	88.68(10)
N(5B)-Cr(1B)-N(1B)	91.26(10)
N(5B)-Cr(1B)-N(6B)	88.68(10)
N(4B)-Cr(1B)-N(3B)	92.87(10)
N(1B)-Cr(1B)-N(3B)	177.43(10)
N(1B)-Cr(1B)-N(4B)	87.30(10)
N(6B)-Cr(1B)-N(3B)	86.36(10)
N(6B)-Cr(1B)-N(4B)	177.24(10)
N(6B)-Cr(1B)-N(1B)	93.58(10)

Symmetry transformations used to generate equivalent atoms:

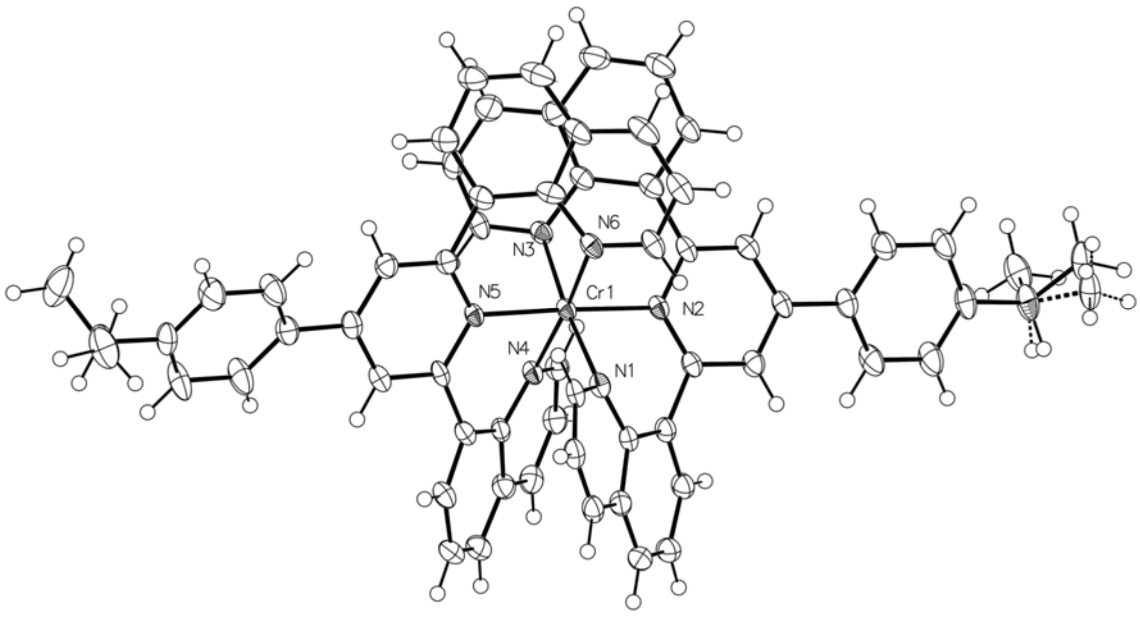

Figure S5. ORTEP view of H_2-**3** with numbering scheme. Thermal ellipsoids are drawn at 50% probability level.

Table S7. Crystal data and structure refinement for *rac*-[Cr(dqp-DMAH)$_2$](SO_3CF_3)$_5$ (H_2-**3**).

CCDC	2373729	
Empirical formula	C71 H56 Cr F15 N10 O15 S5	
Formula weight	1786.55	
Temperature	99.99(13) K	
Wavelength	1.54184 Å	
Crystal system	Monoclinic	
Space group	C 2/c	
Unit cell dimensions	a = 33.6160(4) Å	α = 90°
	b = 8.98170(10) Å	β = 106.929(2)°
	c = 53.2118(8) Å	γ = 90°
Volume	15370.0(4) Å^3	
Z	8	
Density (calculated)	1.544 Mg/m^3	
Absorption coefficient	3.452 mm^{-1}	
F(000)	7288	
Crystal size	0.423 x 0.272 x 0.049 mm^3	
Theta range for data collection	2.748 to 74.923°.	
Index ranges	-41<=h<=41, -11<=k<=9, -63<=l<=66	
Reflections collected	64520	

Independent reflections	15474 [R(int) = 0.0393]
Completeness to theta = 67.684°	99.8 %
Absorption correction	Gaussian
Max. and min. transmission	1.000 and 0.201
Refinement method	Full-matrix least-squares on F^2
Data / restraints / parameters	15474 / 301 / 1226
Goodness-of-fit on F^2	1.108
Final R indices [I>2sigma(I)]	R1 = 0.0760, wR2 = 0.1738
R indices (all data)	R1 = 0.0800, wR2 = 0.1757
Extinction coefficient	n/a
Largest diff. peak and hole	0.933 and -0.698 e.Å^{-3}

Table S8. Selected bond lengths (Å) and angles (°) for *rac*-[Cr(dqp-DMAH)$_2$](SO$_3$CF$_3$)$_5$ (H$_2$-**3**).

Cr(1)-N(1)	2.058(3)
Cr(1)-N(2)	2.051(3)
Cr(1)-N(3)	2.060(3)
Cr(1)-N(4)	2.069(3)
Cr(1)-N(5)	2.047(3)
Cr(1)-N(6)	2.070(3)
N(1)-Cr(1)-N(3)	174.75(12)
N(1)-Cr(1)-N(4)	93.50(12)
N(1)-Cr(1)-N(6)	87.53(13)
N(2)-Cr(1)-N(1)	87.07(12)
N(2)-Cr(1)-N(3)	87.79(12)
N(2)-Cr(1)-N(4)	91.16(12)
N(2)-Cr(1)-N(6)	93.65(12)
N(3)-Cr(1)-N(4)	87.64(12)
N(3)-Cr(1)-N(6)	91.76(13)
N(4)-Cr(1)-N(6)	175.12(12)
N(5)-Cr(1)-N(1)	91.44(12)
N(5)-Cr(1)-N(2)	178.05(12)
N(5)-Cr(1)-N(3)	93.72(12)
N(5)-Cr(1)-N(4)	87.66(12)
N(5)-Cr(1)-N(6)	87.55(12)

Symmetry transformations used to generate equivalent atoms:

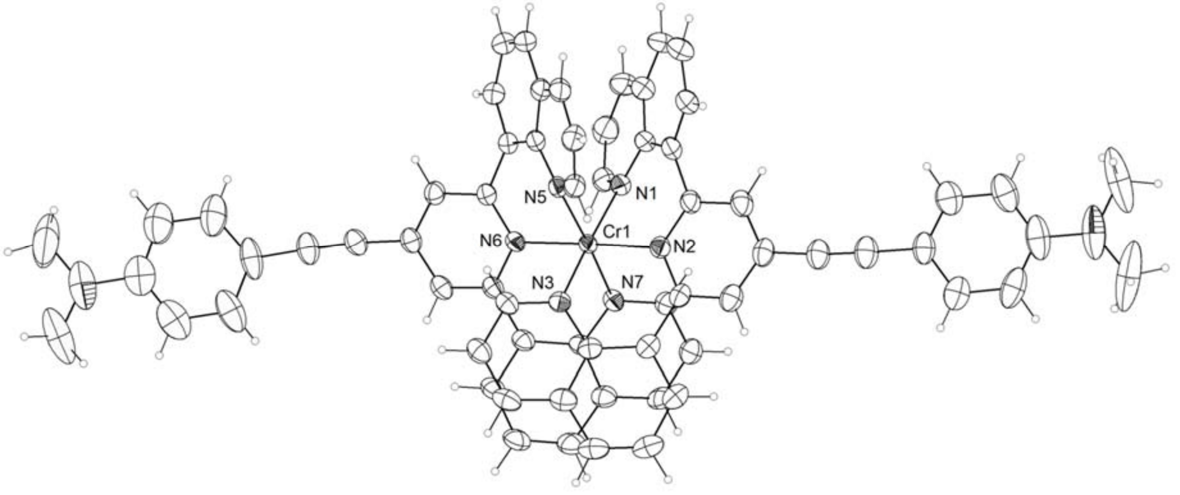

Figure S6. ORTEP view of **4** with numbering scheme. Thermal ellipsoids are drawn at 50% probability level.

Table S9. Crystal data and structure refinement for *rac*-[Cr(dqp-≡-DMA)$_2$](Cl)$_3$ (**4**).

CCDC	2373730	
Empirical formula	C68 H56 Cl3 Cr N8 O2	
Formula weight	1175.55	
Temperature	100.00(10) K	
Wavelength	1.54184 Å	
Crystal system	Triclinic	
Space group	P -1	
Unit cell dimensions	a = 14.05053(16) Å	α= 71.1168(8)°.
	b = 14.19137(16) Å	β= 73.8126(8)°.
	c = 19.59955(15) Å	γ = 83.8344(9)°.
Volume	3550.52(7) Å^3	
Z	2	
Density (calculated)	1.100 Mg/m^3	
Absorption coefficient	2.713 mm^{-1}	
F(000)	1222	
Crystal size	0.251 x 0.167 x 0.15 mm^3	
Theta range for data collection	2.467 to 75.895°.	
Index ranges	-17<=h<=17, -17<=k<=17, -24<=l<=19	

Reflections collected	62845
Independent reflections	14366 [R(int) = 0.0316]
Completeness to theta = 67.684°	99.8 %
Absorption correction	Analytical
Max. and min. transmission	0.739 and 0.655
Refinement method	Full-matrix least-squares on F^2
Data / restraints / parameters	14366 / 9 / 763
Goodness-of-fit on F^2	1.081
Final R indices [I>2sigma(I)]	R1 = 0.0786, wR2 = 0.2366
R indices (all data)	R1 = 0.0859, wR2 = 0.2453
Extinction coefficient	n/a
Largest diff. peak and hole	1.953 and -1.982 e.Å^{-3}

Comment on the crystal structure:

Disorder:

Three chlorine counter ions are found in the structure but one is highly disordered on a symmetry center. It was refined in two parts with fixed occupancies 0.5. This Cl atom located on the inversion center show very large anisotropic displacement parameters due to a large disorder along this direction. Attempts to better modelized this disorder leads to unstable refinement.

Solvent methanol molecules were also found in voids of the crystal structure.
Some of them were included in the refinement, but others are too disordered to be modeled correctly. A solvent mask was calculated (as implemented in Olex2 software, an alternative to Squeeze in Platon) and 283 electrons were found in a volume of 1032 Å^3 in 1 void per unit cell. This is consistent with the presence of 3[C5H12O] per Formula Unit which account for 300 electrons per unit cell (but not included in the reported total chemical formula sum).

Table S10. Hydrogen bonds for *rac*-[Cr(dqp-≡-DMA)$_2$](Cl)$_3$ (**4**) [Å and °].

D-H...A	d(D-H)	d(H...A)	d(D...A)	<(DHA)
O(1S)-H(1S)...Cl(1)	0.84	2.30	3.107(5)	161.4
O(3S)-H(3S)...Cl(2)	0.94	2.18	3.050(5)	154.0

Table S11. Selected bond distances (Å), bond angles (°) in *rac*-[Cr(dqp-≡-DMA)$_2$](Cl)$_3$ (**4**).

Cr(1)-N(1)	2.066(2)
Cr(1)-N(2)	2.031(2)
Cr(1)-N(3)	2.057(2)
Cr(1)-N(5)	2.063(2)
Cr(1)-N(6)	2.041(2)
Cr(1)-N(7)	2.069(2)
N(1)-Cr(1)-N(7)	87.86(10)
N(2)-Cr(1)-N(1)	88.81(10)
N(2)-Cr(1)-N(3)	87.64(10)
N(2)-Cr(1)-N(5)	91.91(9)
N(2)-Cr(1)-N(6)	179.06(10)
N(2)-Cr(1)-N(7)	91.92(10)
N(3)-Cr(1)-N(1)	176.36(9)
N(3)-Cr(1)-N(5)	87.29(9)
N(3)-Cr(1)-N(7)	93.09(9)
N(5)-Cr(1)-N(1)	92.00(10)
N(5)-Cr(1)-N(7)	176.16(9)
N(6)-Cr(1)-N(1)	92.08(10)
N(6)-Cr(1)-N(3)	91.48(9)
N(6)-Cr(1)-N(5)	88.36(9)
N(6)-Cr(1)-N(7)	87.80(9)

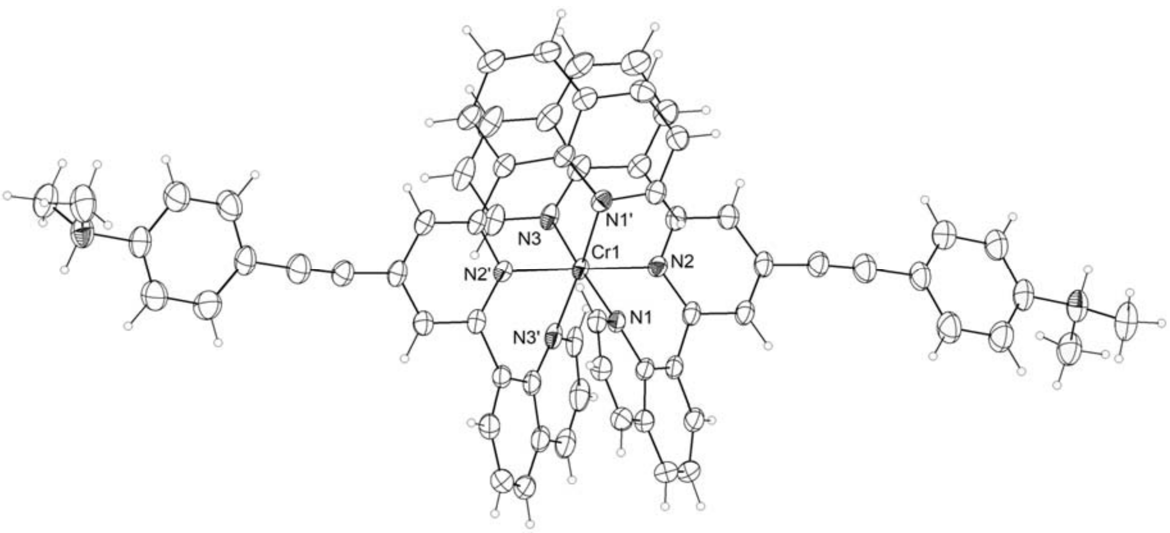

Figure S7. ORTEP view of H_2-**4** with numbering scheme. Thermal ellipsoids are drawn at 50% probability level.

Table S12. Crystal data and structure refinement for *rac*-[Cr(dqp-≡-DMAH)$_2$](SO_3CF_3)$_5$ (H_2-**4**).

CCDC	2373731	
Empirical formula	C79 H62 Cr F15 N12 O15 S5	
Chemical formula moiety	$C_{66}H_{50}CrN_8$, 5(CF_3O_3S), 4(C_2H_3N)	
Formula weight	1916.70	
Temperature	120.00(10) K	
Wavelength	1.54184 Å	
Crystal system	Orthorhombic	
Space group	P n n a	
Unit cell dimensions	a = 26.78232(19) Å	α = 90°.
	b = 38.7943(3) Å	β = 90°.
	c = 8.34436(6) Å	γ = 90°.
Volume	8669.80(11) Å^3	
Z	4	
Density (calculated)	1.468 Mg/m^3	
Absorption coefficient	3.110 mm^{-1}	
F(000)	3916	
Crystal size	0.26 x 0.19 x 0.06 mm^3	
Theta range for data collection	2.278 to 74.381°.	
Index ranges	-31<=h<=33, -48<=k<=46, -9<=l<=10	
Reflections collected	79768	

Independent reflections	8785 [R(int) = 0.0389]
Completeness to theta = 67.684°	100.0 %
Absorption correction	Analytical
Max. and min. transmission	0.878 and 0.561
Refinement method	Full-matrix least-squares on F^2
Data / restraints / parameters	8785 / 7 / 594
Goodness-of-fit on F^2	1.073
Final R indices [I>2sigma(I)]	R1 = 0.0831, wR2 = 0.2442
R indices (all data)	R1 = 0.0870, wR2 = 0.2478
Extinction coefficient	n/a
Largest diff. peak and hole	1.435 and -0.730 e.Å^{-3}

Comments on the crystal structure:

This chromium complex is located around symmetry elements (Cr on a 2-fold axis) and count for 0.5 molecule in the asymmetric unit (Z=4 and Z'=0.5). Two and half triflates and 2 acetonitrile molecules complete the structure, leading to a total chemical formula moiety: C66 H50 Cr N8, 5(C F3 O3 S), 4(C2 H3 N).

Disorder: A triflate ion is located on and disordered around a symmetry element. Its disordered CF_3 unit was refined with isotropic atomic displacement parameters.

Two acetonitrile solvent molecules were also disordered and were refined with isotropic ADPs and partial occupancies. One with occupancy fixed to 0.5 and the second refined in two parts with fixed occupancies of 0.25 each.

Table S13. Selected bond lengths (Å) and angles (°) for *rac*-[Cr(dqp-≡-DMAH)$_2$](SO_3CF_3)$_5$ (H_2-**4**).

Cr(1)-N(1)	2.070(3)
Cr(1)-N(1)#1	2.070(3)
Cr(1)-N(2)	2.042(3)
Cr(1)-N(2)#1	2.042(3)
Cr(1)-N(3)	2.062(3)
Cr(1)-N(3)#1	2.062(3)
N(1)#1-Cr(1)-N(1)	87.76(16)
N(2)#1-Cr(1)-N(1)	91.97(11)
N(2)-Cr(1)-N(1)#1	91.97(11)
N(2)-Cr(1)-N(1)	88.12(11)
N(2)#1-Cr(1)-N(1)#1	88.12(11)
N(2)-Cr(1)-N(2)#1	179.88(16)

N(2)#1-Cr(1)-N(3)#1	87.95(11)
N(2)-Cr(1)-N(3)	87.95(11)
N(2)-Cr(1)-N(3)#1	91.96(11)
N(2)#1-Cr(1)-N(3)	91.96(11)
N(3)-Cr(1)-N(1)	176.05(10)
N(3)#1-Cr(1)-N(1)#1	176.05(10)
N(3)-Cr(1)-N(1)#1	92.81(12)
N(3)#1-Cr(1)-N(1)	92.81(12)
N(3)#1-Cr(1)-N(3)	86.88(17)
C(1)-N(1)-Cr(1)	120.1(2)

Symmetry transformations used to generate equivalent atoms:
#1 -x+3/2,-y+1,z #2 x,-y+1/2,-z+1/2

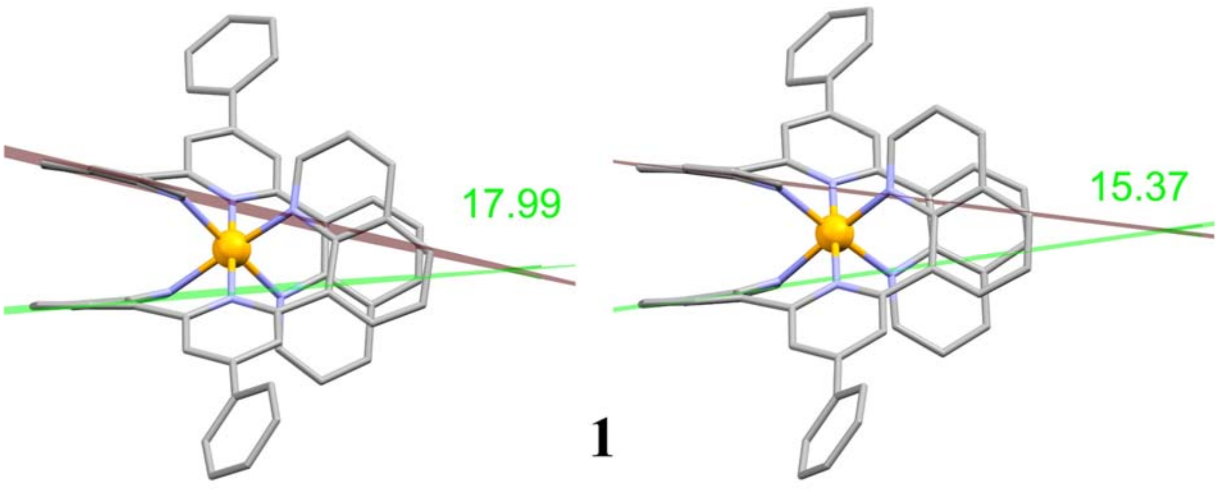

Figure S8. Interplanar angle calculated for **1**. Angles are calculated in degrees (°).

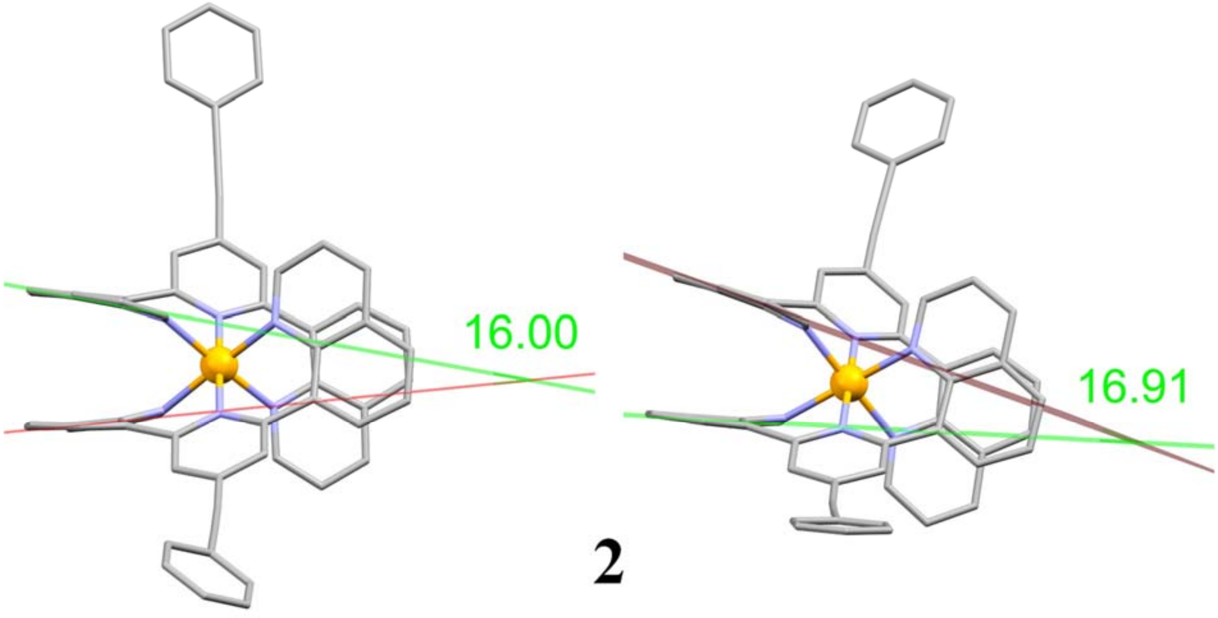

Figure S9. Interplanar angle calculated for **2**. Angles are calculated in degrees (°).

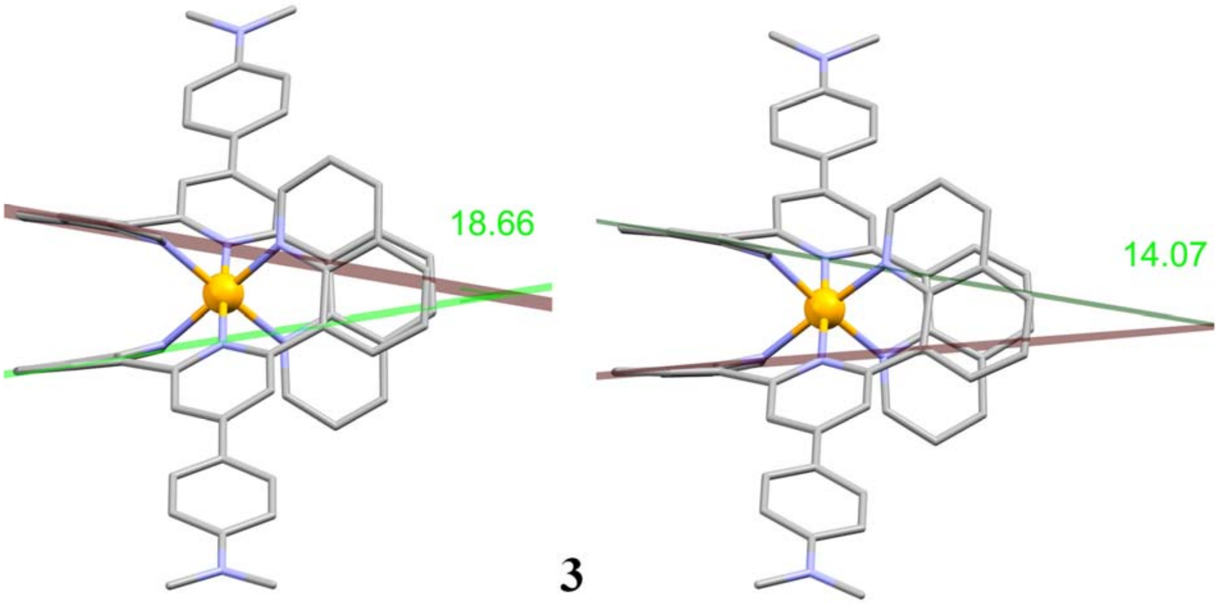

Figure S10. Interplanar angle calculated for **3**. Angles are calculated in degrees (°).

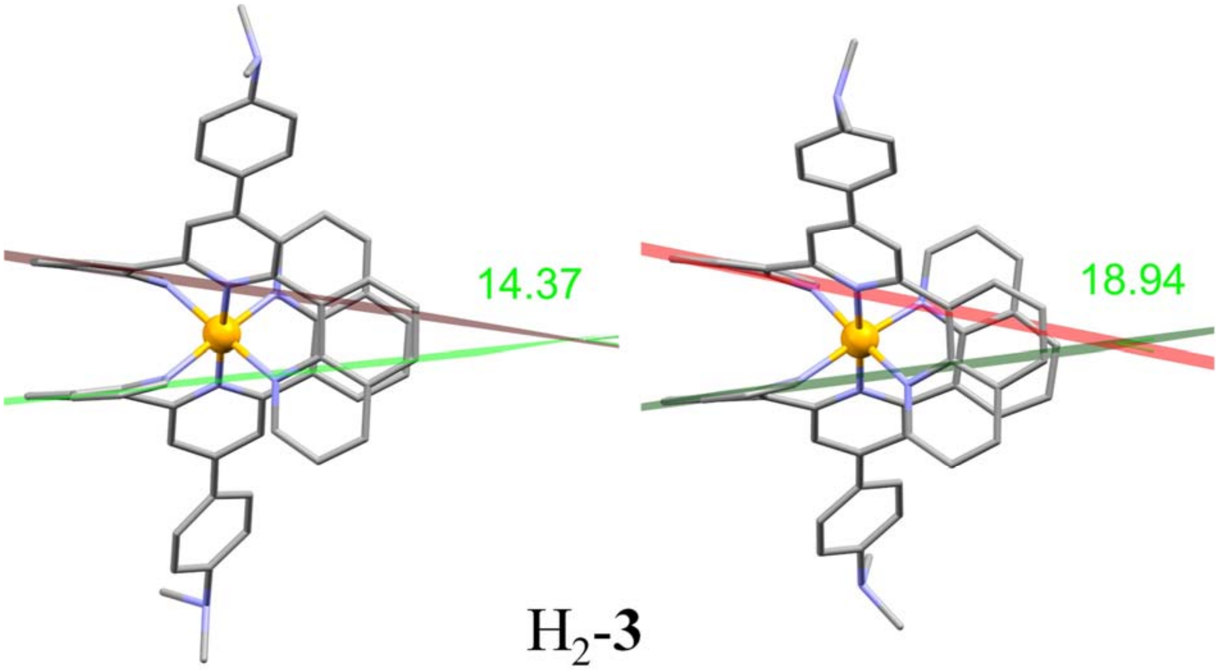

Figure S11. Interplanar angle calculated for H$_2$-**3**. Angles are calculated in degrees (°).

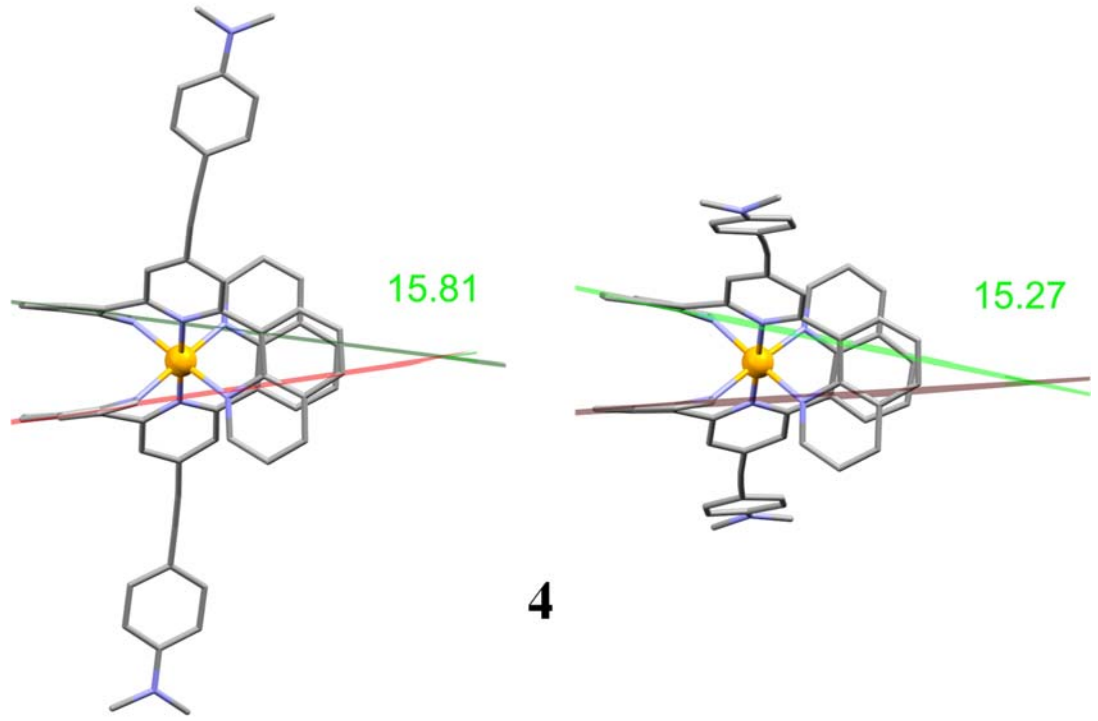

4

Figure S12. Interplanar angle calculated for **4**. Angles are calculated in degrees (°).

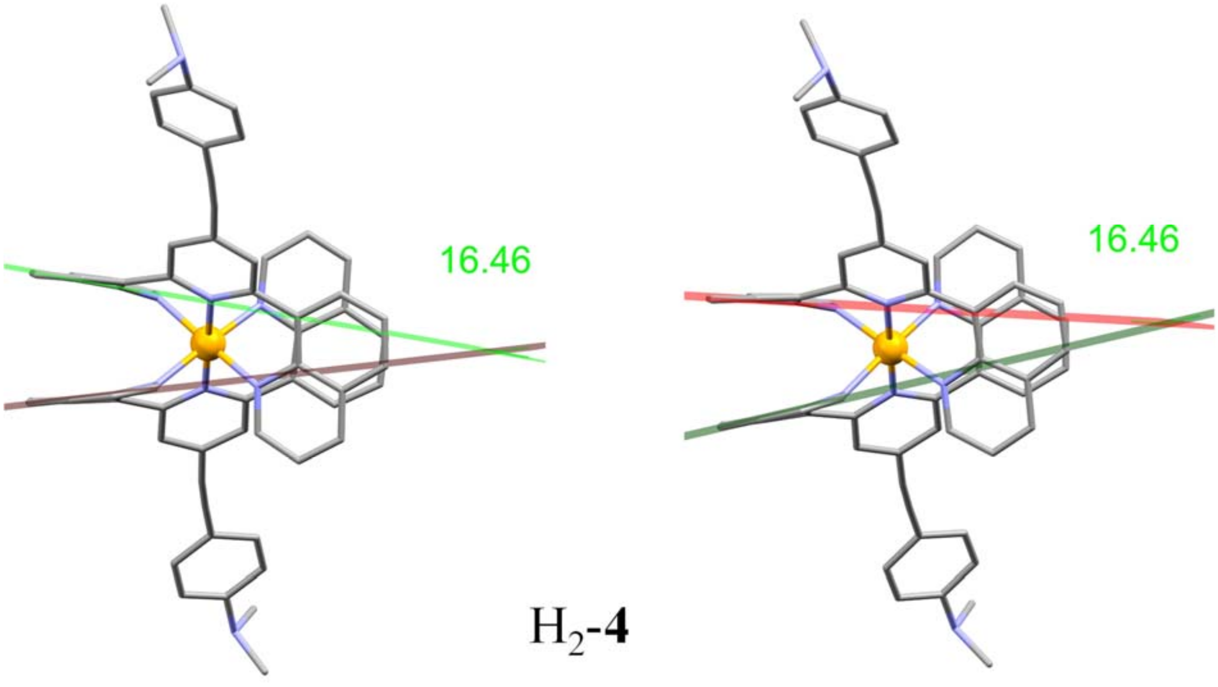

H_2-**4**

Figure S13. Interplanar angle calculated for H_2-**4**. Angles are calculated in degrees (°).

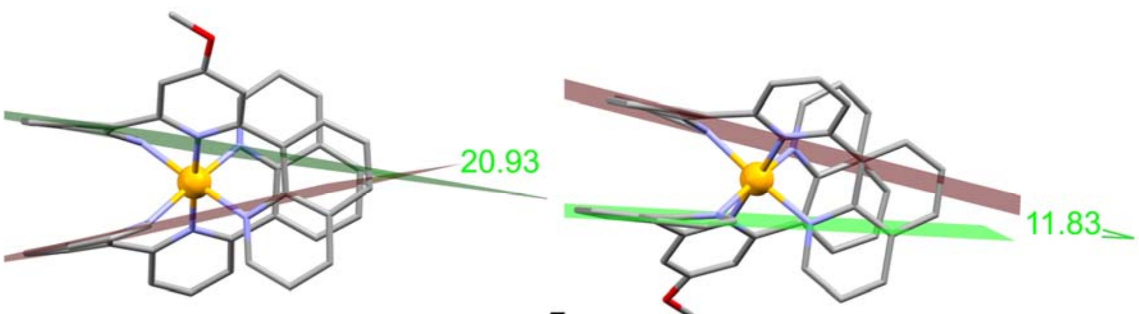

5

Figure S14. Interplanar angle calculated for **5**. Angles are calculated in degrees (°).

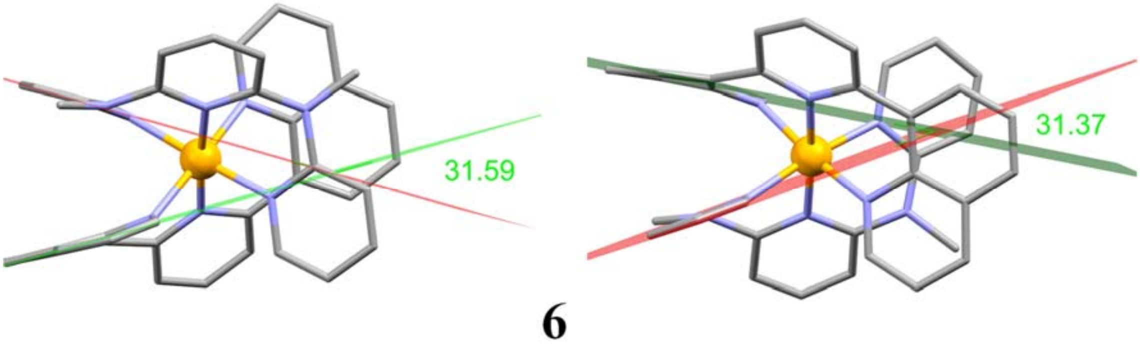

6

Figure S15. Interplanar angle calculated for **6**. Angles are calculated in degrees (°).

Table S14. Assignments and calculations from the absorption spectra. Absorption spectra recorded between $1\cdot10^{-5}$ M and $2\cdot10^{-5}$ M in the UV-vis region and between 3.1 mM and 3.4 mM between 650 and 850 nm at room temperature. Recording done in H_2O for **1-4** and in 1 M HCl in H_2O for H_2-**3** and H_2-**4**.

Compound	λ (nm)	ν (cm^{-1})	ε (M^{-1} cm^{-1})	Assignment [a]	k_{rad} (s^{-1})	τ_{rad} (ms)	f_{exp}	D_{exp} (D^2)
**1**	270	37037	32634	π*← π				
	302	33113	32865	π*← π				
	331	30211	39904	π*← π			---	
	378	26455	15684	π*← π				
	406	24631	[b]	$^4T_2 \leftarrow ^4A_2$				
	699	14306	0.11	$^2T_1$'(3), ^2E'(2) ←$^4A_2$'	31.1	32.2	1.34E-07	1.87E-05
	722	13850	0.08	$^2T_1$'(2) ←$^4A_2$'	16.0	62.3	6.93E-08	1.07E-05
	729	13717	0.35	^2E'(1) ←$^4A_2$'	51.8	19.3	2.24E-07	3.54E-05
	754	13263	0.08	$^2T_1$'(1) ←$^4A_2$'	8.9	112.3	3.85E-08	6.72E-06
**2**	274	36496	27465	π*← π				
	337	29674	36841	π*← π				
	368	27174	34439	π*← π			---	
	412	24272	[b]	$^4T_2 \leftarrow ^4A_2$				
	702	14245	0.12	$^2T_1$'(3), ^2E'(2) ←$^4A_2$'	44.3	22.6	1.91E-07	2.70E-05
	730	13699	0.35	$^2T_1$'(2), ^2E'(1) ←$^4A_2$'	66.5	15.0	2.87E-07	4.56E-05
	755	13245	0.09	$^2T_1$'(1) ←$^4A_2$'	12.6	79.6	5.43E-08	9.52E-06

Compound	λ (nm)	*v* (cm^{-1})	*ε* (M^{-1} cm^{-1})	Assignment [a]	k_{rad} (s^{-1})	τ_{rad} (ms)	f_{exp}	D_{exp} (D^2)
	285	35088	34103	π*← π				
	304	32895	30995	π*← π				
	337	29674	29259	π*← π				
**3**	381	26247	28074	π*← π			---	
	405	24691	[c]	$^4T_2 \leftarrow {}^4A_2$				
	473	21142	24690	CT				
	640	15625	3601	CT				
	269	37175	53401	π*← π				
	316	31646	53512	π*← π				
	380	26316	21221	π*← π			---	
	409	24450	[b]	$^4T_2 \leftarrow {}^4A_2$				
H_2-**3**	699	14302	0.13	$^2E'(2) \leftarrow {}^4A_2'$	43.3	23.1	1.87E-07	2.61E-05
	719	13907	0.04	$^2T_1'(3) \leftarrow {}^4A_2'$	4.4	227.3	1.9E-08	2.89E-06
	728	13733	0.34	$^2T_1'(2) \leftarrow {}^4A_2'$	54.7	18.3	2.36E-07	3.73E-05
	751	13310	0.09	$^2E'(1) \leftarrow {}^4A_2'$	15.2	65.9	6.55E-08	1.13E-05
	768	13019	0.03	$^2T_1'(1) \leftarrow {}^4A_2'$	4.3	233.2	1.85E-08	3.43E-06
	266	37594	41411	π*← π				
	337	29674	45133	π*← π				
**4**	375	26667	41602	π*← π			---	
	402	24876	[c]	$^4T_2 \leftarrow {}^4A_2$				
	470	21277	31223	CT				
	601	16639	6256	CT				

Compound	λ (nm)	v (cm^{-1})	ε (M^{-1} cm^{-1})	Assignment [a]	k_{rad} (s^{-1})	τ_{rad} (ms)	f_{exp}	D_{exp} (D^2)
	272	36765	29784	$\pi^* \leftarrow \pi$				
	336	29762	42556	$\pi^* \leftarrow \pi$			---	
	355	28169	36963	$\pi^* \leftarrow \pi$				
	411	24331	[b]	$^4T_2 \leftarrow {}^4A_2$				
H_2-**4**	686	14571	0.02	$^2E'(2) \leftarrow {}^4A_2'$	4.7	214.4	2.01E-08	2.66E-06
	701	14265	0.12	$^2T_1'(3) \leftarrow {}^4A_2'$	38.7	25.8	1.67E-07	2.35E-05
	720	13893	0.06	$^2T_1'(2) \leftarrow {}^4A_2'$	12.5	80.3	5.38E-08	8.20E-06
	730	13703	0.32	$^2E'(1) \leftarrow {}^4A_2'$	52.6	19.0	2.27E-07	3.61E-05
	753	13273	0.10	$^2T_1'(1) \leftarrow {}^4A_2'$	15.6	64.1	6.74E-08	1.18E-05

[a] Octahedral point groups (O_h) are assumed for the [$Cr^{III}N_6$] chromophores. [b] Not given because of the overlap with LMCT. [c] Not given because of the overlap with LMCT and CT.

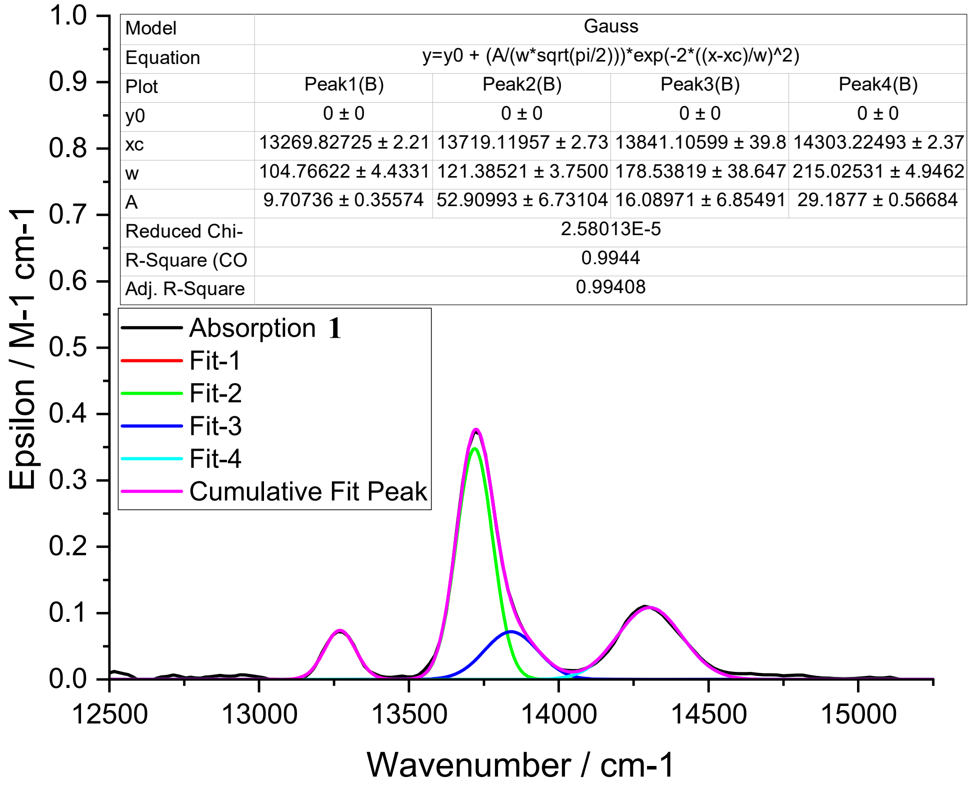

Figure S16. NIR absorption spectra at high concentration in solution of **1** in H_2O at 3.1 mM (black), the four deconvoluted gaussian fitted curves (red, green, dark blue and light blue), the cumulative spectra fitting (pink) and the associated fitting results and parameters.

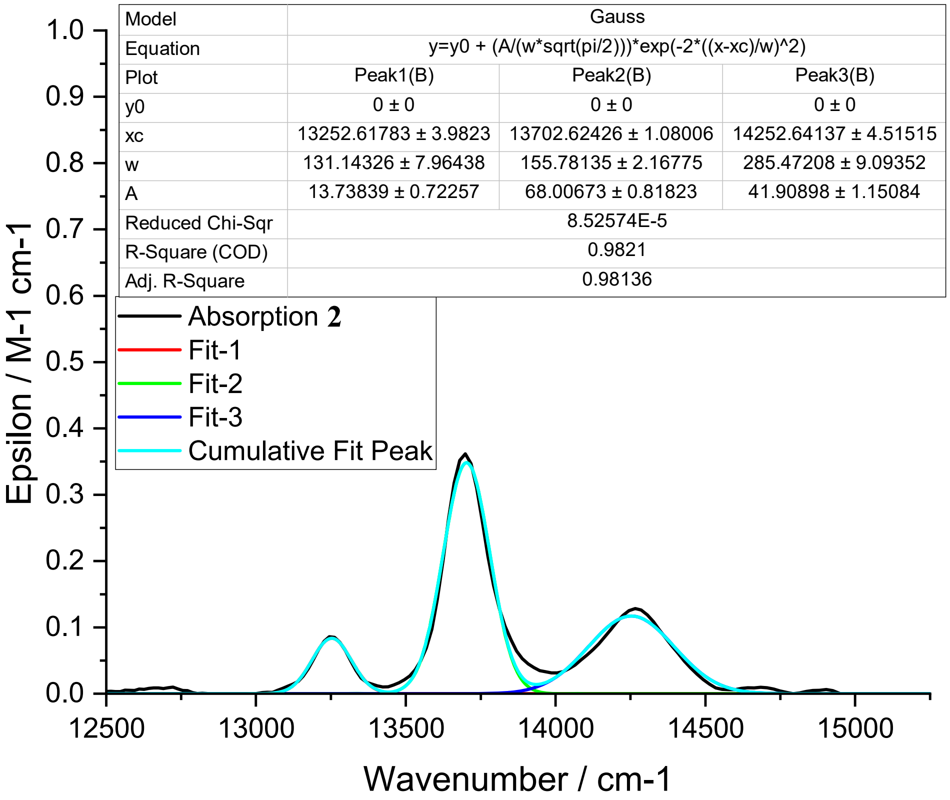

Figure S17. NIR absorption spectra at high concentration in solution of **2** in H_2O at 3.4 mM (black), the three deconvoluted gaussian fitted curves (red, green and dark blue), the cumulative spectra fitting (light blue) and the associated fitting results and parameters.

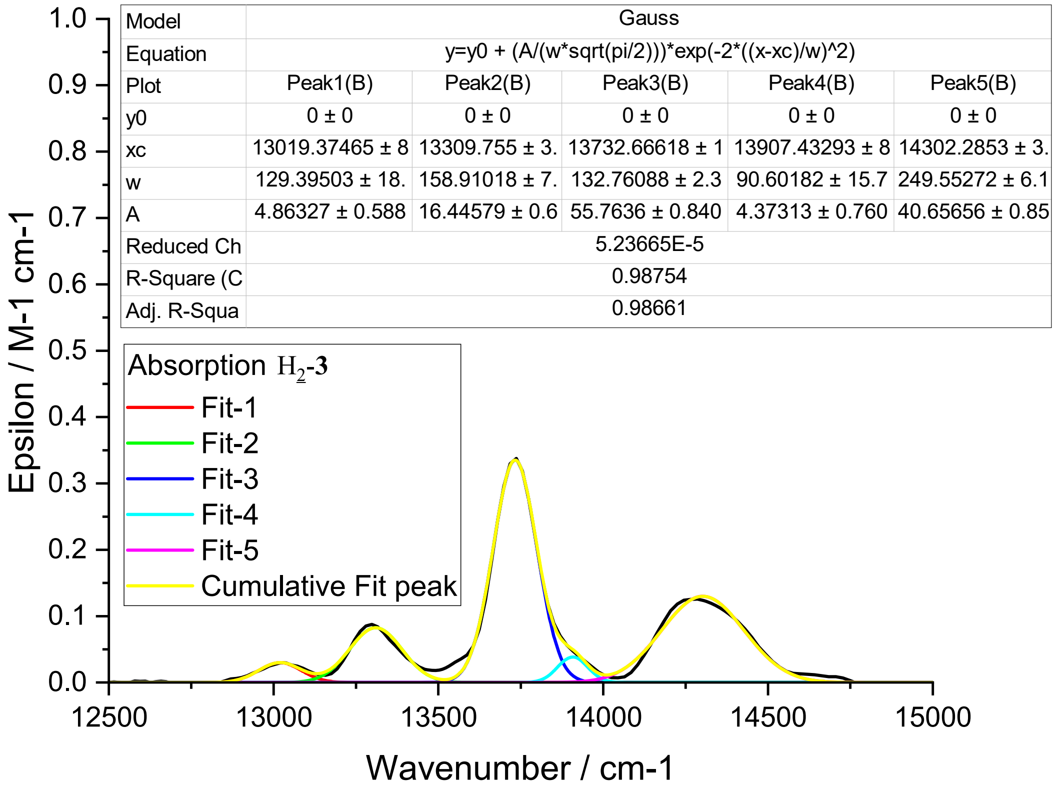

Figure S18. NIR absorption spectra at high concentration in solution of H_2-**3** in H_2O at 3.9 mM (black), the five deconvoluted gaussian fitted curves (red, green, dark blue, light blue and pink), the cumulative spectra fitting (yellow) and the associated fitting results and parameters.

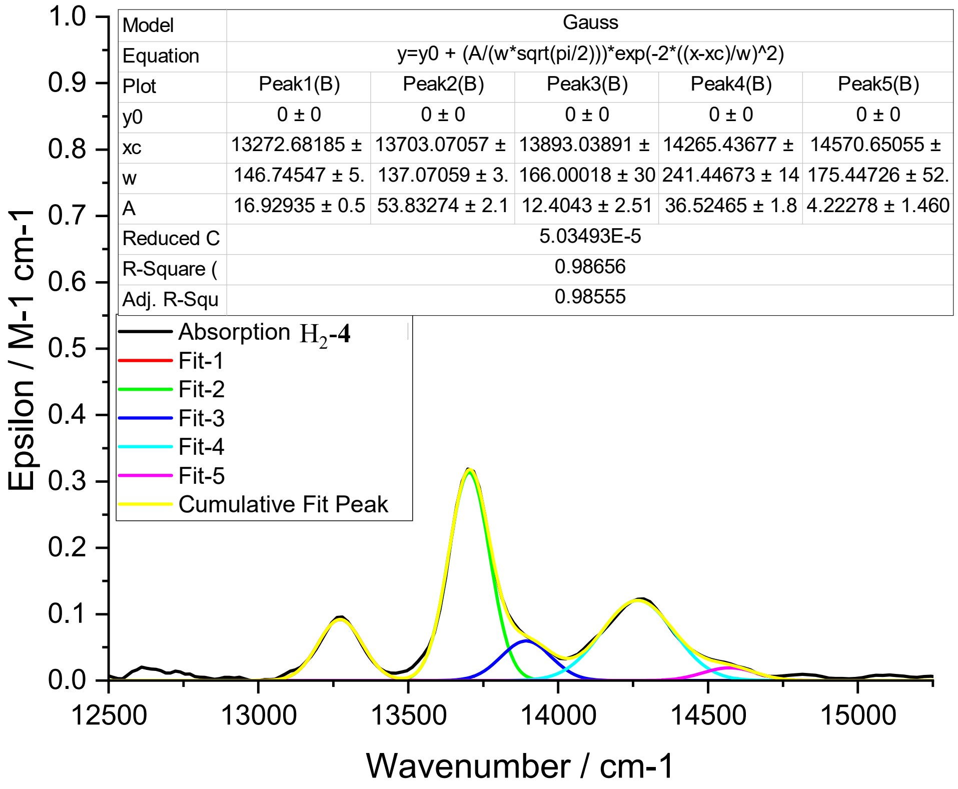

Model	Gauss				
Equation	y=y0 + (A/(w*sqrt(pi/2)))*exp(-2*((x-xc)/w)^2)				
Plot	Peak1(B)	Peak2(B)	Peak3(B)	Peak4(B)	Peak5(B)
y0	0 ± 0	0 ± 0	0 ± 0	0 ± 0	0 ± 0
xc	13272.68185 ±	13703.07057 ±	13893.03891 ±	14265.43677 ±	14570.65055 ±
w	146.74547 ± 5.	137.07059 ± 3.	166.00018 ± 30	241.44673 ± 14	175.44726 ± 52.
A	16.92935 ± 0.5	53.83274 ± 2.1	12.4043 ± 2.51	36.52465 ± 1.8	4.22278 ± 1.460
Reduced C	5.03493E-5				
R-Square (	0.98656				
Adj. R-Squ	0.98555				

Figure S19. NIR absorption spectra at high concentration in solution of H_2-**4** in H_2O at 2.3 mM (black), the five deconvoluted gaussian fitted curves (red, green, dark blue, light blue and pink), the cumulative spectra fitting (yellow) and the associated fitting results and parameters.

Table S15. Racah parameters, nephelauxetic parameter and the energies of the excited states computed for each complex.

Compound	Δ /cm^{-1} [a]	B /cm^{-1}	C /cm^{-1}	C/B	β	^2E /cm^{-1}	$^2T_1$ /cm^{-1}	$^4T_2$ /cm^{-1}	$^2T_2$ /cm^{-1}	$^4T_1$ /cm^{-1}
**1**	24631	655	2735	4.2	0.69	13228	13680	24631	20434	31697
**2**	24272	635	2774	4.4	0.67	13210	13643	24272	20475	31144
**3**	24691	---	---	---	---	--- [b]	--- [b]	---	---	---
H_2-**3**	24450	653	2746	4.2	0.69	13245	13699	24450	20457	31497
**4**	24876	---	---	---	---	--- [b]	--- [b]	---	---	---
H_2-**4**	24331	650	2743	4.2	0.68	13210	13661	24331	20409	31338

[a] Values are extracted from the absorption spectra within 5% accuracy. [b] The energy of Cr($^2T_{1'}$) and Cr(^2E) could not be extracted due to the dominant CT.

Table S16. Kinetic rate constants and emission quantum yields recorded in H_2O at 293 K and in H2O/DMSO (1:1) at 77 K with the exception of H_2-**3** and H_2-**4** that were recorded in 1 M HCl in H_2O at 298 K and 77 K.

Compound	$k_{\mathrm{Cr,rad}}^{^2\mathrm{E}',^2\mathrm{T}'_1}$ ^{a} /s^{-1}	$k_{\mathrm{Cr,nrad}}^{^2\mathrm{E}',^2\mathrm{T}'_1}$ ^{b} /s^{-1}	$\tau_{\mathrm{Cr,obs}}^{^2\mathrm{E}',^2\mathrm{T}'_1}$ ^{c} /µs	$\tau_{\mathrm{Cr,obs}}^{^2\mathrm{E}',^2\mathrm{T}'_1}$ ^{d} /µs	$\tau_{\mathrm{Cr,obs}}^{^2\mathrm{E}',^2\mathrm{T}'_1}$ ^{e} /ms	$\Phi_{\mathrm{Cr}}^{\mathrm{Cr}(^2\mathrm{E}',^2\mathrm{T}'_1)}$ ^{f} /%	$\Phi_{\mathrm{Cr}}^{\mathrm{L}}$ ^{g} /%	$\Phi_{\mathrm{Cr}}^{\mathrm{L}}$ ^{h} /%	$\eta_{\mathrm{sens}}^{\mathrm{L}\to\mathrm{Cr}}$ ^{i} /%	k_q /s^{-1}
**1**	107.9(5)	506(15)	1630(50)	63.73(8)	2.59(2)	17.6(1)	12.4(4)	0.67(1)	71(4)	6.23E+06
**2**	123.3(5)	724(15)	1180(50)	52.02(7)	2.62(2)	14.5(1)	12.4(6)	0.27(1)	85(6)	7.59E+06
**3**	---	---	---	---	1.10(9)	---	---	---	---	---
H_2-**3**	120.5(5)	---	---	---	2.4(2)	---	---	$6(1)\cdot10^{-4}$	---	---
**4**	---	---	---	---	1.31(1)	---	---	---	---	---
H_2-**4**	124.0(5)	---	---	---	2.53(3)	---	---	$1.1(2)\cdot10^{-3}$	---	---

^{a} $k_{\mathrm{Cr,rad}}^{^2\mathrm{E}',^2\mathrm{T}'_1}$ is taken as the sum of all measured k_{rad}. ^{b} $k_{\mathrm{nrad}} = (1/\tau_{\mathrm{obs}}) - k_{\mathrm{rad}}$ (deaerated solution). ^{c} τ_{obs} from time-resolved experiments at 293 K (deaerated solution). ^{d} τ_{obs} from time-resolved experiments at 293 K (aerated solution). ^{e} τ_{obs} from time-resolved experiments at 77 K (aerated solution). ^{f} Intrinsic quantum yield of the specified Cr level $\Phi_{\mathrm{Cr}}^{\mathrm{Cr}} = k_{\mathrm{rad}}\,\tau_{\mathrm{obs}}$ (deaerated solution), ^{g,h} Overall quantum yield $\Phi_{\mathrm{Cr}}^{\mathrm{L}}$ determined by relative method using $[\mathrm{Cr(ddpd)_2}]^{3+}$ (λ_{exc} = 435 nm in acetonitrile; $\Phi_{\mathrm{Cr}}^{\mathrm{L}}$= 12.1%) in deaerated (^{g}) and aerated (^{h}) CH_3CN solutions. ^{i} $\eta_{\mathrm{sens}}^{\mathrm{L}\to\mathrm{Cr}} = \frac{\Phi_{\mathrm{Cr}}^{\mathrm{L}}}{\Phi_{\mathrm{Cr}}^{\mathrm{Cr}(^2\mathrm{E}',^2\mathrm{T}'_1)}}$ (deaerated solution). Lifetime: estimated relative uncertainty ± 10%. Quantum yield: estimated relative uncertainty ±10%.

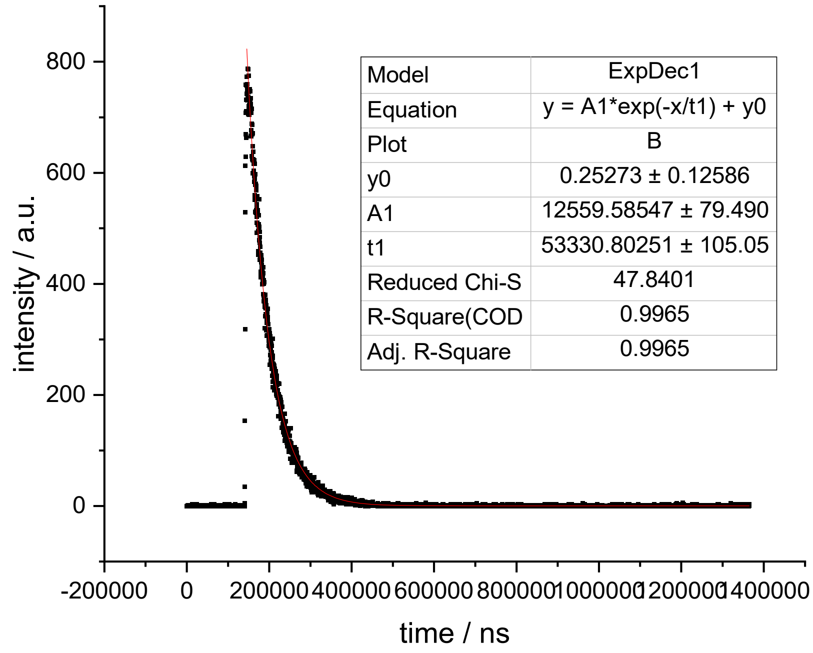

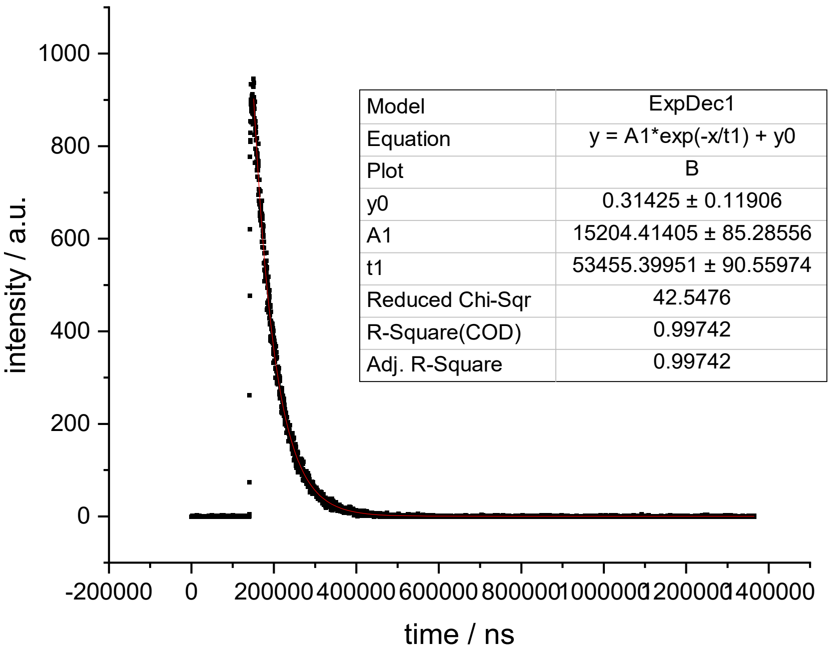

Figure S20. Excited state lifetime fitting for the homoleptic complex **1** (λ_{exc} = 400 nm flash lamp light source (1 μs pulse, HORIBA Scientific)): Cr($^2T_1$') (top, 731 nm) and Cr(^2E') (bottom, 755 nm) lifetimes in aerated aqueous solution at 293 K.

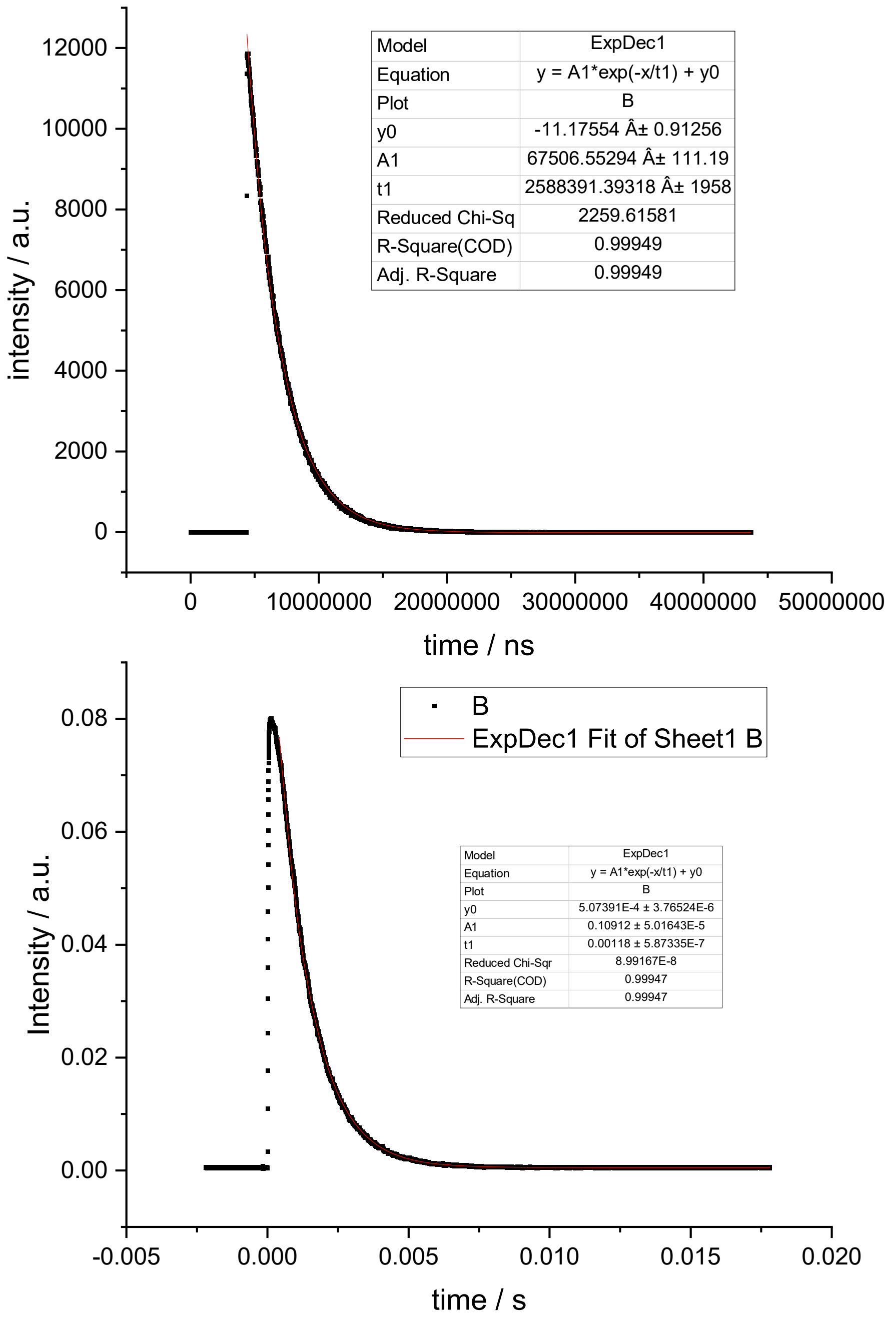

Figure S21. Excited state lifetime fitting for the homoleptic complex **1**: Cr($^2T_1$',^2E') lifetimes in: Top: deaerated acetonitrile solution (λ_{exc} = 350 nm flash lamp light source (1 µs pulse, HORIBA Scientific)). Bottom: at 77 K in a H_2O/DMSO (1:1) solution (λ_{exc} = 355 nm Nd:YAG).

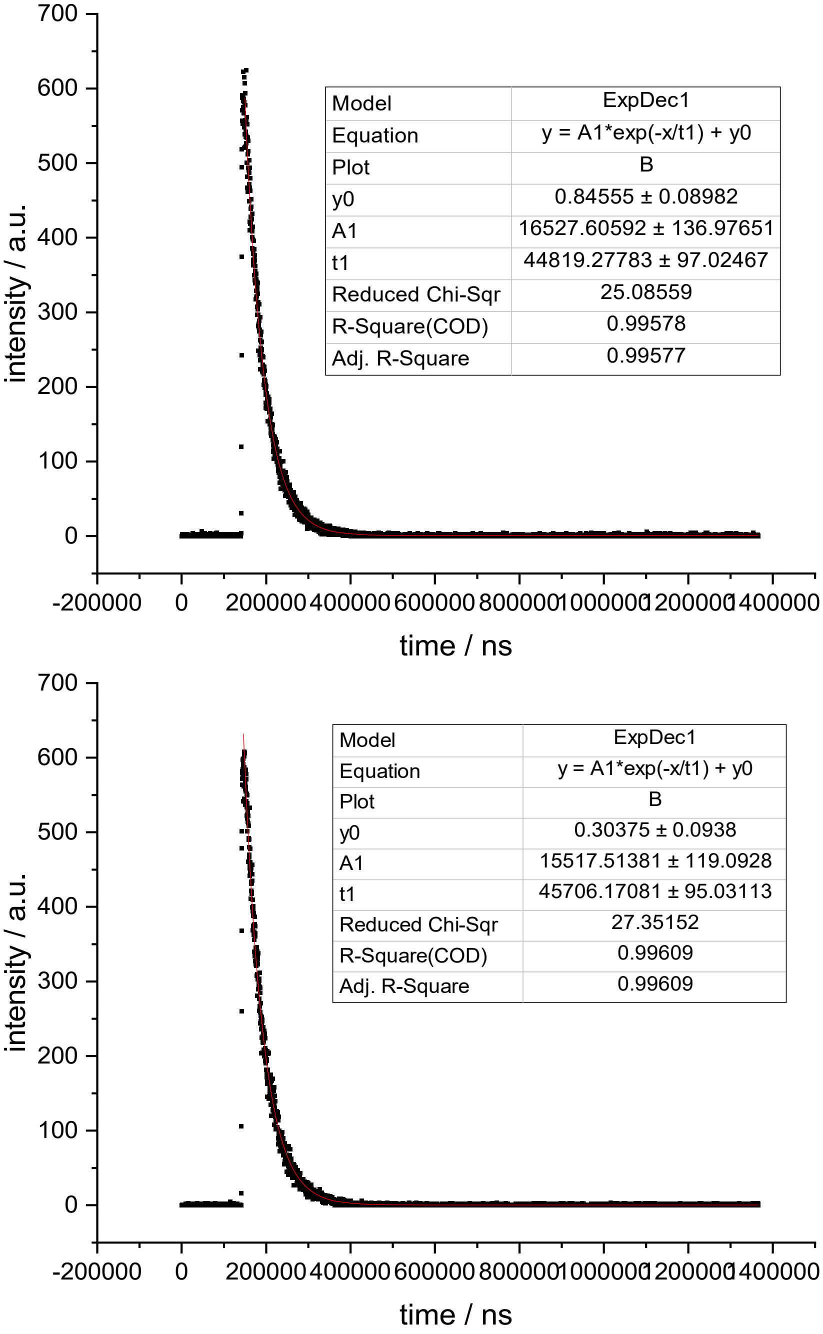

Figure S22. Excited state lifetime fitting for the homoleptic complex **2** (λ_{exc} = 400 nm, flash lamp light source (1 μs pulse, HORIBA Scientific)): Cr($^2T_1$’) (top, 732 nm) and Cr(^2E’) (bottom, 756 nm) lifetimes in aerated aqueous solution at 293 K.

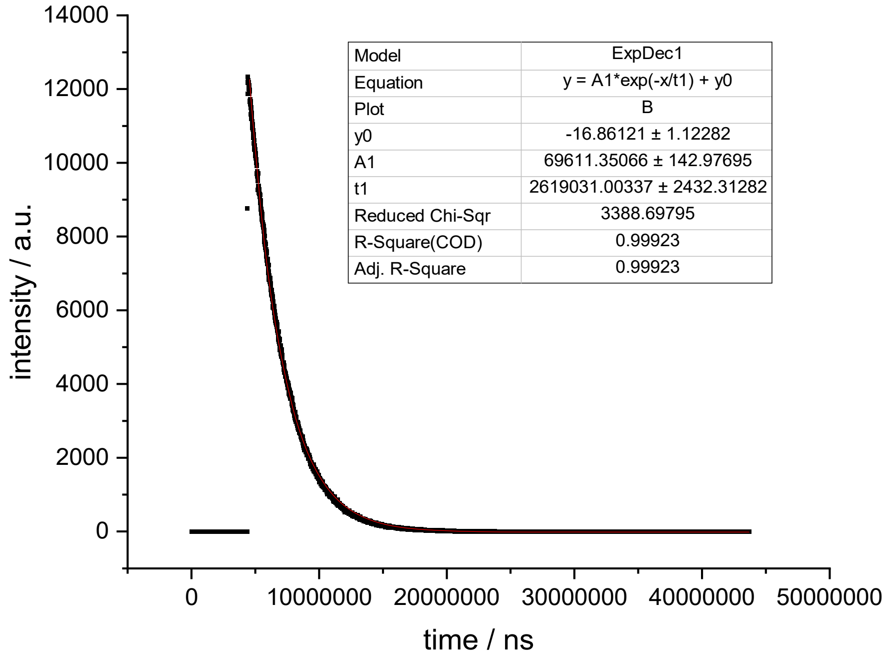

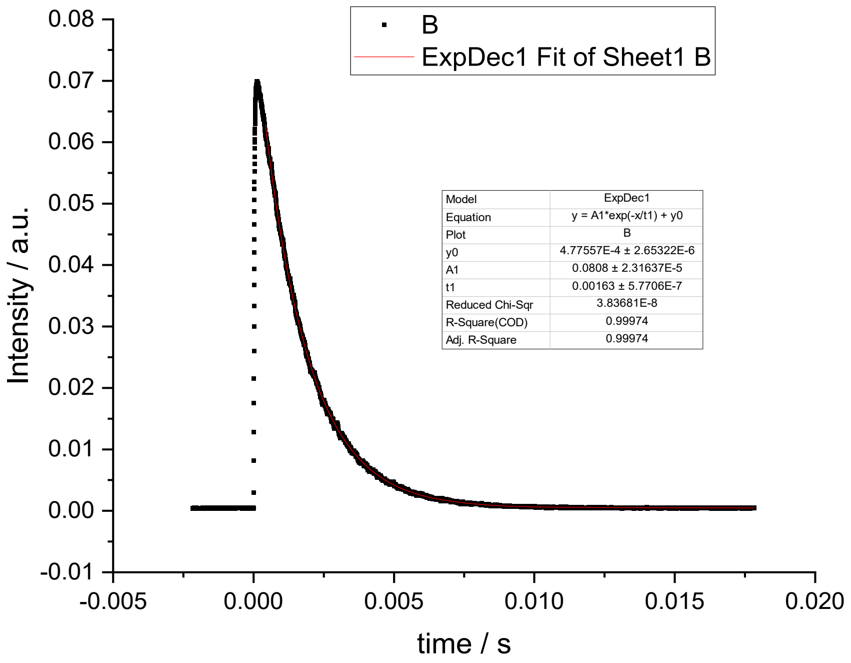

Figure S23. Excited state lifetime fitting for the homoleptic complex **2**: Cr($^2T_1$',^2E') lifetimes in: Top: deaerated acetonitrile solution (λ_{exc} = 350 nm flash lamp light source (1 μs pulse, HORIBA Scientific)). Bottom: at 77 K in a H_2O/DMSO (1:1) solution (λ_{exc} = 355 nm Nd:YAG).

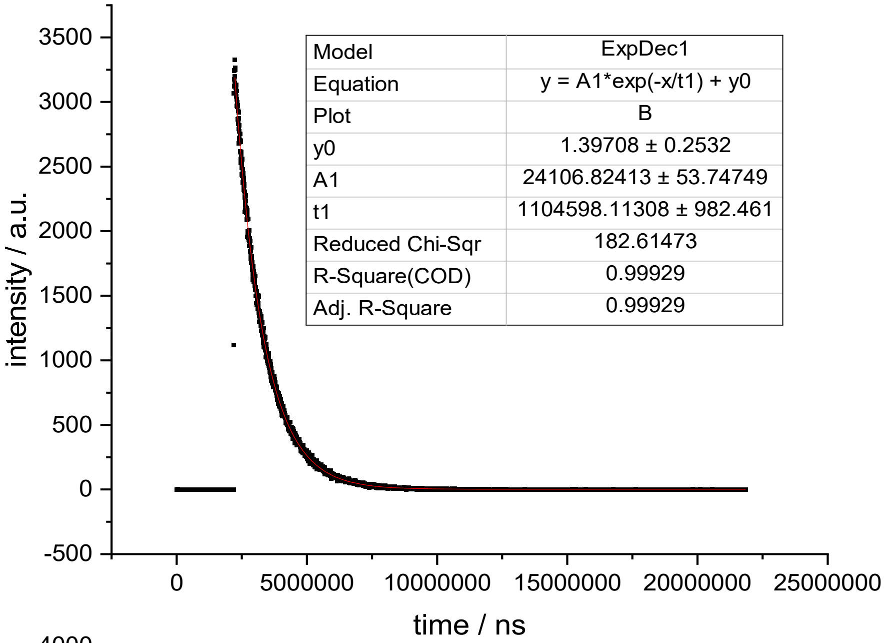

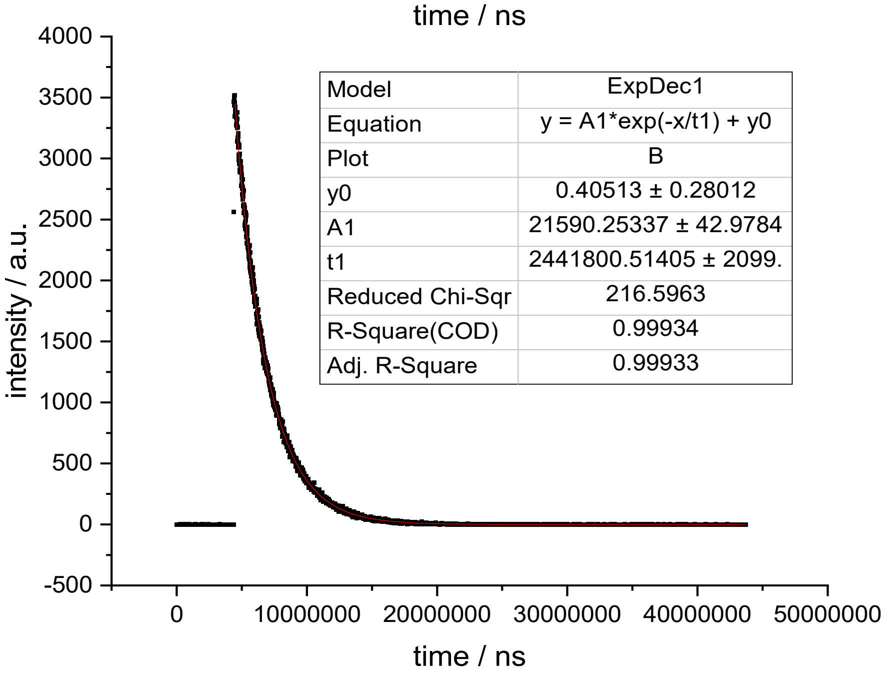

Figure S24. Excited state lifetime fitting for the homoleptic complex **3** and H_2-**3**: Cr($^2T_1$’,^2E’) lifetimes (λ_{exc} = 480 nm flash lamp light source (1 μs pulse, HORIBA Scientific)) of: Top: **3** in a H_2O/DMSO (1:1) solution at 77 K. Bottom: **7-DMAH** in a 1M HCl in H_2O/DMSO (1:1) solution at 77 K.

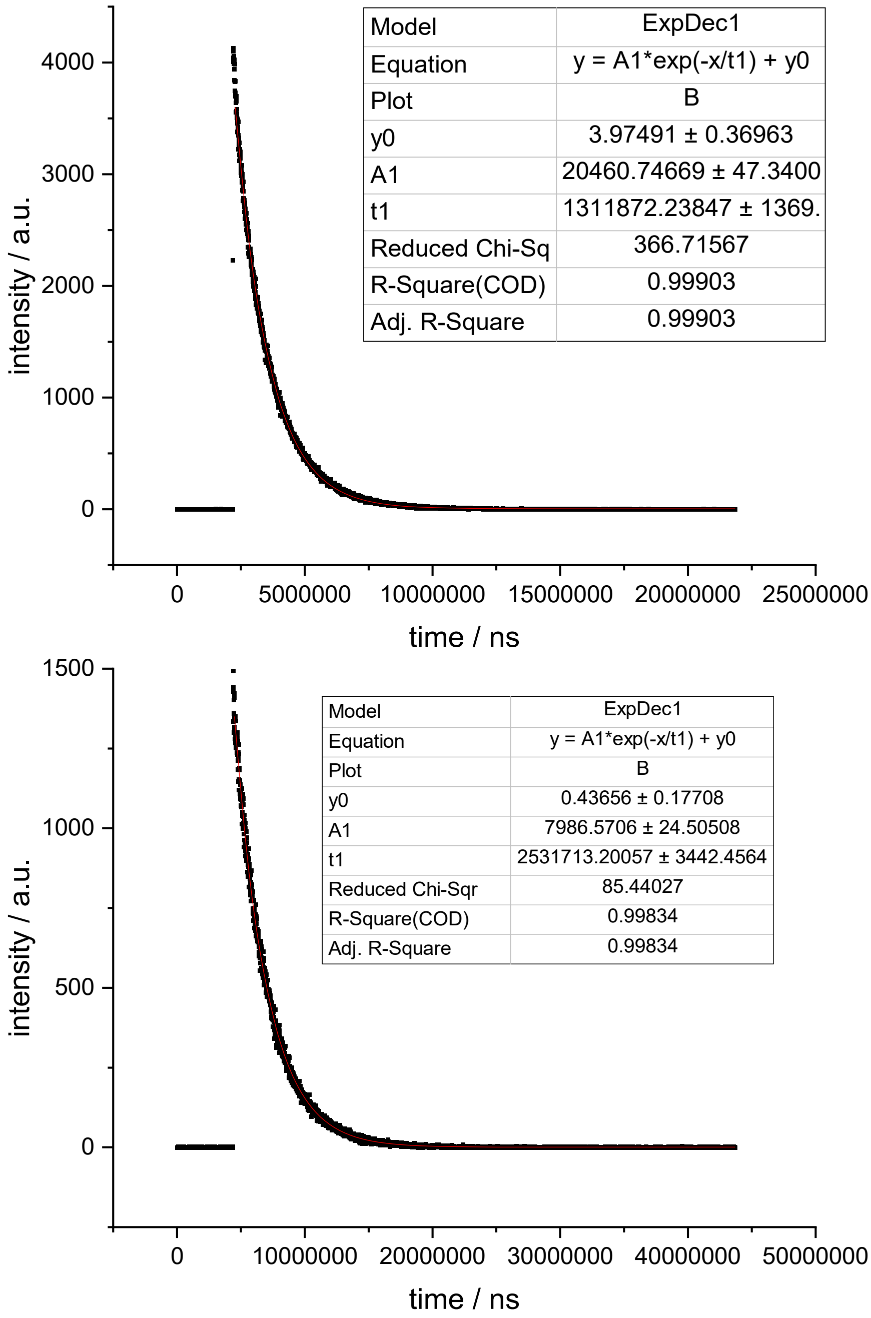

Figure S25. Excited state lifetime fitting for the homoleptic complex **4** and H_2-**4**: Cr($^2T_1$’,^2E’) lifetimes (λ_{exc} = 500 nm flash lamp light source (1 μs pulse, HORIBA Scientific)) of: Top: **4** in a H_2O/DMSO (1:1) solution at 77 K. Bottom: H_2-**4** in a 1M HCl in H_2O/DMSO (1:1) solution at 77 K.

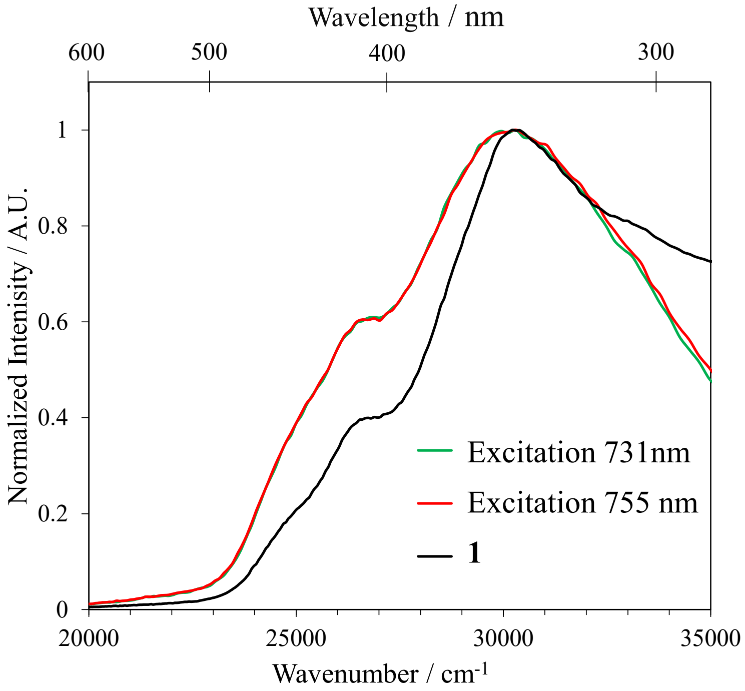

Figure S26. Excitation spectra of **1**. The excitation spectrum was recorded in H_2O at ~10^{-4} M. The absorption spectrum is displayed in black.

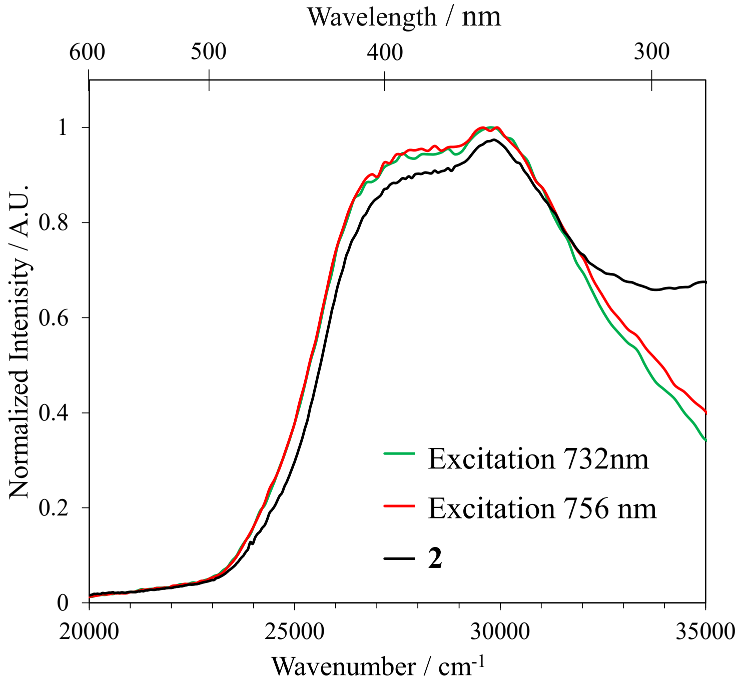

Figure S27. Excitation spectra of **1**. The excitation spectrum was recorded in H_2O at ~10^{-4} M. The absorption spectrum is displayed in black.

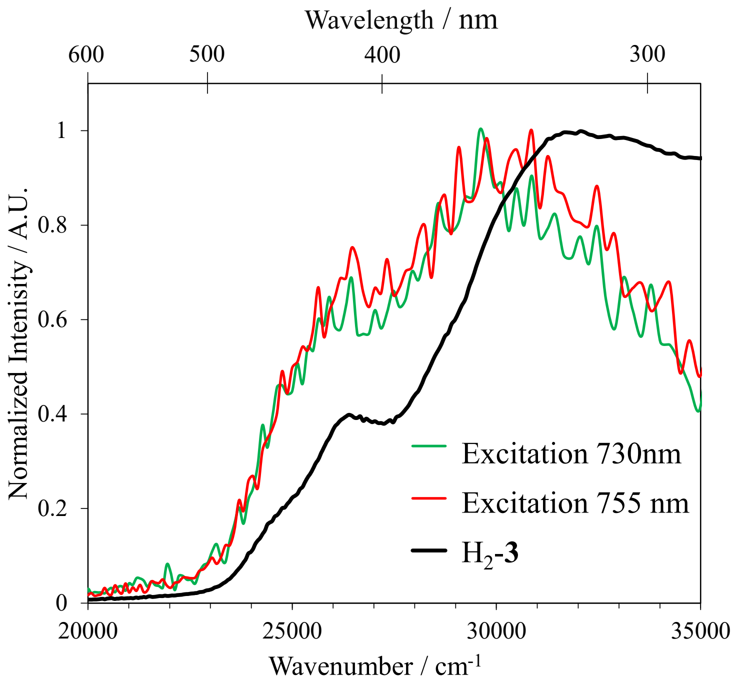

Figure S28. Excitation spectra of H_2-**3**. The excitation spectrum was recorded in 1 M HCl in H_2O at ~10^{-4} M. The absorption spectrum is displayed in black.

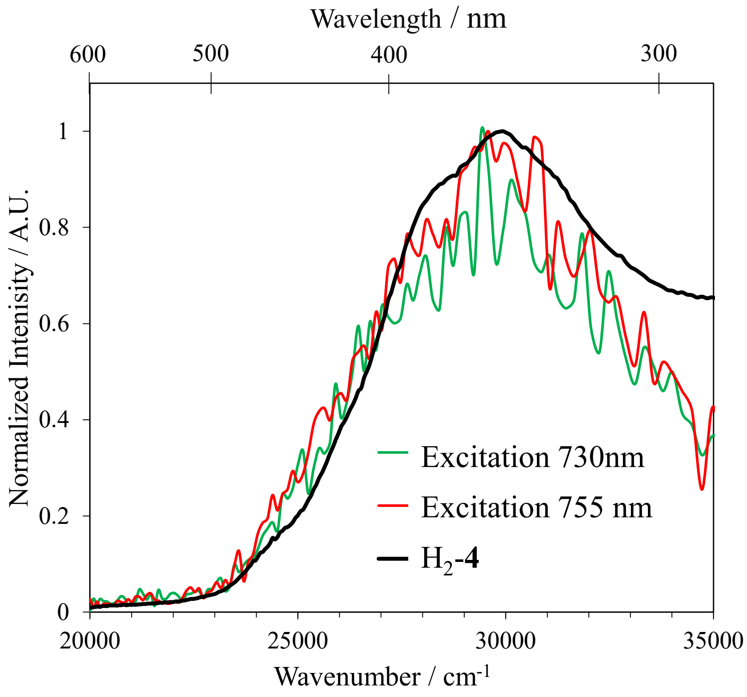

Figure S29. Excitation spectra of H_2-**4**. The excitation spectrum was recorded in 1 M HCl in H_2O at ~10^{-4} M. The absorption spectrum is displayed in black.

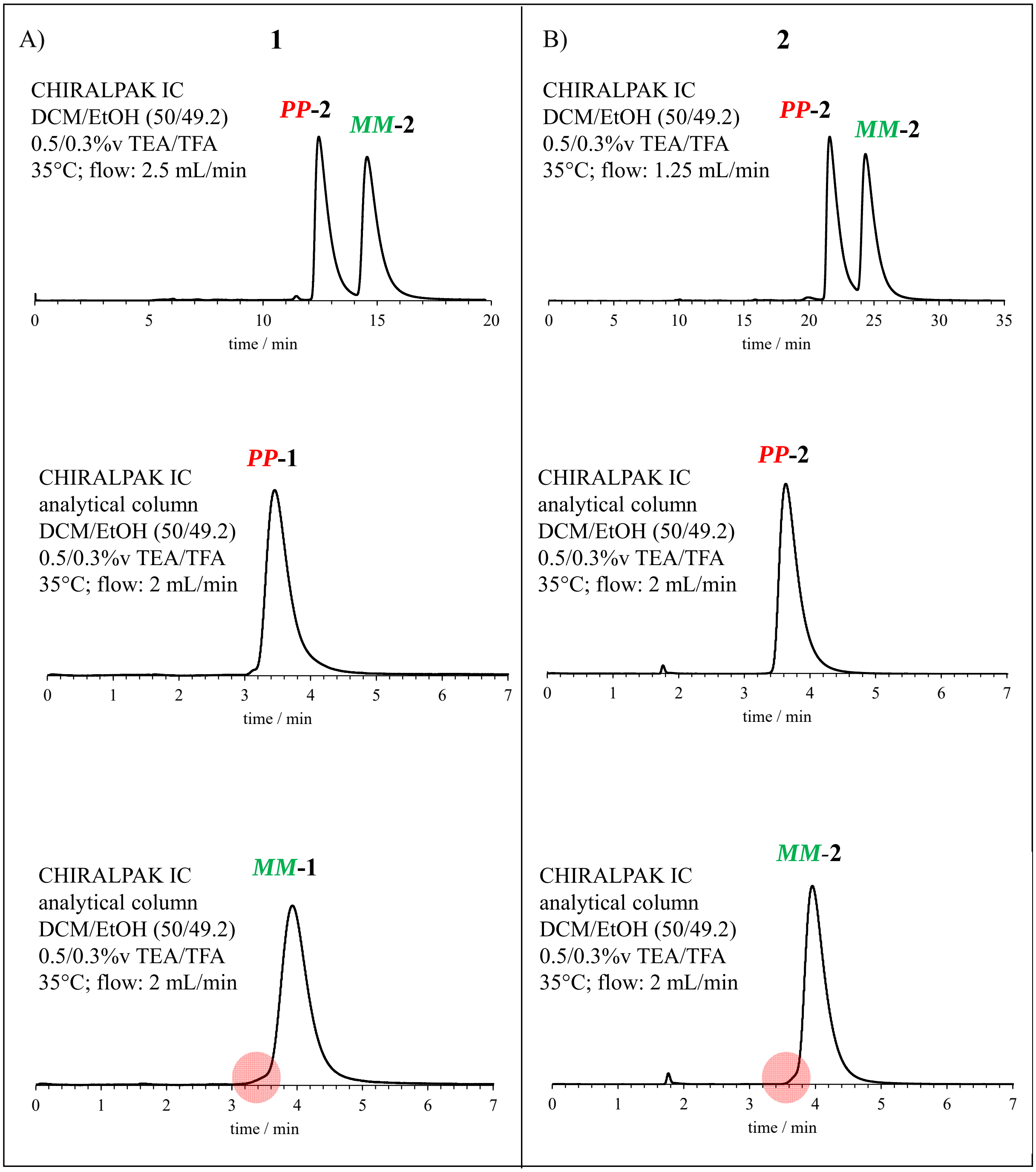

Figure S30. CSP HPLC chromatograms of enantiomerically resolved complexes (1st row, semi-preparative column) and the analytical chromatogram of the reinjection of the collected fraction to check the enantiomeric excess (2nd row for *PP* enantiomers, 3rd row for *MM* enantiomers). Each column is representing a complex: A) **1**, B) **2**. The red areas highlight the small contamination of the *PP* enantiomer in the collected *MM* fraction of *MM*-**1** and *MM*-**2**.

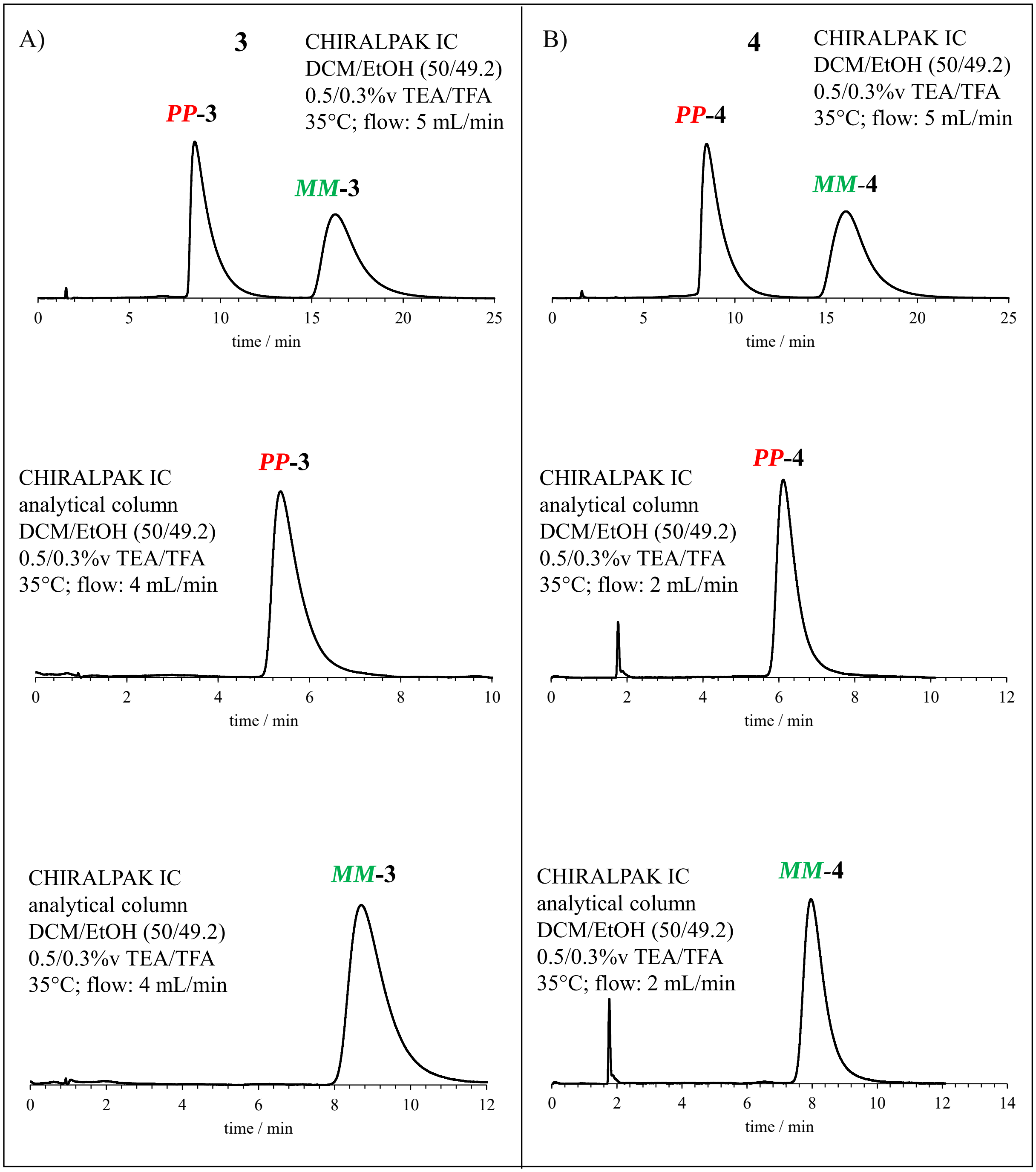

Figure S31. CSP HPLC chromatograms of enantiomerically resolved complexes (1st row, semi-preparative column) and the analytical chromatogram of the reinjection of the collected fraction to check the enantiomeric excess (2nd row for *PP* enantiomers, 3rd row for *MM* enantiomers). Each column is representing a complex: A) **3**, B) **4**.

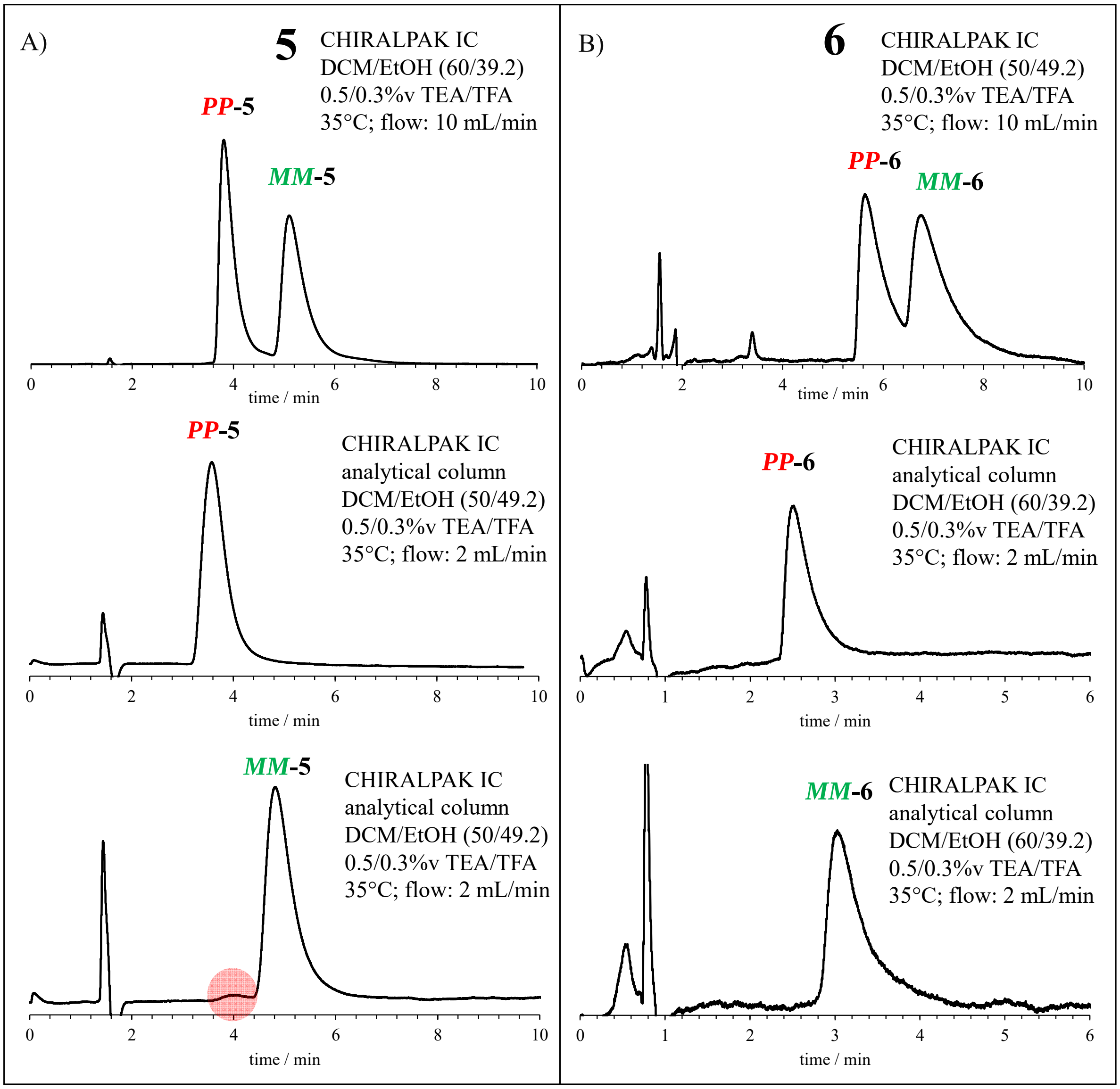

Figure S32. CSP HPLC chromatograms of enantiomerically resolved complexes (1st row, semi-preparative column) and the analytical chromatogram of the reinjection of the collected fraction to check the enantiomeric excess (2nd row for *PP* enantiomers, 3rd row for *MM* enantiomers). Each column is representing a complex: A) **5**, B) **6**. The red areas highlight the small contamination of the *PP* enantiomer in the collected *MM* fraction of *MM*-**5**.

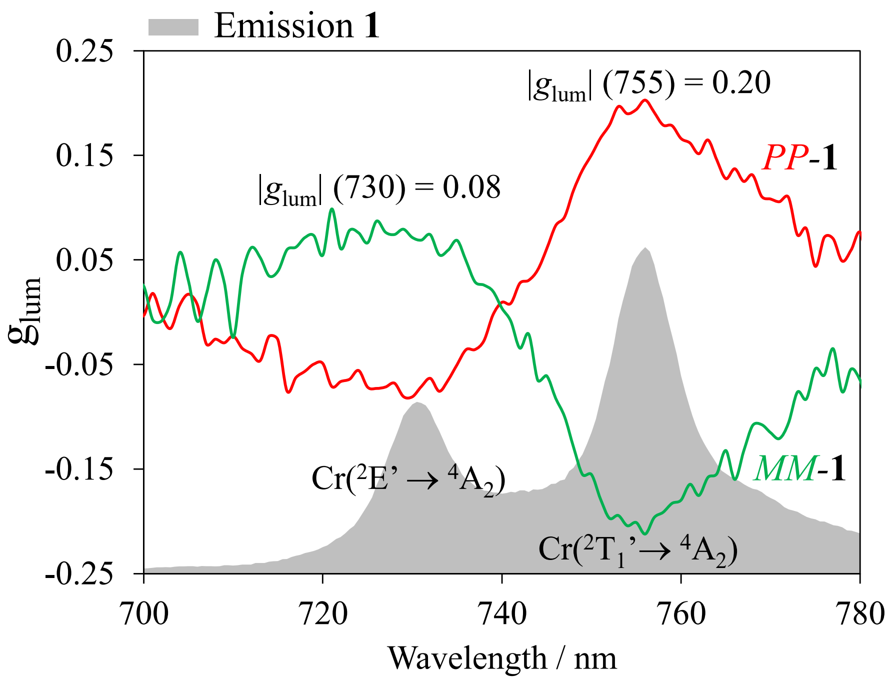

Figure S33. Circularly polarized luminescence spectra of the two enantiomers *PP*-**1** (red) and *MM*-**1** (green) in CH3CN, displayed as dissymmetry factor g_{lum}. The grey area is the emission spectra of the corresponding racemic complex (λ_{exc} = 340 nm, experimental bandwidth = 0.5 nm).

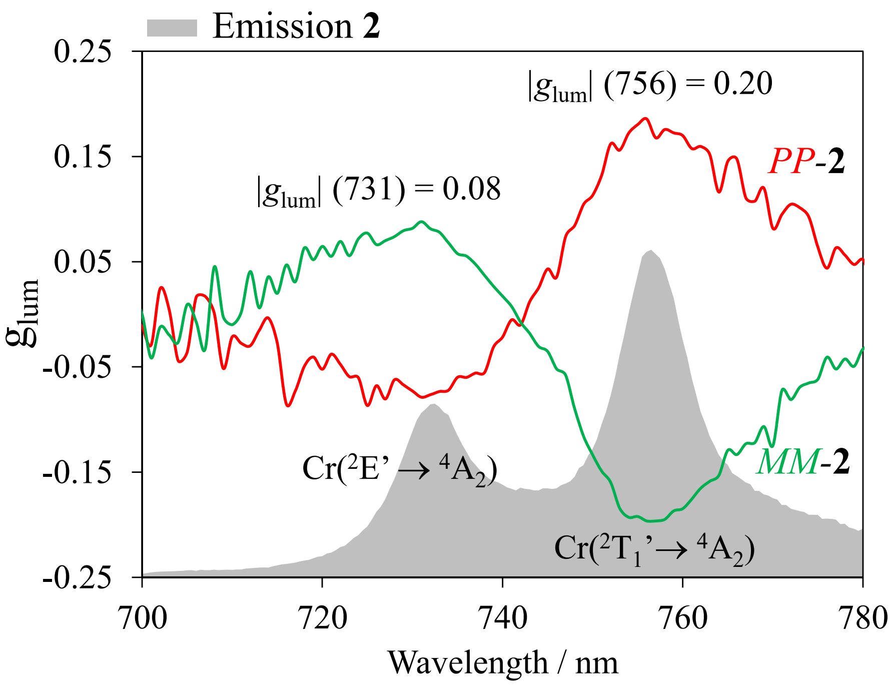

Figure S34. Circularly polarized luminescence spectra of the two enantiomers *PP*-**2** (red) and *MM*-**2** (green) in CH3CN, displayed as dissymmetry factor g_{lum}. The grey area is the emission spectra of the corresponding racemic complex (λ_{exc} = 340 nm, experimental bandwidth = 0.5 nm).

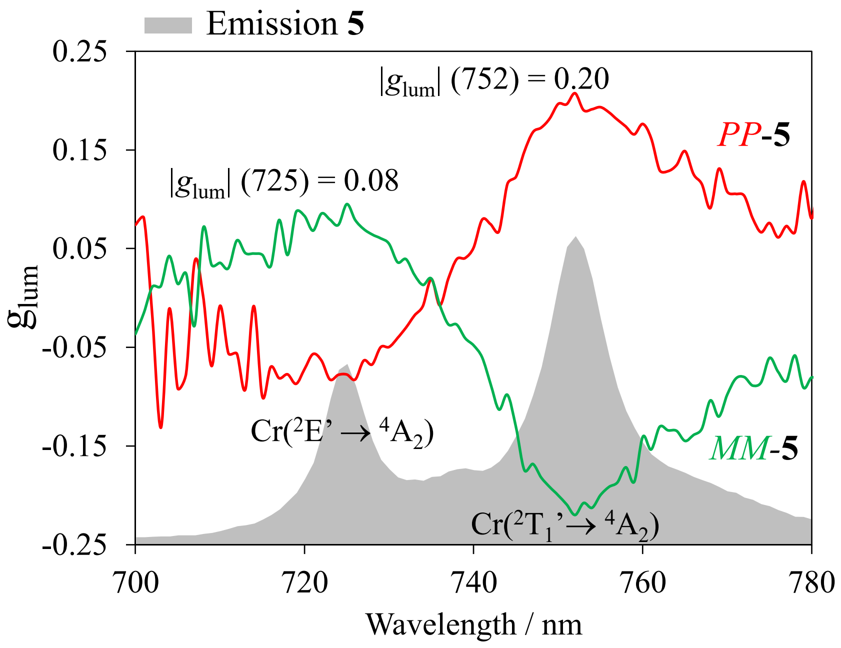

Figure S35. Circularly polarized luminescence spectra of the two enantiomers *PP*-**5** (red) and *MM*-**5** (green) in CH_3CN, displayed as dissymmetry factor g_{lum}. The grey area is the emission spectra of the corresponding racemic complex (λ_{exc} = 340 nm, experimental bandwidth = 0.5 nm).

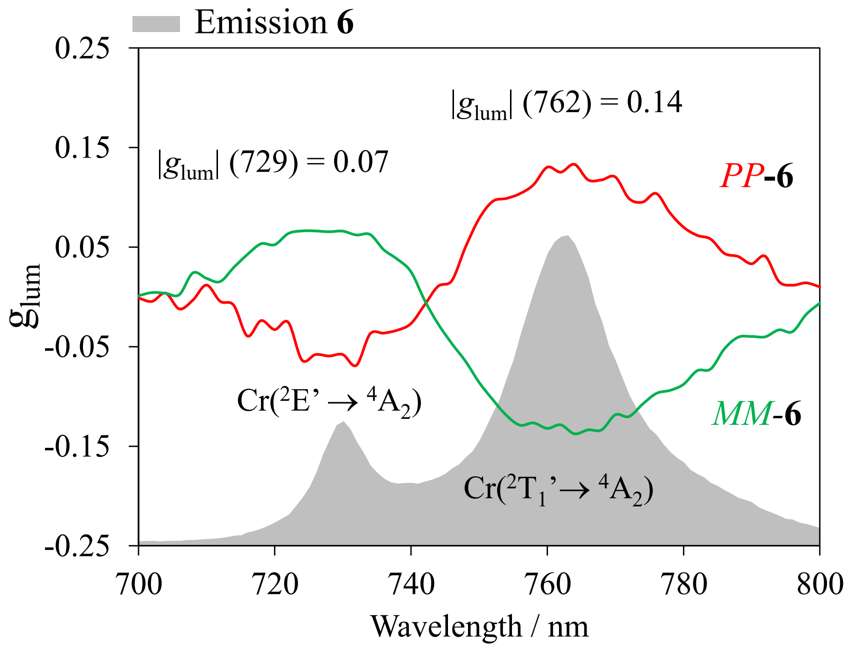

Figure S36. Circularly polarized luminescence spectra of the two enantiomers *PP*-**6** (red) and *MM*-**6** (green) in CH_3CN, displayed as dissymmetry factor g_{lum}. The grey area is the emission spectra of the corresponding racemic complex (λ_{exc} = 340 nm, experimental bandwidth = 5 nm).

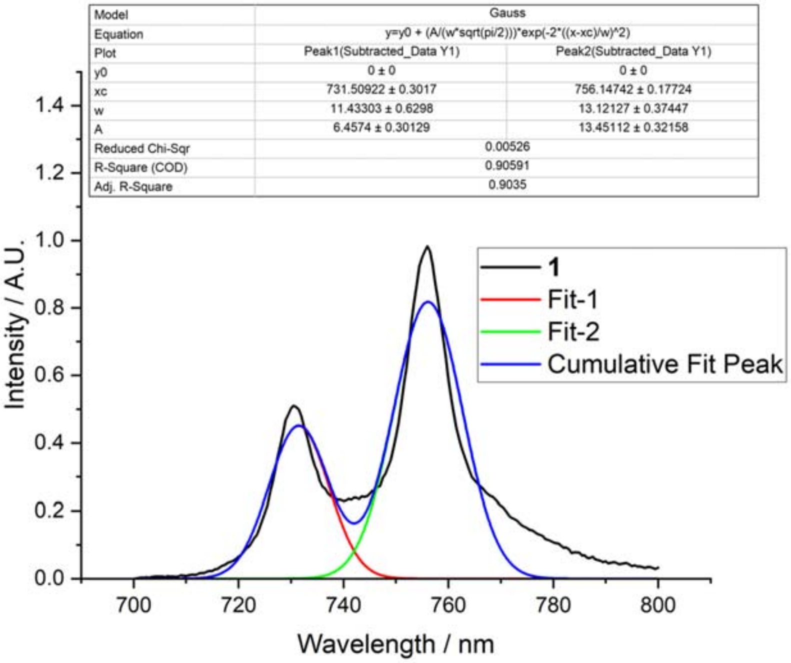

Figure S37. Deconvolution of the emission spectra of **1** in two gaussian curves (red, green), the cumulative spectra fitting (blue) and the associated fitting results and parameters for the calculation of B_{CPL}.

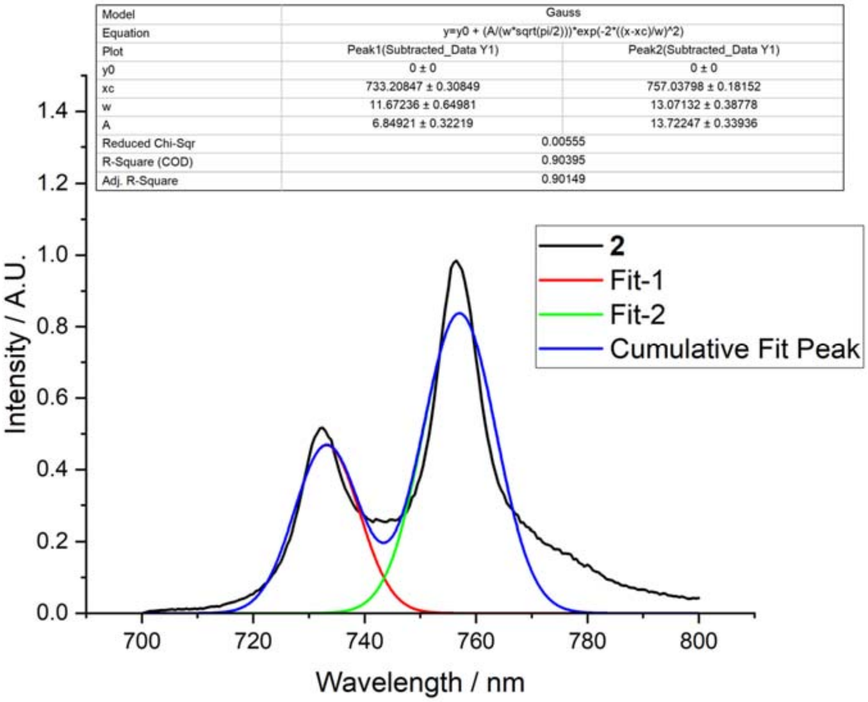

Figure S38. Deconvolution of the emission spectra of **2** in two gaussian curves (red, green), the cumulative spectra fitting (blue) and the associated fitting results and parameters for the calculation of B_{CPL}.

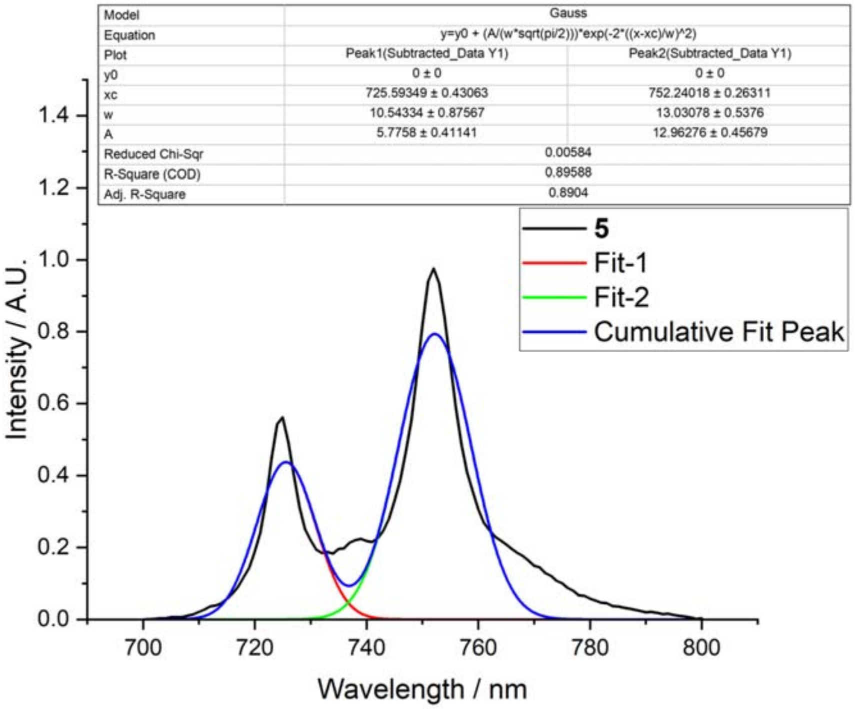

Figure S39. Deconvolution of the emission spectra of **5** in two gaussian curves (red, green), the cumulative spectra fitting (blue) and the associated fitting results and parameters for the calculation of B_{CPL}.

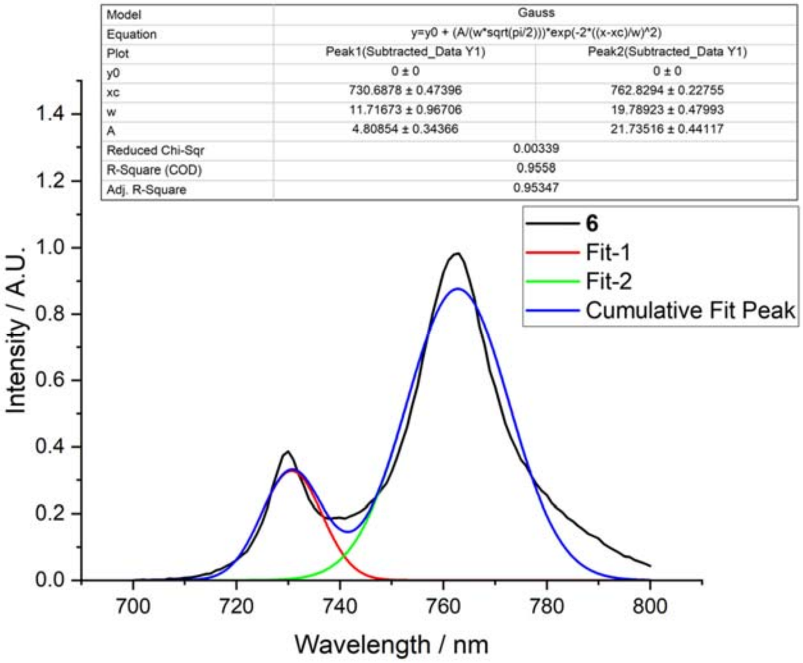

Figure S40. Deconvolution of the emission spectra of **6** in two gaussian curves (red, green), the cumulative spectra fitting (blue) and the associated fitting results and parameters for the calculation of B_{CPL}.
